# Mechanistic Insights Into the Retention and Separation Mechanism of Poly(Ethylene Glycol)‐Modified Short Oligonucleotides in Anion‐Exchange Chromatography

**DOI:** 10.1002/biot.70183

**Published:** 2026-01-26

**Authors:** Noriko Yoshimoto, Tomoya Matsumoto, Yoshiatsu Ono, Yuma Kumagai

**Affiliations:** ^1^ Department of Applied Chemistry Yamaguchi University Yamaguchi Japan

**Keywords:** Anion exchange chromatography, HETP, Mass‐transfer analysis, PEGylated oligonucleotides, Retention mechanism

## Abstract

Purification is a critical step in the development of synthetic short DNA for pharmaceutical applications. PEGylation enhances stability and pharmacokinetics but introduces steric and hydration effects that affect chromatographic behavior without altering the nominal DNA charge. This study investigated the retention and mass‐transfer behavior of thymine‐based poly(dT) oligomers (9–95 bases) modified with poly(ethylene glycol) (PEG) using anion‐exchange chromatography with a mechanistic ion‐exchange framework. By systematically varying PEG molecular weight and modification site, we examined how PEG‐induced changes in molecular size and local interaction environment are reflected in mechanistic retention parameters. PEGylation shifted elution to lower salt concentrations, with more pronounced effects observed for shorter oligomers and mid‐position modifications. Model‐based analysis revealed that the effective number of binding sites remained unchanged after PEG modification, indicating preserved charge‐based binding stoichiometry. In contrast, PEGylation reduced the ion‐exchange equilibrium constant, reflecting changes in the local interaction environment. HETP analysis showed that unmodified poly(dT) exhibited a strong retention‐dependent decrease in plate height, whereas this dependence was weaker for PEGylated DNA, suggesting that PEGylation modifies intraparticle mass‐transfer characteristics through combined steric hindrance and charge‐shielding effects. These results provide mechanistic insights into the chromatographic behavior of PEGylated oligonucleotides and a rational basis for optimizing their purification.

## Graphical Abstract and Lay Summary

Mechanistic analysis revealed unchanged binding‐site stoichiometry (*B*) and altered interaction strength (*K*_e_) for PEGylated DNA.

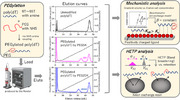

**Abbreviations**
AIEC: Anion‐exchange chromatography
HETP: height equivalent to a theoretical plate
LGE: Linear gradient elution
NHS: *N*‐hydroxysuccinimide
PEG: Poly(ethylene glycol)
SEC: Size‐exclusion chromatography

## 1 Introduction

In recent years, advances in oligonucleotide synthesis have enabled the production of structurally diverse nucleic acid molecules at scales relevant to practical applications [1, 2, 3]. Concurrently, rapid progress in nucleic‐acid‐based therapeutics and functional DNA technologies has highlighted the advantages of chemically modified nucleic acids in terms of stability, functionality, and pharmacokinetic properties [4, 5, 6, 7, 8]. Consequently, the demand for industrial‐scale DNA separation processes has increased [1, 9, 10, 11, 12, 13], and the separation of chemically modified DNA is expected to become an increasingly important challenge.

Among available separation techniques, anion‐exchange chromatography (AIEC) is widely used for DNA separation because it directly exploits electrostatic interactions between negatively charged nucleic acids and positively charged stationary phases [1, 12, 13, 14, 15, 16, 17]. DNA retention in AIEC is governed by the effective number of binding sites simultaneously interacting with anion‐exchange ligands, as described by the Yamamoto model [18, 19, 20, 21]. The corresponding *B* values, which cannot be directly inferred from the nominal charge of the molecule, can be experimentally determined from chromatographic elution behavior and provide a practical and mechanistically meaningful parameter for describing DNA separation. Recently, a detailed mechanistic ion‐exchange model has been proposed to theoretically estimate the effective number of binding sites for oligonucleotides by explicitly accounting for solution chemistry and charge regulation effects [12]. This approach demonstrated that DNA binding behavior can be rationalized beyond simple charge‐based assumptions. These theoretical developments provide a mechanistic basis for interpreting *B* values as physically meaningful parameters that reflect the interaction environment of both native and chemically modified DNA in solution.

PEGylation is one of the most successful strategies in nucleic‐acid‐based therapeutics [4, 6, 8] and is of particular interest because poly(ethylene glycol) (PEG) is electrically neutral and primarily introduces steric and hydration effects without altering the nominal charge of DNA [6, 22, 23]. Although AIEC has been applied to the separation of PEGylated proteins, quantitative interpretation of retention behavior is often complicated by their structural complexity [24, 25, 26, 27, 28, 29, 30, 31, 32, 33, 34, 35]. In contrast, DNA, with its simple and predictable backbone, provides a well‐defined model system for elucidating the effects of PEG conjugation [22] on retention behavior in AIEC.

In our previous work, we established an experimental framework for analyzing DNA retention in AIEC by applying the Yamamoto model to elution profiles of short DNAs [18, 19, 20, 21]. This framework enabled quantitative characterization of retention behavior across poly(dT) sequences of varying chain lengths and provided a practical basis for predicting elution conditions under linear gradient elution.

In this study, we investigated the separation behavior of PEGylated DNA prepared by conjugating activated PEG to either the terminal (5′‐end) or a mid‐position thymine of poly(dT) using AIEC with a multi‐porous particulate resin and a monolithic column, employing our previously established mechanistic framework. To evaluate the steric and electrostatic‐shielding effects of PEGylation, rather than charge reduction, PEG was introduced at either the oxygen atom of the 5′‐phosphate group or the C5 methyl group of a thymine base in the middle position of poly(dT), ensuring that the number of negatively charged phosphates remained unchanged after PEGylation. Furthermore, we examined mass‐transfer properties in AIEC to clarify how PEG‐induced changes in molecular size and local interaction environment are reflected in mechanistically defined retention parameters [21], rather than being interpreted solely by apparent size effects. This study contributes to a deeper understanding of PEGylated DNA behavior in AIEC and provides insights for optimizing separation processes in pharmaceutical and therapeutic applications.

## 2 Experimental

### 2.1 Chemicals

2‐Amino‐2‐(hydroxymethyl)‐1,3‐propanediol (commercial name: Sigma 7–9, catalog no. T1378) was obtained from Sigma‐Aldrich (St. Louis, USA) for buffer preparation. Polyethylene glycol (PEG) derivatives with *N*‐hydroxysuccinimide (NHS) active esters were purchased from NOF (Kawasaki, Japan), including methoxy‐PEG5000‐(CH_2_)_5_COOH‐NHS (PEG5K, SUNBRIGHT ME‐050HS, *M*_w_ = 5000 g·mol^−^^1^), methoxy‐PEG10000‐(CH_2_)_5_COOH‐NHS (PEG10K, SUNBRIGHT ME‐100HS, *M*_w_ = 10,000 g·mol^−^^1^), and methoxy‐PEG20000‐(CH_2_)_5_COOH (PEG20K, SUNBRIGHT ME‐200HS, Mw = 20,000 g·mol^−^^1^). Synthetic DNAs of poly (dT) including dT_9_ (*M*_w_ = 2676 g·mol^−1^), dT_20_ (*M*_w_ = 6027 g·mol^−1^), dT_50_ (*M*_w_ = 15153 g·mol^−1^), dT_90_ (*M*_w_ = 27321 g·mol^−1^), and dT_95_ (*M*_w_ = 28837 g·mol^−1^) were obtained from Tsukuba Oligo Service (Tsukuba, Japan) or FASMAC (Kanagawa, Japan) as the RPLC‐purified products. These DNAs were modified with a C6‐amino linker at either the 5′‐phosphate group (E‐NH_2_ series) or at a mid‐position thymine base (M‐NH2 series) (see Table 1).

**TABLE 1:** Amino‐linked poly(dT) modified at 5′‐end and mid‐position^a^.

Sample name	Oligonucleotide sequence (5′‐3′)
**amino‐linked poly(dT) modified at the 5′‐end (E‐NH_2_ series)**	
E‐NH_2_‐9T	amino‐C_6_‐dT dT_8_
E‐NH_2_‐20T	amino‐C_6_‐dT dT_19_
E‐NH_2_‐50T	amino‐C_6_‐dT dT_49_
E‐NH_2_‐95T	amino‐C_6_‐dT dT_94_
**amino‐linked poly(dT) modified at a mid‐position thymine (M‐NH_2_ series)**	
M‐NH_2_‐9T	d**T_4_**‐amino‐C_6_‐dT dT_4_
M‐NH_2_‐20T	d**T_9_‐**amino‐C_6_‐dT dT_10_
M‐NH_2_‐50T	d**T_24_**‐amino‐C_6_‐dT dT_25_
M‐NH_2_‐90T	d**T_44_**‐amino‐C_6_‐dT dT_45_

^a^ corresponding structural representations are shown in Figure 1.

**FIGURE 1:**
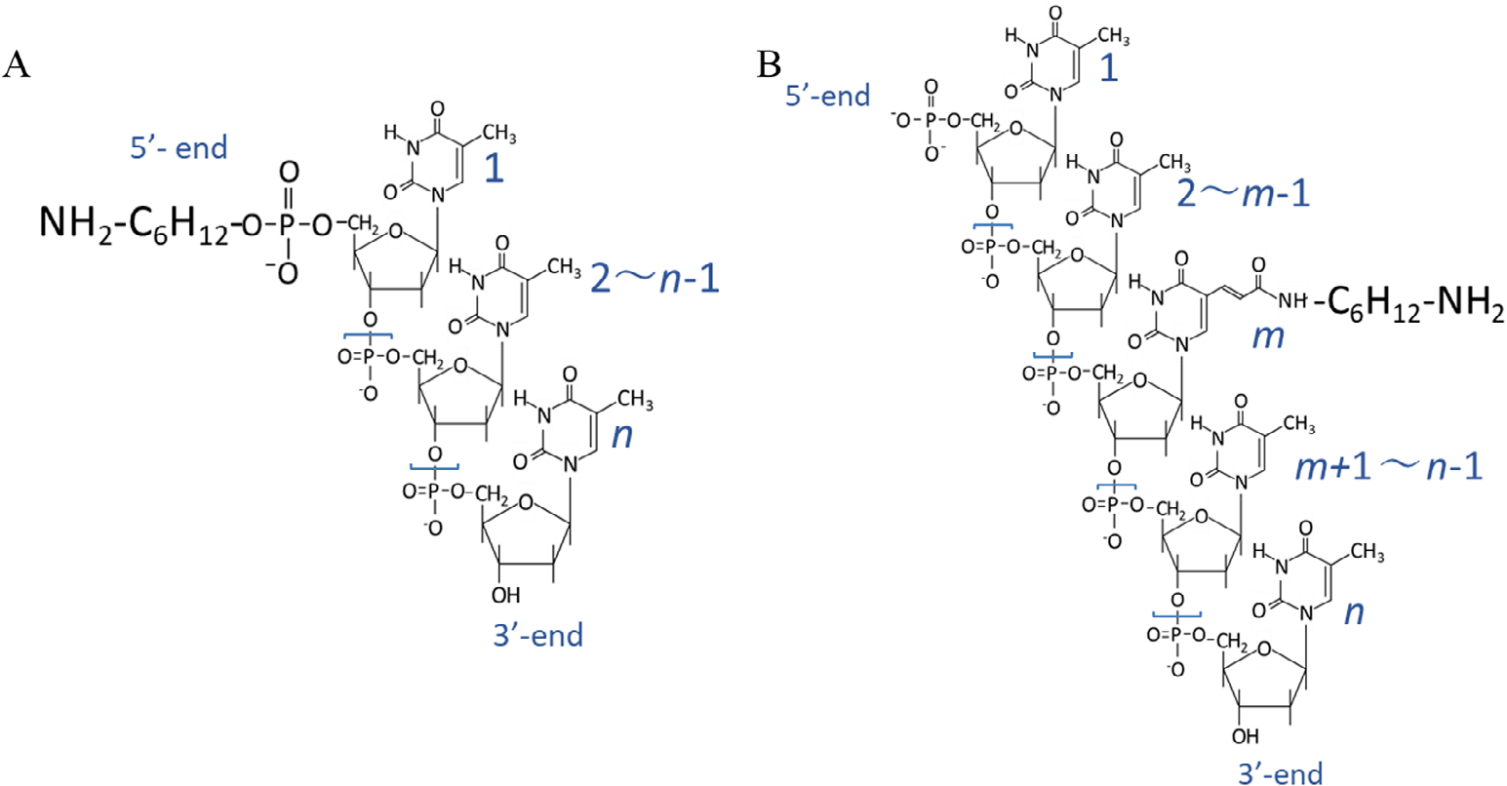
Structures of amino‐linked poly(dT) (A) E‐NH_2_ series with 5′‐end modification and (B) M‐NH_2_ series with mid‐position thymine modification (see Table 1 for details). *n* represents the total number of bases and *m* indicates the number of modified bases.

The position of the C6‐amino modification in M‐NH_2_ series DNA, as *m*, is shown in Figure 1. In this study, poly(dT) sequences with varying lengths and modifications were labeled according to their modification site. For example, 9‐mer poly(dT) with an amino linker at the 5′‐end was denoted E‐NH_2_‐9T, whereas a modification at an internal thymine base was labeled M‐NH_2_‐9T (see Table 1). PEGylated DNA (9T), conjugated to PEG5K at the 5′‐end (E), was designated E‐PEG5K‐9T. Other DNAs, conjugated to PEG of varying molecular weights at the 5′‐end, were abbreviated accordingly.

Prepacked Q Sepharose High Performance (Q HP) column (column A) was obtained from Repligen (Waltham, MA, USA). Q Sepharose HP resin was purchased from Cytiva (Tokyo, Japan) and self‐packed into a plastic tube according to the manufacturer's instructions (column B). The QA CIM monolith was obtained from BIA Separations and mounted onto the column holder (column C). The dimensions of each column used are shown in Table 2. All reagents were used without further purification.

**TABLE 2:** Anion exchange chromatography columns used in this study.

	Packing material	*I.D*. [cm]	*Z* ^a^ [cm]	ε ^b^	*V*_t_ ^c^ [mL]	*d*_p_ ^d^ [µm]	*d*_pore_ ^e^ [nm]	*Λ* ^f^ [meq/mL]
Column A	Q Sepharose HP (prepacked)	0.5	5.0	0.29	1.0	34	50 [36]	0.140 [36]
Column B	Q Sepharose HP (self‐packed)	1.0	5.0	0.30	3.9			
Column C	CIM QA monolith standard disk	1.2	0.3	0.62	0.34	N/A	2000 [37]	0.173 [37]

^a^ *Z*: column length.

^b^ ε: total column bed porosity.

^c^ *V*_t_: total column volume.

^d^ *d*_p_: particle diameter of resin.

^e^ *d*_pore_: pore diameter of resin.

^f^ *Λ*: ionic capacity of the resin.

### 2.2 PEGylation of Poly(dT)

PEGylation was performed via an NHS ester‐amine reaction between NHS‐activated PEG and the C6‐amino linker (denoted as NH_2_‐T) at the 5′‐end of the E‐NH_2_ series or at the internally modified thymine base of the M‐NH_2_ series.

NH_2_‐T was obtained in powder form and dissolved in 1 mL of pure water or a 10 mM sodium phosphate buffer (pH 7) containing 30 mM NaCl to prepare a stock solution with an absorbance at 260 nm of 4–16. The stock solution was then diluted in 10 mM sodium phosphate buffer (pH 7) containing 30 mM NaCl (Table S1, S2 in the Supporting Information), followed by annealing at 100°C for 5 min and cooling at 4°C for 5 min.

Subsequently, PEG with NHS groups was added to the NH_2_‐T solution, and the reaction was conducted for 24 h at room temperature (∼25°C) with continuous mixing.

The resulting PEGylated DNAs were named by modification site and molecular weight, followed by length, e.g., E‐PEG5K‐9T (PEG 5 kDa at the 5′‐end of 9T) and M‐PEG5K‐9T (PEG5K at a mid‐position thymine of 9T) (see Table 1 and Figure 1).

The molecular radius, *r*_m_ of PEGylated poly(dT) was estimated using size‐exclusion chromatography (SEC) with a TSKgel G3000PW_XL_ column, based on a calibration curve generated with PEG (Figures S1–S6 and Table S3 in the Supporting Information).

### 2.3 Linear Salt Gradient Elution (LGE) on Anion Exchange Chromatography

LGE experiments were performed using an ÄKTA pure, Cytiva, Tokyo, Japan. The salt concentration was linearly varied from 30 mM to 1 M using two buffers: 10 mM sodium phosphate buffer solution containing 30 mM NaCl (pH 7, buffer A) and 10 mM sodium phosphate buffer solution containing 1 M NaCl (pH 7, buffer B). Column was equilibrated with buffer A for at least 3 column volumes before sample injection. The electrical conductivity and pH of the eluate were monitored to ensure they matched those of buffer A before loading. A 100 µL sample was injected for each LGE run.

The initial and final salt concentrations, 𝐼_0_ and 𝐼 _f_, gradient volume, 𝑉_g_, the ratio of the gradient volume to the column solid phase volume, *GH* and the elution salt concentration, *I*_R_ for each LGE experiment are summarized in Tables S4, S5 in the Supporting Information.

The *GH* value was calculated using the following equation.

(1) $$\mathrm{GH}=\frac{I_{f}-I_{0}}{V_{g}/V_{t}\left(1-\varepsilon \right)}=g\left(V_{t}-V_{0}\right)$$

In Equation (1), *V*_t_ and *V*_0_ are the column volume and column void volume, respectively. *g* is the salt concentration gradient per elution volume, ε is the void fraction of the column.

For comparison of the *I*_R_ on Q Sepharose HP and monolith columns, column A and column C were selected, respectively.

### 2.4 Distribution Coefficient in LGE

The distribution coefficient, *K*_R_, in LGE was obtained from the *GH*‐*I*_R_ curves, as reported in our previous study [18, 20, 38]. Using the *GH*‐*I*_R_ model, *GH* can be expressed as following equation.

(2) $$\mathrm{GH}=\frac{I_{R}^{B+1}}{A\left(B+1\right)}$$

In Equation (2), *A* is a parameter composed of the ion exchange capacity, Λ, of the resin used and the equilibrium constant *K*_e_ of the ion‐exchange reaction, while *B* is the stoichiometric coefficient of the solute, representing the number of binding sites per biomolecule to the ion‐exchange groups.

The parameter *A* is defined as follows.

(3) $$A=K_{e}\Lambda^{B}$$

The distribution coefficient *K*_R_ as a function of the mobile phase salt concentration in LEG is given by:

(4) $$K_{R}=A\cdot {I_{R}}^{-B}+K^{\prime }$$

where *K*’ is the distribution coefficient of salt.

### 2.5 HETP Measurements in LGE

For the height equivalent to a theoretical plate (HETP) measurements [39], 9T, 20T, 30T, and 50T modified at the 5′‐end with NH_2_ (no PEG moiety), PEG5k, or PEG20k were used, with a self‐packed Q sepharose HP column (Column B). LGE experiments were conducted at flow rates ranging from 0.03 to 2 mL/min. The HETP and diffusion coefficients were determined as reported in in our previous study [20, 21]. The HETP was corrected by a factor *L* to account for peak shrinking and broadening effects in LGE chromatography using the following equation.

(5) $${\mathrm{HETP}}_{\mathrm{LGE}}=\frac{Z}{L^{2}}{\left(\frac{\sigma_{V}}{V_{R}}\right)}^{2}$$

In Equation (5), *Z* is the column length and σ_V_ is the volume‐based standard deviation of the peak. *L* was obtained using a dimensionless group, *M*, defined in terms of *K*_R_ and *B*, based on the *GH*‐*I*_R_ model, as follows:

(6) $$L=\left\{\begin{array}{c} \sqrt{M}\;\left(M<0.25\right)\\ \frac{3.22M}{\begin{array}{c} 1+3.13M \end{array}}\;\left(0.25<M<12\right)\\ 1\;(12<M) \end{array}\right.$$

where:

(7) $$M=\frac{1}{2}\left(\frac{1+HK_{R}}{1+HK^{\prime }}\right)\left(\frac{B+1}{B}\right)$$

The retention volume, *V*_R_ for HETP_LGE_ was determined using *K*_R_ at the peak elution salt concentration *I*_R_:

(8) $$V_{R}=V_{0}\left(1+HK_{R}\right)$$

where *H* is the phase ratio (= (1‐ε) / ε).

### 2.6 Molecular Dynamics Simulations for Local Interaction Analysis of PEGylated Poly(dT)

MD simulations were performed to analyze local interaction tendencies between PEG chains and DNA functional groups for PEGylated 9T modified with PEG (*M*_w_ = 0.6 kDa) at the 5′‐end (E‐PEG‐9T) and PEGylated 9T modified with PEG (*M*_w_ = 0.6 kDa) at the fifth thymine (M‐PEG‐9T) (Figure S21 in the Supporting Information). Detailed simulation protocols and analysis procedures are described in the Supporting Information.

## 3 Results and Discussion

### 3.1 Molecular Radius of PEGylated Oligonucleotides

**FIGURE 2:**
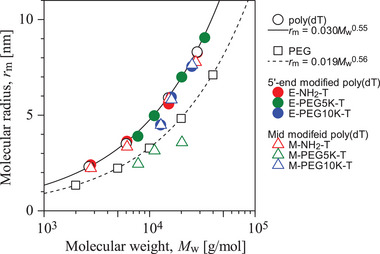
Molecular radius, *r*_m_ as a function of molecular weight, *M*_W_ for unmodified and PEGylated poly(dT). Unfilled circles: unmodified poly(dT); unfilled squares: PEG. Filled circles: amino‐linked poly(dT) and PEGylated poly(dT) modified with the 5′‐end modification (E‐NH_2_‐T, E‐PEG5K‐T, E‐PEG10K‐T). Unfilled triangles: amino‐linked poly(dT) and PEGylated poly(dT) modified with mid‐position modification (M‐NH_2_‐T, M‐PEG5K‐T, M‐PEG10K‐T). The solid line represents the fitted curve for poly(dT) and the dashed line represents the calibration curve for PEG.

The relationship between the molecular radius, *r*_m_ and molecular weight, *M*_w_ for unmodified poly(dT), PEG, amino‐linked poly(dT), and PEGylated poly(dT), as estimated by SEC, is shown in Figure 2. The corresponding elution curves are shown in Figure S2‐S6 in the Supporting Information. The *M*_w_ values, distribution coefficients, *K*, on the SEC, and calculated *r*_m_ values are summarized in Table S3 (B)‐(H) in the Supporting Information.

As shown in Figure 2, PEGylation generally increased *r*_m_ compared to unmodified poly(dT). However, the extent of this increase depends on both the PEG molecular weight and modification site. At an *M*_w_ around 10,000 g·mol^−1^, PEGylated poly(dT) modified at mid‐position with PEG5K and PEG10K exhibited slightly smaller *r*_m_ values than those modified at the 5′‐end.

The fitted curve for poly(dT) (*r*_m_ = 0.030*M*_w_^0.55^, **solid line** in Figure 2) consistently shows a larger molecular radius than PEG (*r*_m_ = 0.019*M*_w_^0.56^ [40], **dashed line** in Figure 2). This suggests that poly(dT), with its linear, negatively charged backbone, adopts a more extended conformation in solution [41] than PEG, forming a more flexible and compact coil.

The slightly larger *r*_m_ of poly(dT) modified at 5′‐end is likely due to electrostatic repulsion at the DNA terminus, which promotes molecular expansion. In contrast, the reduced *r*_m_ observed for PEGylated poly(dT) modified at the mid‐position (M‐PEG5K‐T and M‐PEG10K‐T) suggests a more compact molecular structure. This compaction may result from the steric constraints associated with the branched structure or intramolecular interactions, including hydrogen bonding between the thymine bases [42].

To further examine the origin of the difference in molecular radius between end‐ and mid‐position PEGylated poly(dT), MD simulations were performed for PEGylated 9T with PEG (*M*_w_ = 0.6 kDa), and the interaction patterns between PEG chains and DNA functional groups analyzed (Figure S22 in the Supporting Information). The MD analysis evaluated the mean minimum distance and contact frequency between PEG atoms and individual thymine residues along poly(dT) chain. For the PEGylated 9T modified at the 5′‐end (E‐PEG0.6K‐9T), PEG contacts were predominantly localized near the modification site, mainly involving dT1—dT4 (Figure S22 (A), (B), (E), and (F)). In contrast, for PEGylated 9T modified at the fifth thymine (M‐PEG0.6K‐9T), PEG‐DNA contacts were distributed over a broader range of thymine residues along the 9T chain compared with E‐PEG0.6K‐9T.

These differences in PEG‐DNA contact distribution are consistent with the trend observed in the SEC‐derived molecular radius, where mid‐position PEGylation resulted in slightly smaller rm values than the 5′‐end modification. A more distributed PEG‐DNA contact pattern may facilitate partial chain compaction, leading to a reduced apparent molecular radius. Such compaction behavior is also consistent with previous thermodynamic studies indicating that PEG conjugation does not alter the nominal charge of DNA but can modify local hydration and excluded‐volume effects around nucleobases [23].

### 3.2 Elution Behavior of PEGylated Oligonucleotides in Ion‐Exchange Chromatography

**FIGURE 3:**
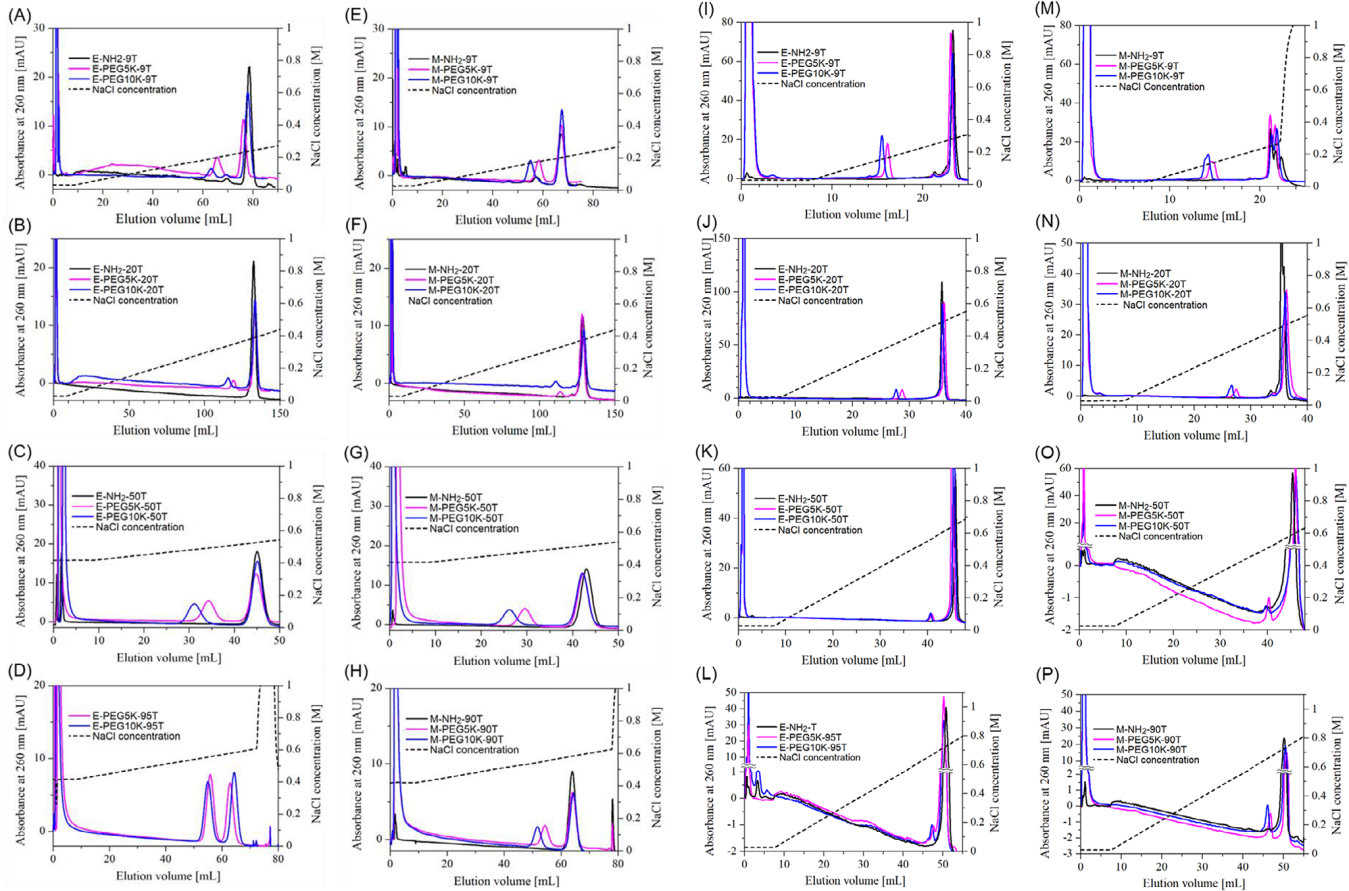
Elution curves under linear salt gradient elution conditions. (A)‐(D) poly(dT) modified at the 5′‐end analyzed using the Q Sepharose HP column (Column A). (E)‐(H) poly(dT) modified at the mid‐position analyzed using the Q Sepharose HP column (Column A). (I)‐(L) poly(dT) modified at the 5′‐end analyzed using the QA monolith column (Column C). (M)‐(P) poly(dT) modified at the mid‐position analyzed using the QA monolith column (Column C). Solid black lines: amino‐linked poly(dT); solid pink lines: PEGylated poly(dT) reaction mixtures with PEG5K; solid blue lines: PEGylated poly(dT) reaction mixtures with PEG10K; dotted lines: NaCl concentration in the column eluent.

Figure 3 shows the elution curves of the amino‐linked poly(dT) (NH_2_‐T) and its reaction mixture with PEG in LGE at a gradient volume of *GH* = 0.002 M using the Q Sepharose HP column (Column A, see Table 2) and the QA monolith (Column C, see Table 2). **Panels (A)**–**(D)** in Figure 3 display the elution curves of PEGylated 9T – 95T reaction mixtures of 5′‐end modification (E‐PEG‐T), while **panels (E)**–**(H)** in Figure 3 show those of PEGylated 9T–90T reaction mixtures of mid‐position modification (M‐PEG‐T). In the LGE, the initial and final salt concentrations for 9T and 20T were set to 30 and 1000 mM, respectively, while for 50T and 95T they were set to 400 and 1000 mM, respectively, owing to their strong electrostatic interactions with the stationary phase.

As shown in Figure 3(A), the PEGylation mixtures of E‐NH_2_‐9T with PEG5K and PEG10K resulted in two distinct peaks: PEGylated 9T with 5′‐end modification (E‐PEG5K‐9T: *I*_R_ = 0.193 M, E‐PEG10K‐9T: *I*_R_ = 0.186 M) and the un‐PEGylated form (E‐NH_2_‐9T), which eluted later at *I*_R_ = 0.235 M. The extent of *I*_R_ reduction depended on both *M*_w_ and the modification site. PEGylated 9T with mid‐position modification (M‐PEG5K‐9T and M‐PEG10K‐9T) eluted even earlier than their 5′‐end counterparts (Figure 3(E)). Similarly, PEGylated 20T, 50T, and 95T eluted earlier than the unPEGylated forms (Figure 3, **panels (B)**–**(D)** and **(F)**–**(H)**). This trend was more pronounced for mid‐position modifications, correlating with the SEC results, in which greater conformational compaction of PEG in mid‐position modifications led to enhanced steric exclusion from the stationary phase in AIEC, resulting in earlier elution.

The elution behavior observed with the QA monolith column was similar to that on the Q Sepharose HP column, with PEGylated poly(dT) eluting earlier than the unPEGylated poly(dT). Regardless of the column type, PEGylated poly(dT) with a higher PEG molecular weight eluted earlier, which is consistent with the steric exclusion from the stationary phase.

The overall trend in the elution salt concentration differences between the unmodified and PEGylated poly(dT) obtained in Figure 3 is summarized in Figure S7 in Supporting Information. The reduction in *I*_R_ was more pronounced on the monolith than on the Q Sepharose HP column. In the following section, the reduction in *I*_R_ is analyzed using a mechanistic model of ion‐exchange chromatography.

### 3.3 Retention Mechanism of PEGylated Oligonucleotide in Ion‐Exchange Chromatography

**FIGURE 4:**
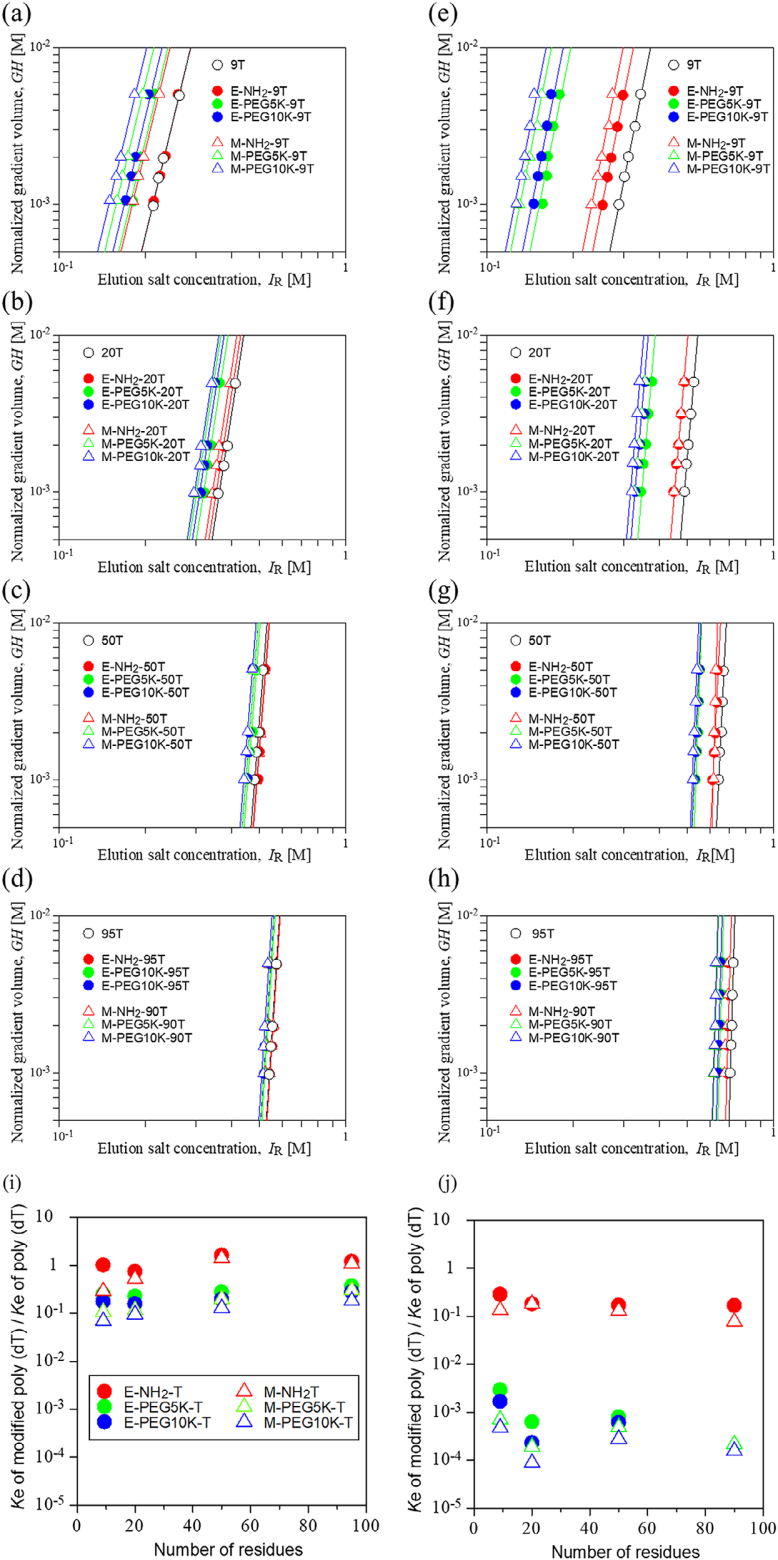
*GH*‐*I*_R_ curves obtained using the Q sepharose HP ((a)–(d)) and the QA monolith column ((e)–(h)) and the relative ion‐exchange equilibrium constant *K*_e_ normalized to unmodified poly (dT) obtained on the Q sepharose HP (i) and QA CIM monolith column (j). Filled circles: amino‐linked poly(dT) at the 5′‐end (E‐NH_2_‐T), PEGylated poly(dT) modified with PEG5K and with PEG10K at the 5′‐end (E‐PEG5K‐T and E‐PEG10K‐T); Unfilled triangles: amino‐linked poly(dT) at a mid‐position thymine (M‐NH2‐T), PEGylated poly(dT) modified at a mid‐position thymine modified with PEG5K and PEG10K (M‐PEG5K‐T and M‐PEG10K‐T).

To further quantify the effect of PEGylation on the retention behavior observed in Section 3.2, the *GH*‐*I*_R_ logarithmic plots were analyzed using a mechanistic ion‐exchange chromatography model. **Panels (a)**–**(h)** in Figure 4 show the logarithmic *GH*‐*I*_R_ plots, where the normalized gradient volume, *GH*, is plotted against the *I*_R_ obtained from LGE experiments using the Q Sepharose HP (Column A) and the QA monolith column (Column C) (see, Figures S8–S16 and Tables S4, S5 in the Supporting Information).

For all unmodified and PEGylated poly(dT), the *GH*‐*I*_R_ plots exhibited linear relationships (Figure 4 (a)–(h)), indicating that the elution behavior can be consistently described by the Yamamoto model [38, 43], which assumes stoichiometric ion‐exchange interactions between the oligonucleotides and the stationary phase. Based on the slopes and intercepts of these plots, the number of binding sites, *B*, and the interaction parameter *A* (= *K*_e_·Λ^B^, where *K*_e_ is the ion exchange equilibrium constant and Λ is the ionic capacity of the carrier) were determined.

Importantly, the slopes of these curves were nearly identical for unmodified poly(dT) and PEGylated poly(dT), regardless of PEG molecular weight or modification site. This indicates that PEGylation does not alter the effective number of binding sites, *B*, suggesting that the fundamental binding stoichiometry between the phosphate groups of poly(dT) and the anion‐exchange ligands remains unchanged upon PEGylation.

As shown in **Panels (i)** and **(j)** of Figure 4, the ratio of the ion‐exchange equilibrium constant *K*_e_ for PEGylated poly(dT) to that for unmodified poly(dT) was essentially independent of the number of nucleotides over the range examined, indicating that the effect of PEGylation on *K*_e_, is independent of the chain length. In contrast, the *K*_e_ ratio depended on both the PEG molecular weight and the modification site. PEGylated poly(dT) with higher PEG molecular weight exhibited slightly lower *K*_e_ values, and this reduction was more pronounced for mid‐position PEG modifications than for 5′‐end modifications. These trends were consistently observed on both the Q Sepharose HP and the QA monolith columns.

Consistent with our previous studies and recent mechanistic analysis [12], the stoichiometric ion‐exchange framework allows the retention behavior of oligonucleotides in AIEC to be interpreted in terms of two distinct parameters. In this framework, the parameter *B* reflects the effective number of binding sites and thus the charge stoichiometry of the oligonucleotide‐resin interaction, whereas the equilibrium constant *K*_e_ reflects the local binding environment that modulates the strength of electrostatic interactions between the oligonucleotide and the stationary phase [12].

As discussed in Section 3.1., MD simulations showed that PEG preferentially associates with nucleobase and sugar moieties rather than directly contacting phosphate groups. Importantly, the simulations also indicated that PEG modification perturbed the local distribution of Na^+^ ions near the DNA backbone without completely excluding them, particularly in the case of mid‐position PEGylation (Figure S22 **(C)**, **(D)**, **(G)** and **(H)** in the Supporting Information), where PEG‐DNA contacts were more spatially distributed along the oligonucleotide chain.

These observations suggest that PEGylation does not alter the effective number of ion‐exchange binding sites but modifies the local electrostatic environment surrounding phosphate groups, resulting in a reduction of *K*_e._ This PEG‐induced modulation of the binding environment provides a molecular‐level explanation for the charge‐shielding effect observed in the chromatographic retention behavior.

### 3.4 Mass‐Transfer Behavior of PEGylated Oligonucleotides in Ion‐Exchange Chromatography

**FIGURE 5:**
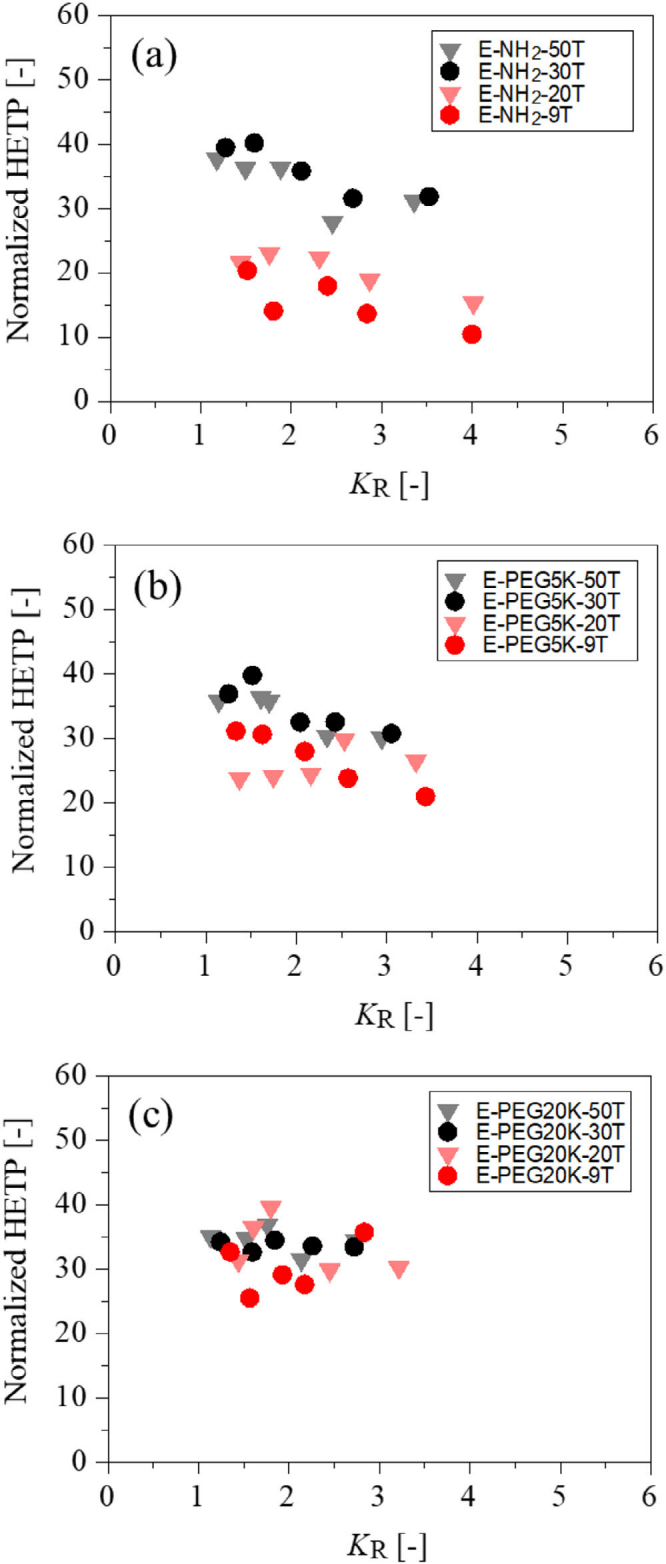
Normalized height equivalent to a theoretical plate (HETP) as a function of the distribution coefficient *K*_R_ for PEGylated poly(dT) on the Q Sepharose HP column. HETP values were normalized by the particle diameter. (a) Amino‐linked poly(dT) at the 5′‐end (E‐NH2‐T). (b) PEGylated poly(dT) at the 5′‐end with PEG5 kDa (E‐PEG5K‐T). (c) PEGylated poly(dT) at the 5′‐end with PEG 20 kDa (E‐PEG20K‐T).

As PEGylation increased the molecular radius of poly(dT), as observed by size‐exclusion chromatography in Figure 2, PEGylated species were eluted at lower salt concentrations by ion‐exchange chromatography (Figures 3, 4). Analysis of peak broadening under these linear gradient elution conditions on porous AIEC (see, Figure S17 in the Supporting Information) showed that the dependence of HETP on retention weakened with increasing PEG molecular weight (Figure 5), as evidenced by the reduced variation of HETP with *K*_R_ across different chain lengths (see also, Figure S18‐S20, and Table S6 in the Supporting Information). Similar retention‐dependent mass‐transfer behavior has been reported for unmodified oligonucleotides and attributed to interparticle diffusion limitations in porous resins [18, 19, 20, 21].

However, because PEGylation simultaneously increased molecular size and modified the local electrostatic environment, the observed changes in HETP cannot be attributed solely to restricted pore diffusion. Instead, the results indicate that PEGylation reduced retention‐dependent mass‐transfer effects through a combined contribution of steric hindrance and modulation of electrostatic interactions near the resin surface, consistent with previous observations reported for PEGylated protein in ion‐exchange chromatography [44].

## 4 Conclusions

In this study, the effect of PEGylation on the retention and mass‐transfer behavior of poly(dT) were systematically investigated using both a multi‐porous resin‐packed column and a monolithic column under linear gradient elution. PEGylation consistently reduced the elution salt concentrations on both columns, however, the effective number of ion‐exchange binding sites, *B*, remained unchanged regardless of the PEG modification site or molecular weight.

In contrast, the ion‐exchange equilibrium constant, *K*_e_, was found to decrease upon PEGylation, with its magnitude depending on the PEG structure, modification site, and type of stationary phase. Size‐exclusion chromatography confirmed that PEGylation increased the apparent molecular radius of the oligonucleotide. While SEC primarily reflects steric size‐exclusion and does not directly prove electrostatic interactions, the observed reduction in *K*_e_ cannot be explained by size effects alone.

Molecular dynamic simulations provided complementary molecular‐level insight, indicating that PEG preferentially associates with nucleobase and sugar moieties rather than directly contacting phosphate groups. These results suggest that PEGylation does not alter the intrinsic charge structure of DNA but modulates the local interaction environment surrounding the phosphate groups. Such modulation is consistent with a charge‐shielding effect in which the accessibility of counterions and ion‐exchange ligands to the phosphate groups is partially hindered by the presence of PEG, leading to a reduction in *K*_e_.

The preservation of *B* values after PEGylation implies that the elution salt concentration of PEGylated oligonucleotides can be predicted from that of their unmodified counterparts. This finding is particularly useful in the early stages of oligonucleotide drug development, where PEGylated materials are often limited due to low PEGylation reaction yields and the scarcity of the starting material.

Despite the increased molecular size caused by PEGylation, the HETP values for PEGylated poly(dT) did not significantly increase in the Q Sepharose HP column. The weakened dependence of HETP on retention for PEGylated poly(dT) suggests that PEGylation modifies intraparticle mass‐transfer behavior without inducing severe band broadening. Taken together, these results demonstrate that PEGylated poly(dT) can be efficiently separated by AIEC without compromising separation performance.

From a practical perspective, while binding capacity remains an important consideration, multi‐porous resin‐packed columns are expected to provide practical advantages in terms of robustness and scalability for the preparative separation of PEGylated oligonucleotides.

## Sample Abbreviations

 

E‐NH_2_ series: DNAs modified with a C6‐amino linker at the 5′ ‐phosphate group
M‐NH2 series: DNAs modified with a C6‐amino linker at a mid‐position thymine base.

## Author Contributions

Noriko Yoshimoto designed and supervised the research and performed MD simulations. Yoshiatsu Ono conducted the chromatographic experiment and analyzed the retention behavior of PEGylated DNA using Q Sephaorose HP (Column A) and QA monolith column (Column C). Tomoya Matsumoto performed the experiments and data analysis of the mass‐transfer behavior of PEGylated DNA using Q sepharose HP (Column C). Yuma Kumagai partially contributed the experiments and data analysis of the mass‐transfer behavior of PEGylated DNA using Q sepharose HP (Column C). All authors have read and approved the final manuscript.

## Funding

This work was supported by the Japan Society for the Promotion of Science (JSPS) KAKENHI Grant Number 18K04854, and partly by 25K08403.

## Conflicts of Interest

The authors declare no conflict of interest.

## Declaration of Generative AI in Scientific Writing

The authors state that AI‐assisted technology was only used in the writing process to improve the readability and language of the manuscript.

## Supporting information




**Supporting File**: biot70183‐sup‐0001‐SuppMat.pdf.

## Data Availability

The data supporting the finding of this study are available from the corresponding author upon reasonable request.
