# Supplementary material for: Mechanistic Insights Into the Retention and Separation Mechanism of Poly(Ethylene Glycol)‐Modified Short Oligonucleotides in Anion‐Exchange Chromatography
Source: Biotechnol J. 2026 Jan 26;21(1):e70183. doi: 10.1002/biot.70183 (PMC12835577; doi:10.1002/biot.70183)
Supplement: Supplementary file 1 — Supporting File: biot70183‐sup‐0001‐SuppMat.pdf. [file BIOT-21-e70183-s001.pdf]

Supporting Information

Retention and Separation Mechanism of Poly(ethylene glycol)-Modified Short Oligonucleotides in Anion-Exchange Chromatography

Noriko Yoshimoto*[1], Tomoya Matsumoto[1], Yoshiatsu Ono[1], Yuma Kumagaya[1]

[1]Department of Applied Chemistry, Yamaguchi University, Tokiwadai 2-16-1 Ube 755-8611, Japan

*Corresponding author: e-mail: noriko-y@yamaguchi-u.ac.jp

Contents

1. PEGylation reaction conditions for oligonucleotide. p.S2

Table S1, **S2**

2. Molecular radius of PEGylated oligonucleotide. p.S4

Figure S1-S6, **Table S3**

3. Elusion salt concentrations in a linear salt gradient elution experiment. p.S11

Figure S7

4. Data from linear salt gradient elution experiments using Q Sepharose HP column. p.S12

Figure S8-S11, **Table S4**

5. Data from linear salt gradient elution experiments using QA monolith column. p.S29

Figure S12-S15, **Table S5**

6. Calculated elution salt concentration of PEGylation mixtures in linear salt gradient elution. p.S46

Figure S16

7. Data used to determine mass transfer properties of PEGylated oligonucleotide. p.S48

Figure S17-S20, Table S6

8. MD simulations for local interaction tendencies between PEG chains and DNA functional groups. p.S61

Figure S21, 22

1. PEGylation reaction conditions of oligonucleotide.

In the PEGylation reaction of amino-linked poly(dT) with activated PEG, the concentrations were set at lower for smaller column (column A, prepacked Q Sepharose HP Column and column C, QA monolith column, see **Table2**) than those for larger column (column B, self-packed Q Sepharose HP column, see **Table 2**) than those

For investigation of hydrodynamic radius using TSK G3000PW$_{XL}$ column and the retention behavior in linear salt gradient experiments (LGE) using smaller column A and column C, poly(dT) purchased from Tsukuba Oligo service (Tsukuba, Japan) was used. The stock solution was prepared with pure water. The concentration of each poly(dT) and PEG was set as listed in **Table S1**. The sample for LGE was directly withdrawn from the reaction mixture.

For investigation of mas transfer using HETP measurements with larger column B, poly(dT) purchased from FASMAC (Kanagawa, Japan). The stock solution was prepared with 10 mM sodium phosphate buffer containing 30 mM NaCl (pH7). The concentration of each poly(dT) in PEGylation was set as listed in T**able S2**. After PEGylation, the reaction mixture was diluted at a given concentration with 10 mM Tris-HCl containing 30 mM NaCl (pH7).

Table S1. Concentration in SEC experiments using a TSK G3000PW$_{XL}$ column and PEGylation reaction conditions for linear salt gradient experiments with column A (Q Sepharose HP) and column C (CIM QA monolith).

**Sample**	**Stock solution**	**PEGylation reaction and LGE**		
**name**[a]	**[NH$_2$-T]**[b] **[M]**	**[NH$_2$-T]**[c] **[M]**	**[PEG]**[d] **[M]**	**[PEG]/[NH$_2$-T]**[e]
**amino-linked poly(dT) modified at the 5'-end (E-NH$_2$ series)**				
E-NH$_2$-9T	1.81×10^{-7}	6.03×10^{-9}	6.03×10^{-4}	100000
E-NH$_2$-20T	8.70×10^{-8}	2.90×10^{-9}	2.90×10^{-4}	100000
E-NH$_2$-50T	3.48×10^{-8}	1.16×10^{-9}	1.16×10^{-4}, 4.64×10^{-4}	100000, 400000
E-NH$_2$-95T	1.14×10^{-8}	3.80×10^{-10}	3.80×10^{-5}, 1.52×10^{-5}	100000, 400000
**amino-linked poly(dT) modified at a mid-position thymine (M-NH$_2$ series)**				
M-NH$_2$-9T	1.93×10^{-7}	6.43×10^{-9}	6.43×10^{-4}	100000
M-NH$_2$-20T	8.61×10^{8}	2.87×10^{-9}	2.87×10^{-4}	100000
M-NH$_2$-50T	3.47×10^{-8}	1.16×10^{-9}	1.16×10^{-4}, 4.64×10^{-4}	100000, 400000
M-NH$_2$-90T	9.64×10^{-9}	3.21×10^{-10}	3.21×10^{-5}, 1.28×10^{-4}	100000, 400000

[a]Sample name: E-NH$_2$ and M-NH$_2$ denote amino-linked poly(dT) modified at the 5'-end and at a mid-position thymine, respectively. The base length of each sample and the modification sites are described in **Table 1**.

[b][NH$_2$-T]: Concentration of amino-linked poly(dT) in the stock solution.

[c][NH$_2$-T]: Concentration of amino-linked poly(dT) in the PEGylation reaction.

[d][PEG]: Concentration of NHS-activated PEG5K or PEG10K in the PEGylation reaction.

[e][PEG]/[NH$_2$-T]: Molar ration of PEG to amino-linked poly(dT) in the PEGylation reaction.

Table S2. Concentrations of amino-linked or PEGylated poly(dT) modified at the 5′-end in the PEGylation reaction or HETP sample using column B (Q Sepharose HP).

**Sample name**[a]	**Stock solution**	**PEGylation reaction**			**HETP measurement**
	**$[E\text{-}NH_2\text{-}T]^b$ [M]**	**$[E\text{-}NH_2\text{-}T]^c$ [M]**	**$[PEG]^d$ [M]**	**$[PEG]/[E\text{-}NH_2\text{-}T]^e$**	**$[E\text{-}NH_2\text{-}T] + [E\text{-}PEG\text{-}T]^f$ [M]**
$E\text{-}NH_2\text{-}9T$	6.41×10^{-8},<br>7.25×10^{-8}	6.41×10^{-8},<br>7.25×10^{-8}	3.20×10^{-6},<br>3.63×10^{-6}	50	6.04×10^{-9}
$E\text{-}NH_2\text{-}20T$	5.98×10^{-8}<br>8.70×10^{-8}	5.98×10^{-8}<br>8.70×10^{-8}	2.99×10^{-6}<br>4.35×10^{-6}	50	1.09×10^{-9}
$E\text{-}NH_2\text{-}50T$	2.17×10^{-8}	2.17×10^{-8}	1.09×10^{-6}	50	3.61×10^{-9}
$E\text{-}NH_2\text{-}95T$	3.48×10^{-8}	3.48×10^{-8}	1.74×10^{-6}	50	2.18×10^{-9}

[a]Sample name: $E\text{-}NH_2$ denote amino-linked poly(dT) modified at the 5′-end. See **Table 1** for base length and modification site of each sample.

[b]$[E\text{-}NH_2\text{-}T]$: Concentration of amino-linked poly(dT) in the stock solution.

[c]$[E\text{-}NH_2\text{-}T]$: Concentration of amino-linked poly(dT) in the PEGylation reaction.

[d][PEG]: Concentration of NHS-activated PEG5K or PEG10K in the PEGylation reaction.

[e]$[PEG]/[NH_2\text{-}T]$: Molar ration of PEG to amino-linked poly(dT) in the PEGylation reaction.

[f]$[E\text{-}NH_2\text{-}T] + [E\text{-}PEG\text{-}T]$: Total Concentration of unreacted and PEGylated poly(dT).

2. Molecular radius of PEGylated oligonucleotide.

The molecular radius of PEGylated poly(dT) was measured using size exclusion chromatography with a TSKgel G3000PWXL column. In this study, we employed an activated PEG with N-hydroxysuccinimide (NHS-PEG), as a standard sample. The absorption of the column elution at 215 nm as shown in **Figure S1 (A)**. A 10 mM Tris-HCl buffer containing 30 mM NaCl (pH7) was used as the mobile phase. The flow rate and injection sample volume were set to 0.1 mL/min and 100 μL, respectively. As shown in **Table S3(A)**, the molecular radius (r_m) of PEG was calculated using the equation relating the molecular radius of PEG to its molecular weight [S1].

$$r_m = 0.019M_r^{0.559} \qquad \text{(S1)}$$

As shown in **Figure S1 (B)**, the calibration curve of the column was generated based on the relationship between the distribution coefficient (K) and r_m using data obtained in this study and reference data from the manufacture (Tosoh Bioscience [S1]) :

$$r_m = 10.6e^{-7.55\,K} \qquad \text{(S2)}$$

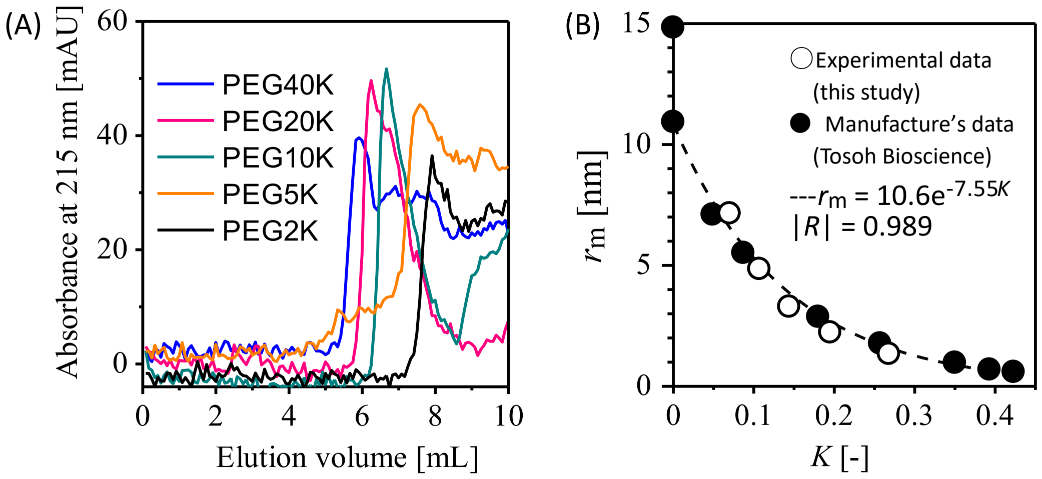

Figure S1. Elution curves on a TSKgel G3000PW$_{XL}$ column (A) and calibration curve of the molecular radius for the TSKgel G3000PW$_{XL}$ column based on the distribution coefficient using PEG (B).
open circle: Experimental data (this study), filled circle: Manufacture's data (Tosoh Bioscience [S2])

For unmodified and PEGylated poly (dT), a 10 mM Tris-HCl buffer containing 150 mM NaCl (pH7) was used as the mobile phase. The flow rate and injection sample volume were set to 0.1 mL/min and 100 μL, respectively. Column elution was monitored at 260 nm.

(A) poly(dT)

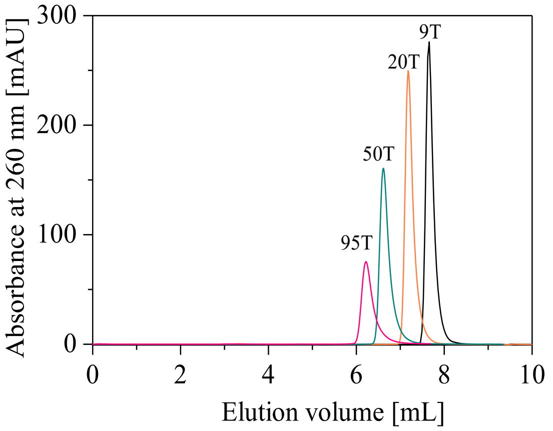

(B) amino-linked poly(dT) at the 5'-end

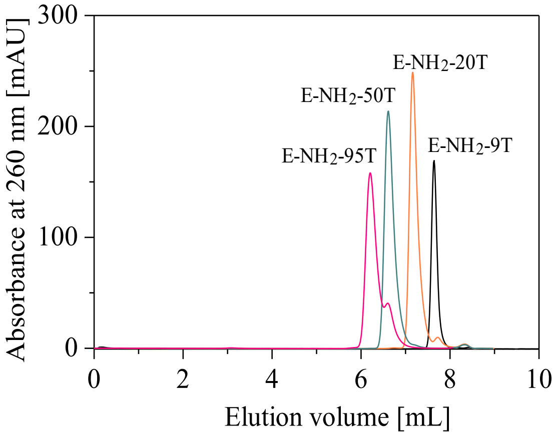

(C) amino-linked poly(dT) at the mid-thymine

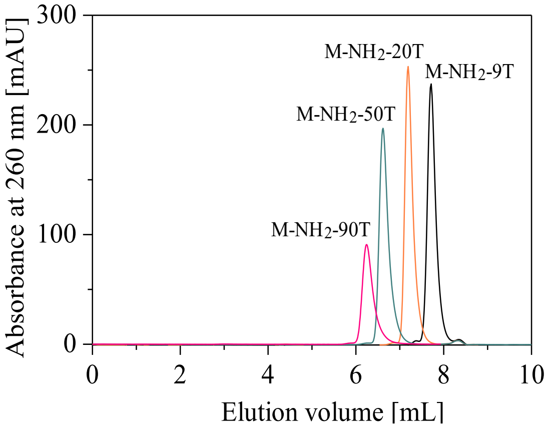

Figure S2. Elution curves of poly(dT) on a G3000PW$_{XL}$ column.

(A) unmodified poly(dT)

(B) amino-linked poly(dT) at the 5' -end (E-NH_2-T)

(C) amino-linked poly(dT) at a mid-position thymine (M-NH_2-T)

The distribution coefficients (*K*) of poly(dT) are summarized in **Table S3 (B-D)** (poly(dT), E-NH_2-T, M-NH_2-T series). The injection volume was 100 μL, and the sample concentration are shown in **Table S1**. The mobile phase consisted of 10 mM Tris-HCl buffer with 150 mM NaCl (pH7), with a flow rate of 0.1 mL/min.

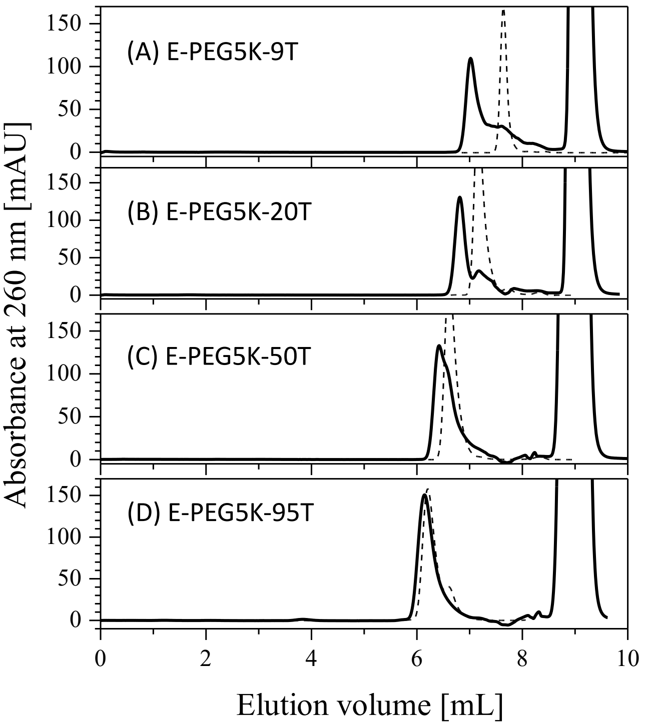

Figure S3. Elution curves of the PEGylation reaction mixture of 5'-end amino-linked poly(dT) and NHS-activated PEG5K on a G3000PW$_{XL}$ column.

(A) Solid line: reaction mixture of amino-linked 9T at the 5'-end (E-PEG5K-9T) and PEG5K; dashed line: amino-linked 9T at the 5' -end (E-NH_2-9T)

(B) Solid line: reaction mixture of amino-linked 20T at the 5'-end (E-PEG5K-20T) and PEG5K; dashed line: amino-linked 20T at the 5' -end (E-NH_2-20T)

(C) Solid line: reaction mixture of amino-linked 50T at the 5'-end (E-PEG5K-50T) and PEG5K; dashed line: amino-linked 50T at the 5' -end (E-NH_2-50T)

(D) Solid line: reaction mixture of amino-linked 95T at the 5'-end (E-PEG5K-95T) and PEG5K; dashed line: amino-linked 95T at the 5' -end (E-NH_2-95T)

The distribution coefficients (*K*) of PEGylated poly(dT) with PEG5K at the 5' -end (E-PEG5K-T), which eluted first in the curve, followed by amino-linked poly(dT) and hydrolyzed NHS, are summarized in **Table S3 (E)** (E-PEG5K-T series).

The injection volume was 100 μL, and the sample concentration are shown in **Table S1**). The mobile phase consisted of 10 mM Tris-HCl buffer with 150 mM NaCl (pH7), with a flow rate of 0.1 mL/min.

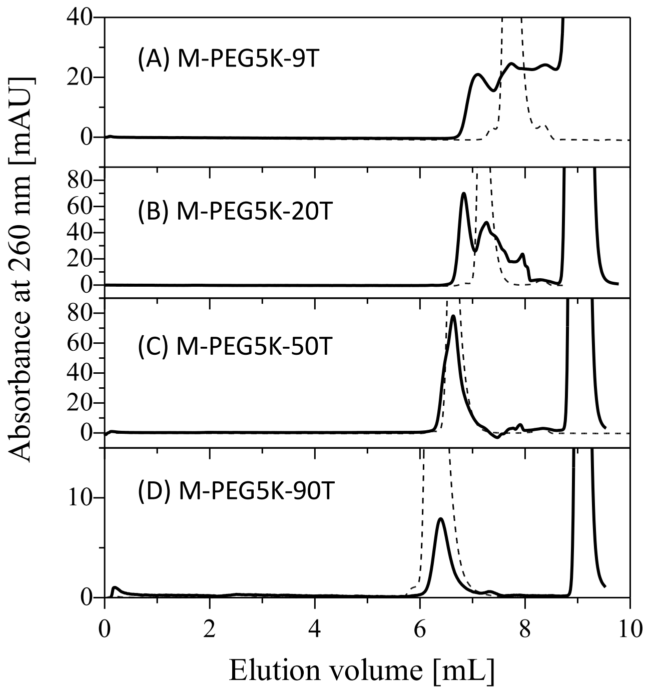

Figure S4. Elution curves of the PEGylation reaction mixture of mid-position amino-linked poly(dT) and PEG5K on a G3000PW$_{XL}$ column.

(A) Solid line: reaction mixture of amino-linked 9T at the 5th thymine (M-PEG5K-9T) and PEG5K; dashed line: amino-linked 9T at the 5th thymine (M-NH$_2$-9T)

(B) Solid line: reaction mixture of amino-linked 20T at the 10th thymine (M-PEG5K-20T) and PEG5K; dashed line: amino-linked 20T at the 10th thymine (M-NH$_2$-20T)

(C) Solid line: reaction mixture of amino-linked 50T at the 24th thymine (M-PEG5K-50T) and PEG5K; dashed line: amino-linked 50T at the 24th thymine (M-NH$_2$-50T)

(D) Solid line: reaction mixture of amino-linked 90T at the 45th thymine (M-PEG5K-90T) and PEG5K; dashed line: amino-linked 90T at the 45th thymine (M-NH$_2$-90T)

The distribution coefficients (*K*) of PEGylated poly(dT) with PEG5K at a mid-position thymine (M-PEG5K-T), which eluted first in the curve, followed by amino-linked poly(dT) and hydrolyzed NHS, are summarized in **Table S3 (F)** (M-PEG5K-T series).

The injection volume was 100 μ, and the sample concentration are shown in **Table S1**. The mobile phase consisted of 10 mM Tris-HCl buffer with 150 mM NaCl (pH7), with a flow rate of 0.1 mL/min.

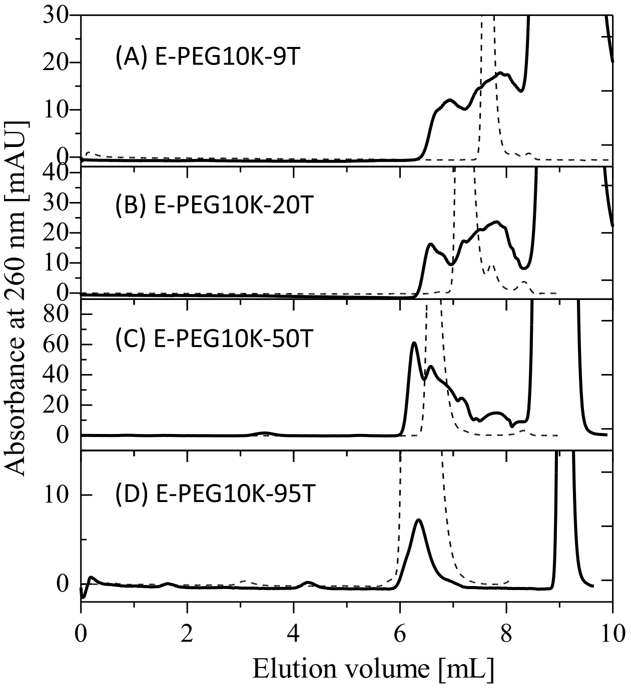

Figure S5. Elution curves of the PEGylation reaction mixture of 5′ -end amino-linked poly(dT) and NHS-activated PEG10K on a G3000PW$_{XL}$ column.

(A) Solid line: reaction mixture of amino-linked 9T at the 5′-end (E-PEG10K-9T) and PEG10K; dashed line: amino-linked 9T at the 5′ -end (E-NH_2-9T)

(B) Solid line: reaction mixture of amino-linked 20T at the 5′-end (E-PEG10K-20T) and PEG10K; dashed line: amino-linked 20T at the 5′ -end (E-NH_2-20T)

(C) Solid line: reaction mixture of amino-linked 50T at the 5′-end (E-PEG10K-50T) and PEG10K; dashed line: amino-linked 50T at the 5′ -end (E-NH_2-50T)

(D) Solid line: reaction mixture of amino-linked 95T at the 5′-end (E-PEG10K-95T) and PEG10K; dashed line: amino-linked 95T at the 5′ -end (E-NH_2-95T)

The distribution coefficients (*K*) of PEGylated poly(dT) with PEG10K at the 5′ -end (E-PEG10K-T), which eluted first in the curve, followed by amino-linked poly(dT) and hydrolyzed NHS, are summarized in **Table S3 (G)** (E-PEG10K-T series).

The injection volume was 100 μL, and the sample concentration are shown in **Table S1**. The mobile phase consisted of 10 mM Tris-HCl buffer with 150 mM NaCl (pH7), with a flow rate of 0.1 mL/min.

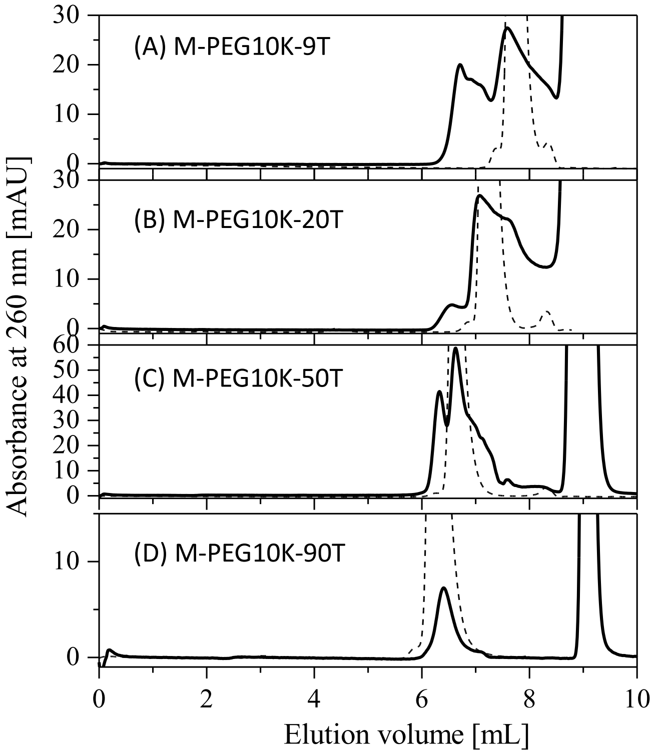

Figure S6. Elution curves of the PEGylation reaction mixture of mid-position amino-linked poly(dT) and NHS-activated PEG10K on a G3000PW$_{XL}$ column.

(A) Solid line: reaction mixture of amino-linked 9T at the 5th thymine (M-PEG10K-9T) and PEG10K; dashed line: amino-linked 9T at the 5th thymine (M-NH_2-9T)

(B) Solid line: reaction mixture of amino-linked 20T at the 10th thymine (M-PEG10K-20T) and PEG10K; dashed line: amino-linked 20T at the 10th thymine (M-NH_2-20T)

(C) Solid line: reaction mixture of amino-linked 50T at the 24th thymine (M-PEG10K-50T) and PEG10K; dashed line: amino-linked 50T at the 24th thymine (M-NH_2-50T)

(D) Solid line: reaction mixture of amino-linked 95T at the 45th thymine (M-PEG10K-90T) and PEG10K; dashed line: amino-linked 90T at the 45th thymine (M-NH_2-90T)

The distribution coefficients (*K*) of PEGylated poly(dT) with PEG10K at a mid-position thymine (M-PEG10K-T), which eluted first in the curve, followed by amino-linked poly(dT) and hydrolyzed NHS, are summarized in **Table S3 (H)** (M-PEG10K-T series).

The injection volume was 100 μL, and the sample concentration are shown in **Table S1**. The mobile phase consisted of 10 mM Tris-HCl buffer with 150 mM NaCl (pH7), with a flow rate of 0.1 mL/min.

Table S3. Molecular weight and radius of oligonucleotides determined using size exclusion chromatography.

Sample name	Molecular weight [g/mol]	Distribution coefficient, *K*	Hydration radius, r_m [nm]
**(A) PEG**			
PEG2K	2000	0.268	1.3
PEG5K	5000	0.195	2.2
PEG10K	10000	0.144	3.3
PEG20K	20000	0.107	4.9
PEG40K	40000	0.055	7.2
**(B) Poly(dT) (poly(dT) series)**			
9T	2676	0.2	2.3
20T	6022	0.145	3.5
50T	15148	0.078	5.9
95T	28337	0.032	8.3
**(C) amino-linked poly(dT) modified at the 5' -end (E-NH_2-T series)**			
E-NH_2-9T	2776	0.197	2.4
E-NH_2-20T	6122	0.142	3.6
E-NH_2-50T	15248	0.085	5.6
E-NH_2-95T	27421	0.033	8.3
**(D) amino-linked poly(dT) modified at a mid-position thymine (M-NH_2-T series)**			
M-NH_2-9T	2791	0.207	2.2
M-NH_2-20T	6137	0.153	3.3
M-NH_2-50T	15263	0.079	5.8
M-NH_2-90T	27436	0.041	7.8
**(E) PEGylated poly(dT) modified at the 5' -end with PEG5K (E-PEG5K-T series)**			
E-PEG5K-9T	7776	0.133	3.9
E-PEG5K-20T	11122	0.100	5.0
E-PEG5K-50T	20248	0.055	7.0
E-PEG5K-95T	32421	0.021	9.0
**(F) PEGylated poly(dT) modified at a mid-position thymine with PEG5K (M-PEG5K-T series)**			
M-PEG5K-9T	7791	0.194	2.6
M-PEG5K-20T	11137	0.161	3.4
M-PEG5K-50T	20263	0.144	4.0
M-PEG5K-90T	32436	N/D[a]	N/D[a]
**(G) PEGylated poly(dT) modified at the 5' -end with PEG10K (E-PEG10K-T series)**			
E-PEG10K-9T	12776	0.114	4.5
E-PEG10K-20T	16122	0.077	5.9
E-PEG10K-50T	25248	0.045	7.5
E-PEG10K-95T	38437	N/D[a]	N/D[a]
**(H) PEGylated poly(dT) modified at a mid-position thymine with PEG10K (M-PEG10K-T series)**			
M-PEG10K-9T	12791	0.114	4.5
M-PEG10K-20T	16137	0.080	5.8
M-PEG10K-50T	25263	0.044	7.6
M-PEG10K-90T	37436	N/D[a]	N/D[a]

[a]The peak corresponding to PEGylated poly(dT) was difficult to distinguish.

3. Elusion salt concentrations in linear salt gradient elution experiments.

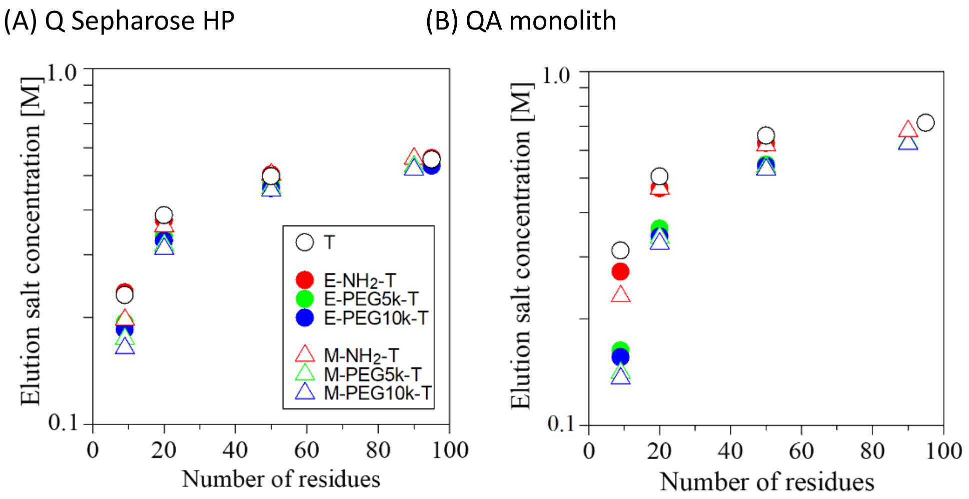

Figure S7. Elution salt concentration of amino-linked or PEGylated poly(dT) on Q Sepharose HP (A) and QA monolith column (B) in a linear salt gradient elution experiment.

Elution salt concentrations were measured at *GH* = 0.002 M. Unfilled circles: unmodified poly(dT) (T); filled circles: amino-linked poly(dT) (E-NH_2-T) and PEG-poly(dT) modified at the 5' -end (E-PEG5K-T, E-PRG10K-T); unfilled triangles: amino-linked poly(dT) (M-NH_2-T) and PEG-poly(dT) modified at mid-position thymine (M-PEG5K-T, M-PRG10K-T).

4. Data from linear salt gradient elution experiments using Q Sepharose HP column.

(A) Elution curves of 9T.

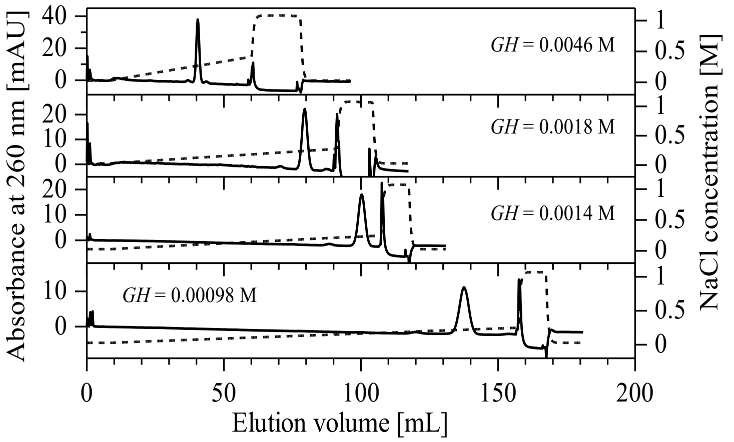

(B) Elution curves of amino-linked 9T modified at the 5′ -end (E-NH_2-9T).

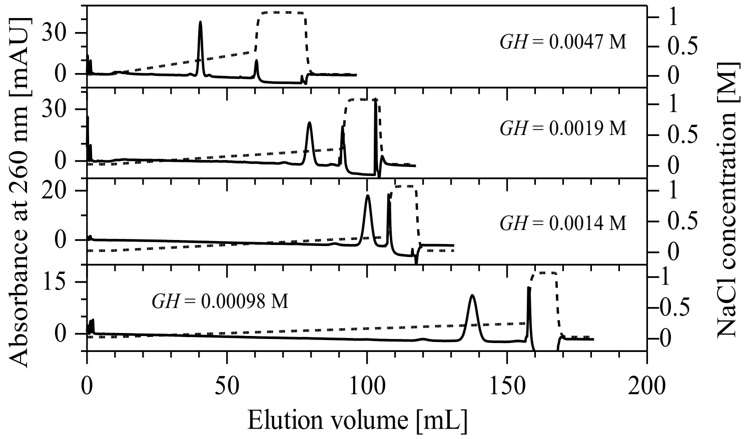

(C) Elution curves of a mixture of PEGylated 9T modified with PEG5K at the 5′ -end (E-PEG5K-9T) and amino-linked 9T modified at the 5′ -end (E-NH_2-9T).

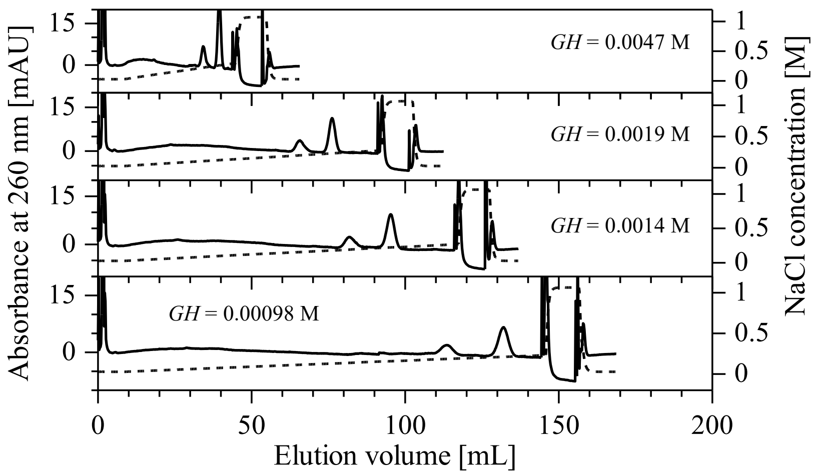

Figure S8 *continued*

(D) Elution curves of a mixture of PEGylated 9T modified with PEG10K at the 5′ -end (E-PEG10K-9T) and amino-linked 9T modified at the 5′ -end (E-NH_2-9T).

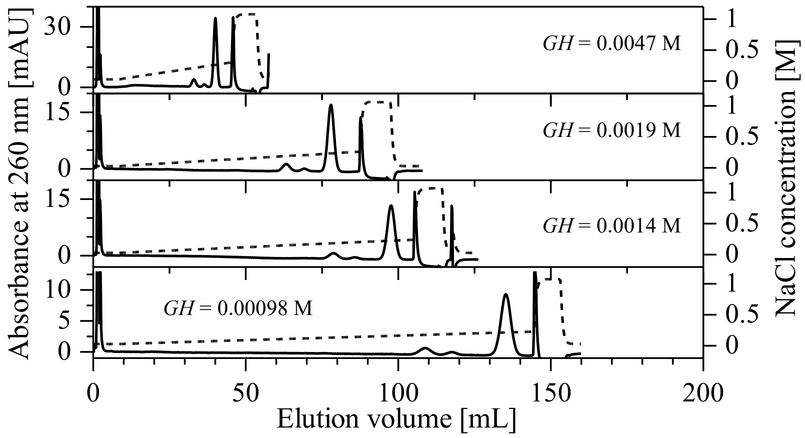

(E) Elution curves of amino-linked 9T modified at the 5th thymine (M-NH_2-9T)

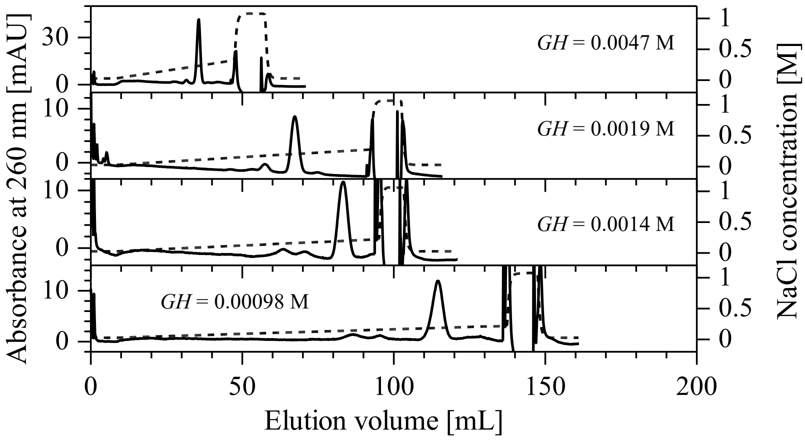

(F) Elution curves of a mixture of PEGylated 9T modified with PEG5K at the 5th thymine (M-PEG5K-9T) and amino-linked 9T modified at the 5th thymine (M-NH_2-9T)

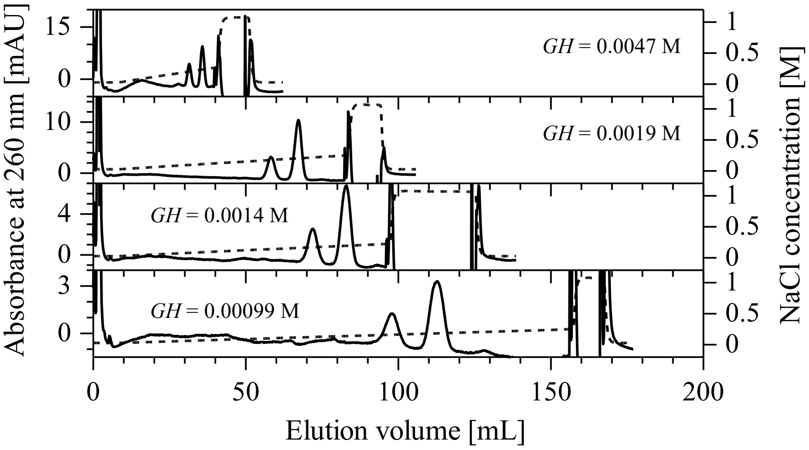

Figure S8 *continued*

(G) Elution curves of a mixture of PEGylated 9T modified with PEG5K at the 5th thymine (M-PEG5K-9T) and amino-linked 9T modified at the 5th thymine (M-NH_2-9T)

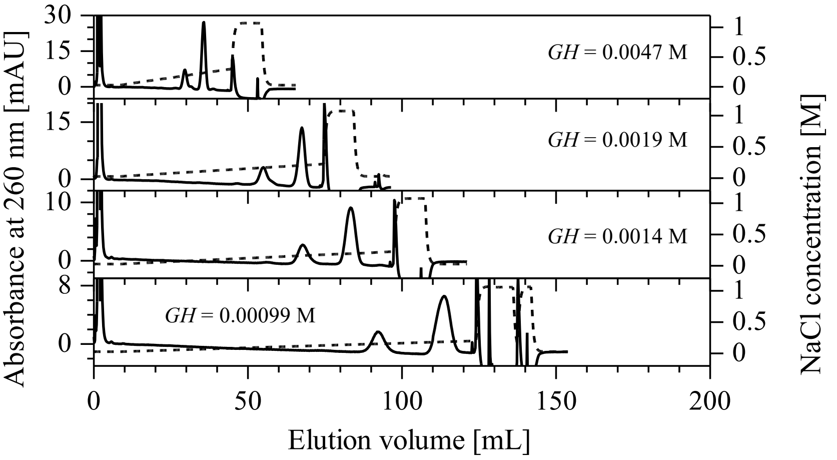

Figure S8. Elution curves of unmodified and modified 9T in linear salt gradient elution using a Q Sepharose HP column.

(A) 9T

(B) amino-linked 9T modified at the 5′ -end (E-NH_2-9T)

(C) a mixture of PEGylated 9T modified with PEG5K at the 5′ -end (E-PEG5K-9T) and amino-linked 9T modified at the 5′ -end (E-NH_2-9T)

(D) a mixture of PEGylated 9T modified with PEG10K at the 5′ -end (E-PEG10K-9T) and amino-linked 9T modified at the 5′ -end (E-NH_2-9T)

(E) amino-linked 9T modified at the 5th thymine (M-NH_2-9T)

(F) a mixture of PEGylated 9T modified with PEG5K at the 5th thymine (M-PEG5K-9T) and amino-linked 9T modified at the 5th thymine (M-NH_2-9T)

(G) a mixture of PEGylated 9T modified with PEG10K at the 5th thymine (M-PEG10K-9T) and amino-linked 9T modified at the 5th thymine (M-NH_2-9T)

The solid and dotted lines represent the absorbance at 260 nm of the column elution and the elution salt concentration, respectively. The salt concentration of the mobile phase was linearly varied from 30 mM to 1 M using a low-salt buffer (10 mM sodium phosphate buffer solution containing 30 mM NaCl, pH 7; buffer A) and a high-salt buffer (10 mM sodium phosphate buffer solution containing 1 M NaCl, pH7; buffer B). The gradient volume of the salt concentration (V_g), initial and final salt concentration (I_0 and I_F), *GH*, and peak elution salt concentration (I_R) are summarized in **Table S4**.

Column A (see **Table 2**) was used. The sample injection volume was 100 μL. The sample concentrations are shown in **Table S1**. The flow rate was set to 1.0 mL/min.

(A) Elution curves of 20T

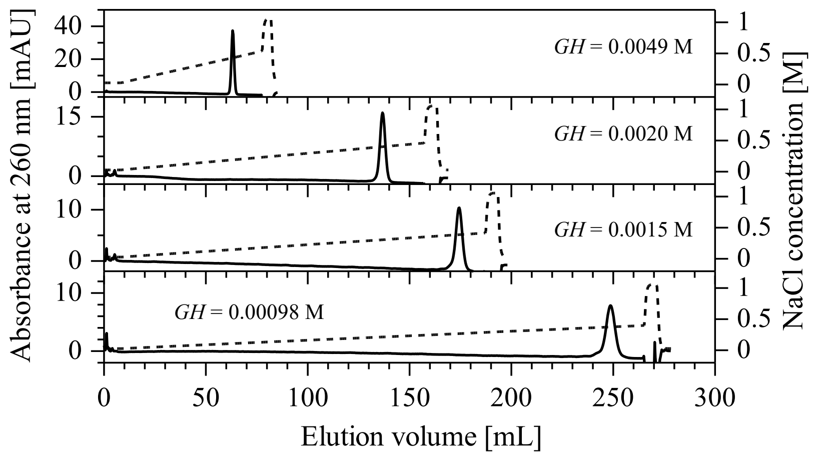

(B) Elution curves of amino-linked 20T modified at the 5′ -end (E-NH_2-20T)

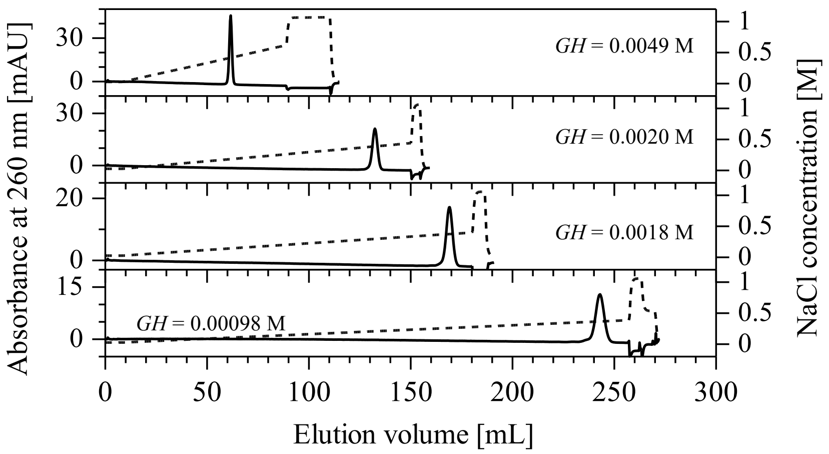

(C) Elution curves of a mixture of PEGylated 20T modified with PEG5K at the 5′ -end (E-PEG5K-20T) and amino-linked 20T modified at the 5′ -end (E-NH_2-20T)

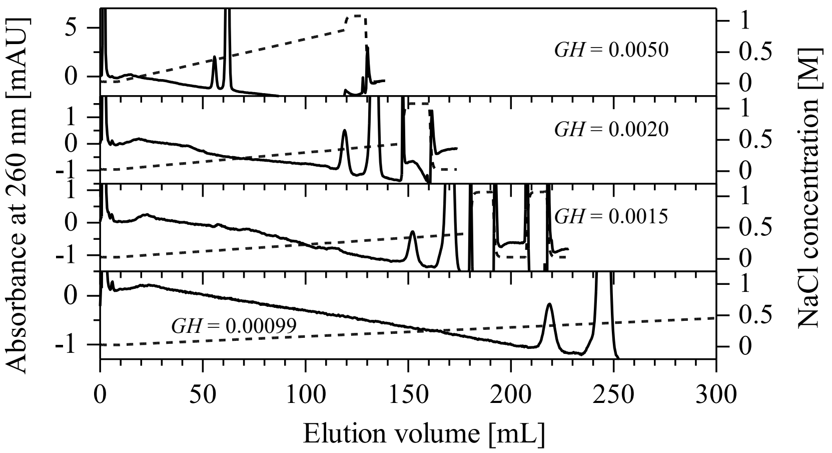

Figure S9 *continued*

(D) Elution curves of a mixture of PEGylated 20T modified with PEG10K at the 5′ -end (E-PEG10K-20T) and amino-linked 20T modified at the 5′ -end (E-NH_2-20T)

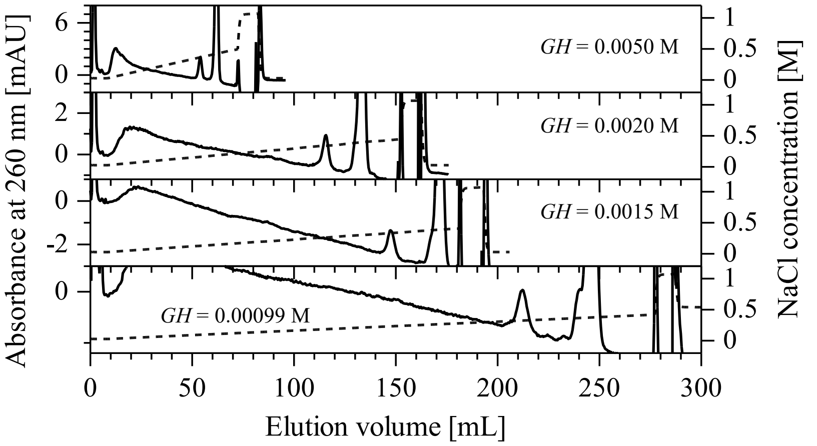

(E) Elution curves of amino-linked 20T modified at the 10th thymine (M-NH_2-20T)

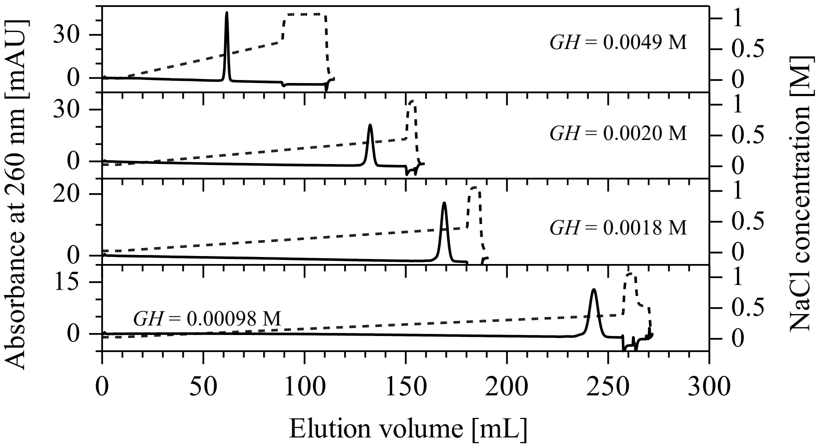

(F) Elution curves of a mixture of PEGylated 20T modified with PEG5K at the 10th thymine (M-PEG5K-20T) and amino-linked 20T modified at the 10th thymine (M-NH_2-20T)

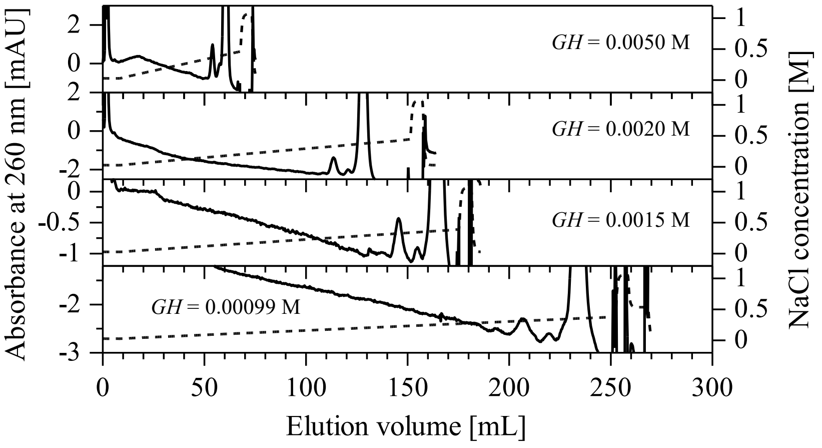

Figure S9 *continued*

(G) Elution curves of a mixture of PEGylated 20T modified with PEG10K at the 10th thymine (M-PEG10K-20T) and amino-linked 20T modified at the 10th thymine (M-NH_2-20T)

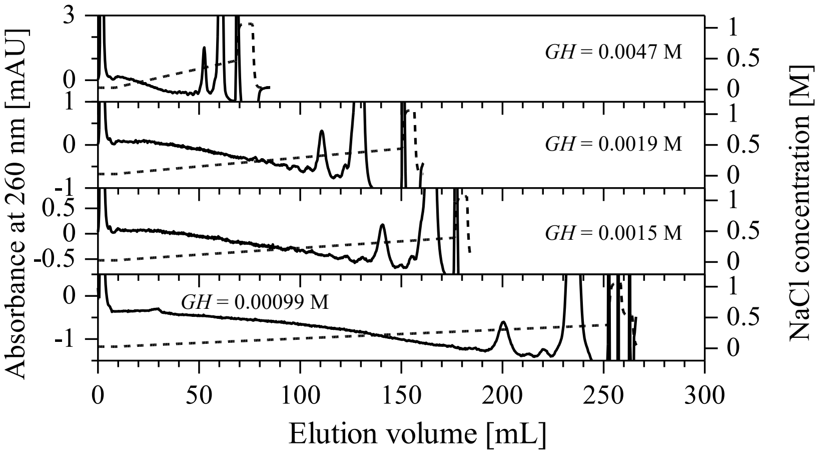

Figure S9 Elution curves of unmodified and modified 20T in linear salt gradient elution using Q Sepharose HP column.

(A) 20T

(B) amino-linked 20T modified at the 5' -end (E-NH_2-20T)

(C) a mixture of PEGylated 20T modified with PEG5K at the 5' -end (E-PEG5K-20T) and amino-linked 20T modified at the 5' -end (E-NH_2-20T)

(D) a mixture of PEGylated 20T modified with PEG10K at the 5' -end (E-PEG10K-20T) and amino-linked 20T modified at the 5' -end (E-NH_2-20T)

(E) amino-linked 20T modified at the 10th thymine (M-NH_2-20T)

(F) a mixture of PEGylated 20T modified with PEG5K at the 10th thymine (M-PEG5K-20T) and amino-linked 20T modified at the 10th thymine (M-NH_2-20T)

(G) a mixture of PEGylated 20T modified with PEG10K at the 10th thymine (M-PEG10K-20T) and amino-linked 20T modified at the 10th thymine (M-NH_2-20T)

The solid and dotted lines represent the absorbance at 260 nm of the column elution and the elution salt concentration, respectively. The salt concentration of the mobile phase was linearly varied from 30 mM to 1 M using a low-salt buffer (10 mM sodium phosphate buffer solution containing 30 mM NaCl, pH 7; buffer A) and a high-salt buffer (10 mM sodium phosphate buffer solution containing 1 M NaCl, pH7; buffer B). The gradient volume of salt concentration (V_g), initial and final salt concentration (I_0 and I_F), *GH*, and peak elution salt concentration (I_R) are summarized in **Table S4**.

Column A (see **Table 2**) was used. The sample injection volume was 100 μL. The sample concentrations are shown in **Table S1**. The flow rate was set to 1.0 mL/min.

(A) Elution curves of 50T

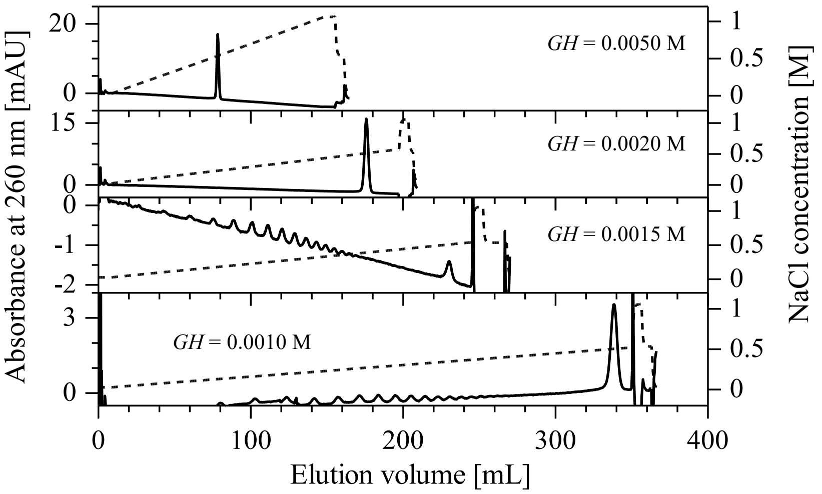

(B) Elution curves of amino-linked 50T modified at the 5' -end (E-NH_2-50T)

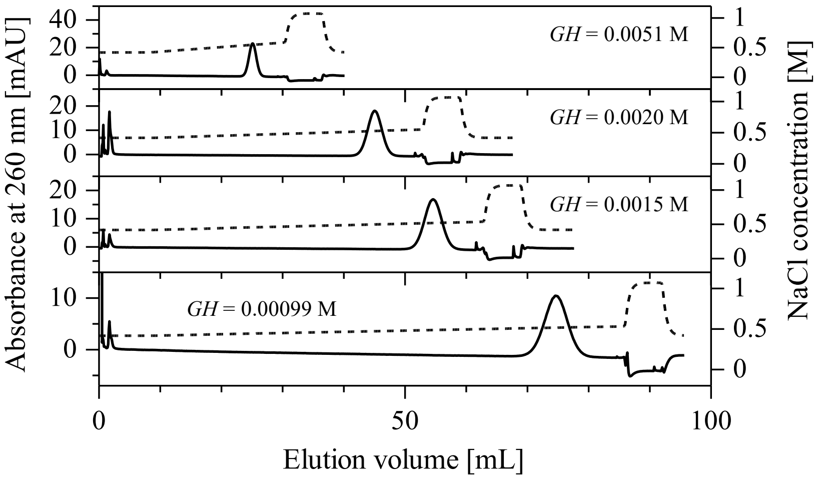

(C) Elution curves of a mixture of PEGylated 50T modified with PEG5K at the 5' -end (E-PEG5K-50T) and amino-linked 50T modified at the 5' -end (E-NH_2-50T)

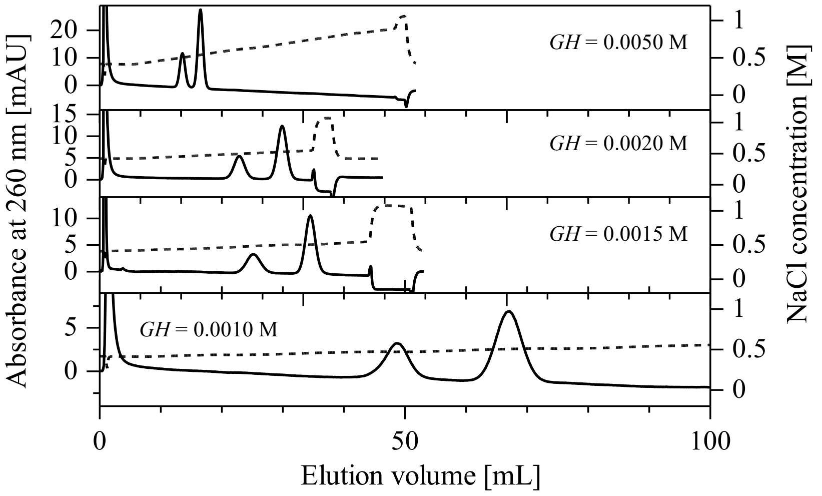

Figure S10 *continued*

(D) Elution curves of a mixture of PEGylated 50T modified with PEG10K at the 5′ -end (E-PEG10K-50T) and amino-linked 50T modified at the 5′ -end (E-NH_2-50T)

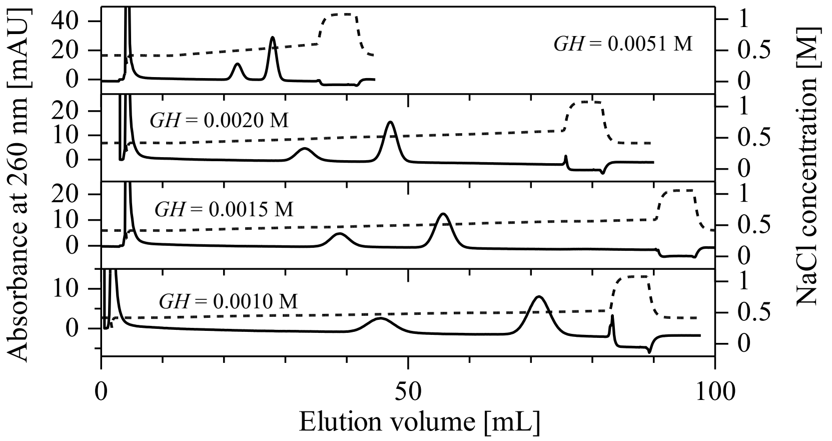

(E) Elution curves of amino-linked 50T modified at the 24th thymine (M-NH_2-50T)

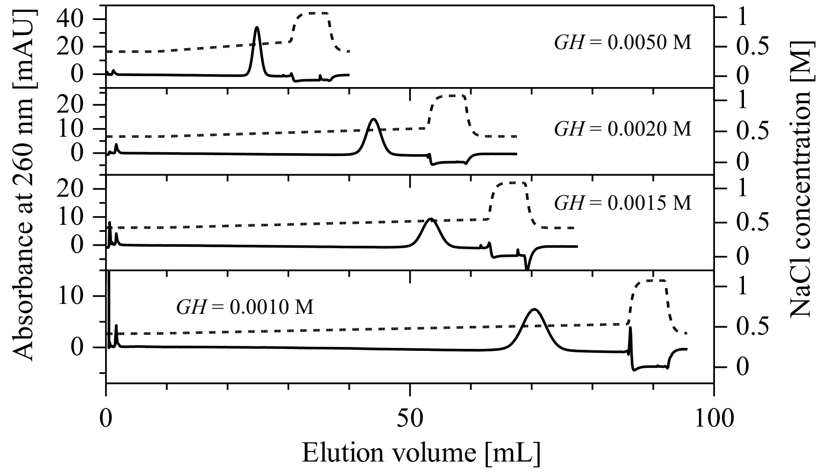

(F) Elution curves of a mixture of PEGylated 50T modified with PEG5K at the 24th thymine (M-PEG5K-50T) and amino-linked 50T modified at the 24th thymine (M-NH_2-50T)

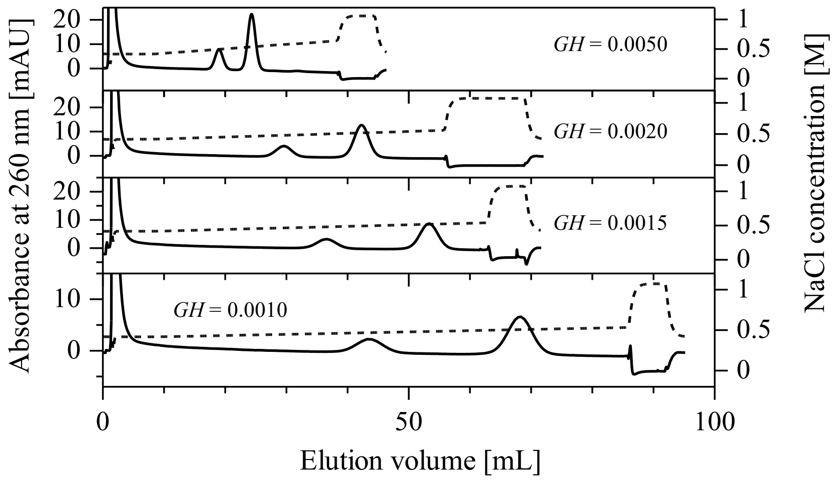

Figure S10 *continued*

(G) Elution curves of a mixture of PEGylated 50T modified with PEG10K at the 24th thymine (M-PEG10K-50T) and amino-linked 50T modified at the 24th thymine (M-NH_2-50T)

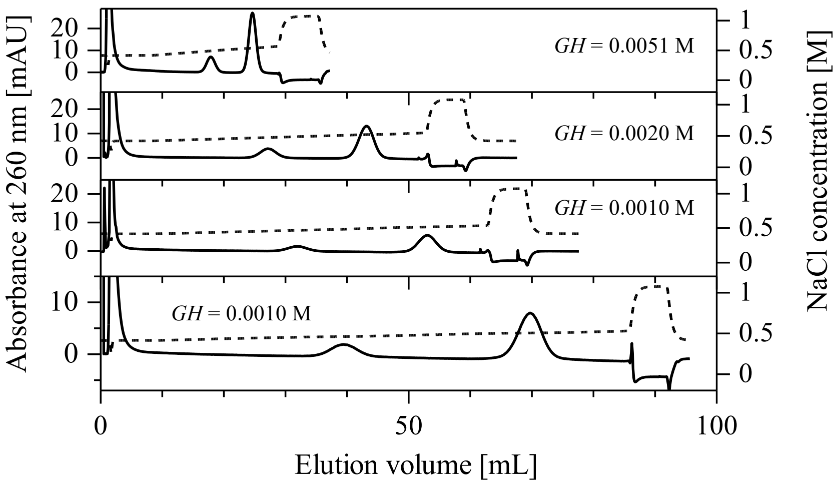

Figure S10. Elution curves of unmodified and modified 50T in linear salt gradient elution using Q Sepharose HP column.

(A) 50T

(B) amino-linked 50T modified at the 5′ -end (E-NH_2-50T)

(C) a mixture of PEGylated 50T modified with PEG5K at the 5′ -end (E-PEG5K-50T) and amino-linked 50T modified at the 5′ -end (E-NH_2-50T)

(D) a mixture of PEGylated 50T modified with PEG10K at the 5′ -end (E-PEG10K-50T) and amino-linked 50T modified at the 5′ -end (E-NH_2-50T)

(E) amino-linked 50T modified at the 24th thymine (M-NH_2-50T)

(F) a mixture of PEGylated 50T modified with PEG5K at the 24th thymine (M-PEG5K-50T) and amino-linked 50T modified at the 24th thymine (M-NH_2-50T)

(G) a mixture of PEGylated 50T modified with PEG10K at the 24th thymine (M-PEG10K-50T) and amino-linked 50T modified at the 24th thymine (M-NH_2-50T)

The solid and dotted lines represent the absorbance at 260 nm of the column elution and the elution salt concentration, respectively. The salt concentration of the mobile phase was linearly varied from 30 mM to 1 M using a low-salt buffer (10 mM sodium phosphate buffer solution containing 30 mM NaCl, pH 7; buffer A) and a high-salt buffer (10 mM sodium phosphate buffer solution containing 1 M NaCl, pH7; buffer B). The gradient volume of salt concentration (V_g), initial and final salt concentration (I_0 and I_F), *GH*, and peak elution salt concentration (I_R) are summarized in **Table S4**.

Column A (see **Table 2**) was used. The sample injection volume was 100 μL. The sample concentrations are shown in **Table S1**. The flow rate was set to1.0 mL/min.

(A) Elution curves of 95T

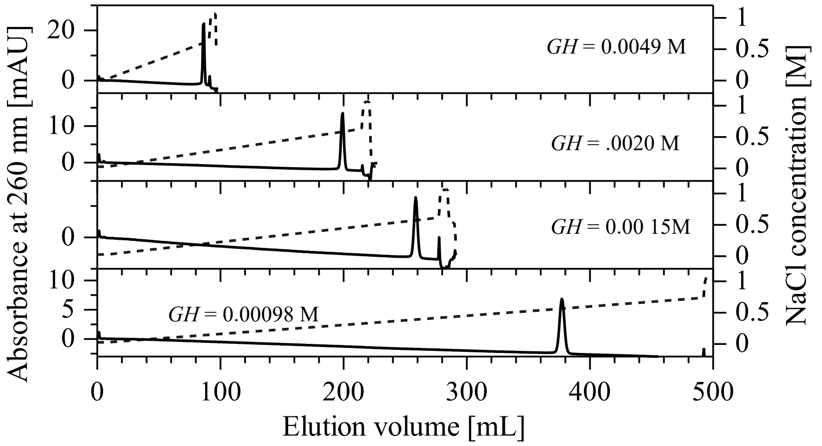

(B) Elution curves of amino-linked 95T modified at the 5′ -end (E-NH2-9T)

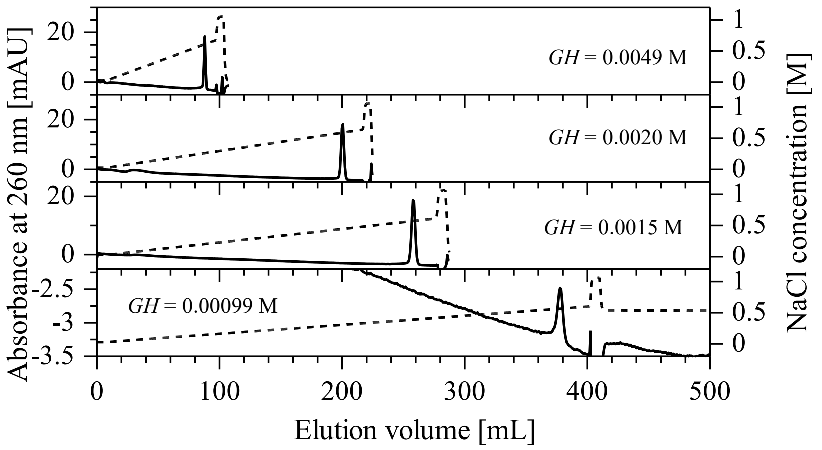

(C) Elution curves of a mixture of PEGylated 95T modified with PEG5K at the 5′ -end (E-PEG5K-95T) and amino-linked 95T modified at the 5′ -end (E-NH2-95T)

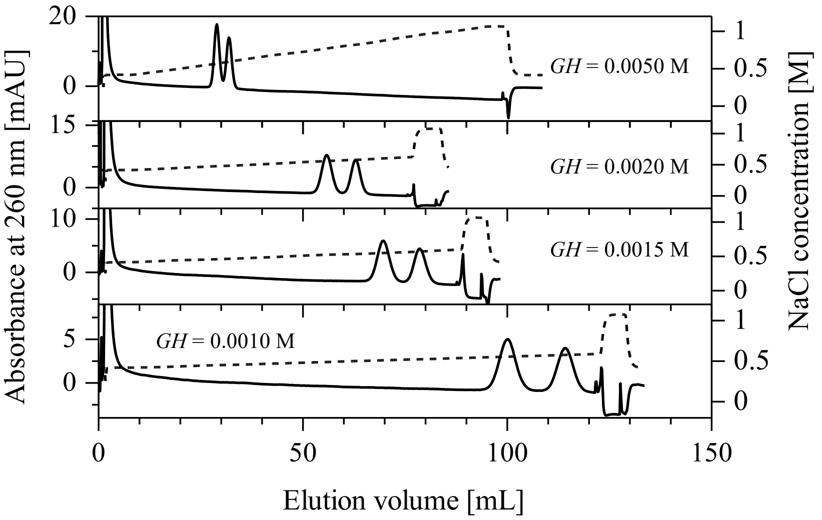

Figure S11 *continued*

(D) Elution curves of a mixture of PEGylated 95T modified with PEG10K at the 5′ -end (E-PEG5K-95T) and amino-linked 95T modified at the 5′ -end (E-NH_2-95T)

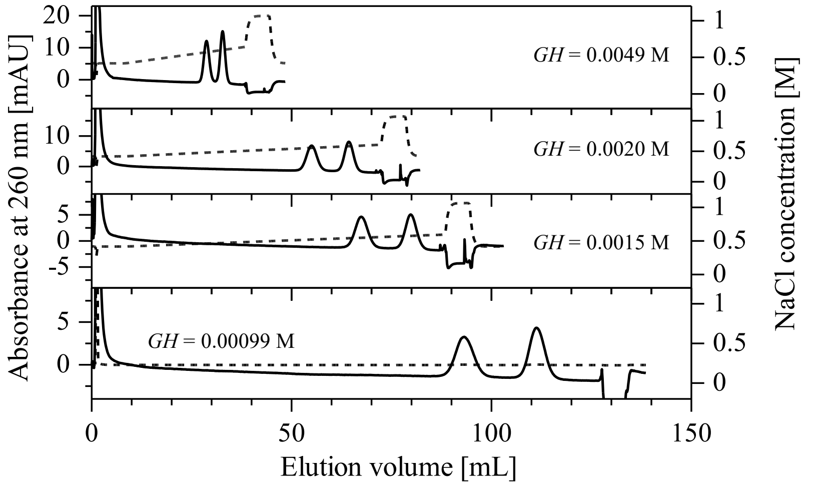

(E) Elution curves of amino-linked 90T modified at the 45th thymine (M-NH_2-90T)

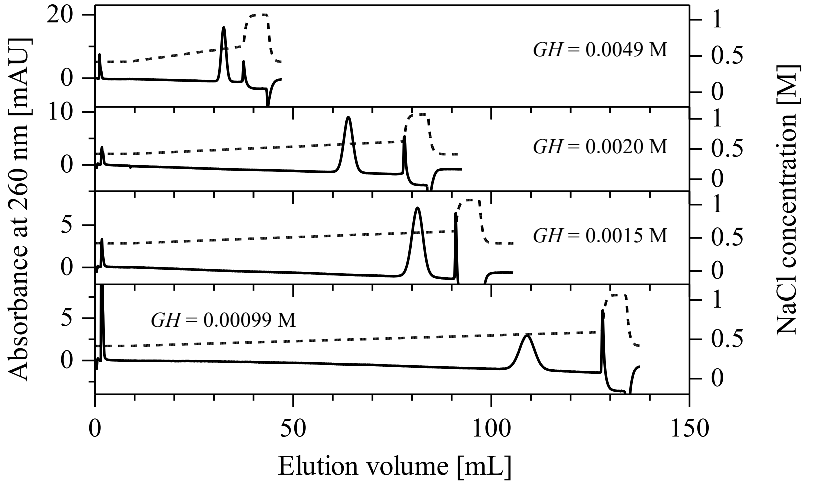

(F) Elution curves of a mixture of PEGylated 90T modified with PEG5K at the 24th thymine (M-PEG5K-90T) and amino-linked 90T modified at the 45th thymine (M-NH_2-90T)

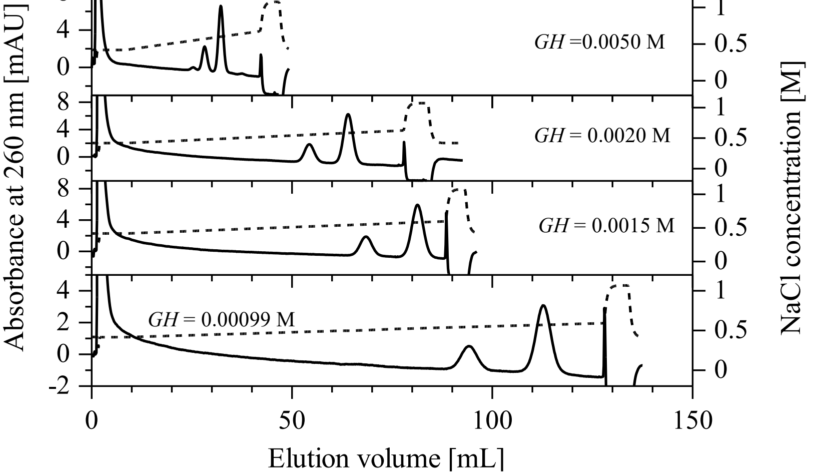

Figure S11 *continued*

(G) Elution curves of a mixture of PEGylated 90T modified with PEG10K at the 45th thymine (M-PEG10K-90T) and amino-linked 90T modified at the 45th thymine (M-NH_2-90T)

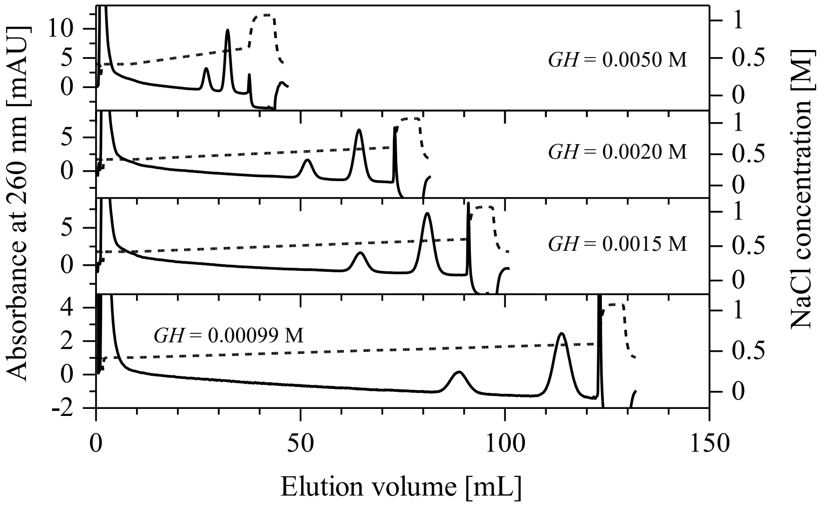

Figure S11. Elution curves of unmodified 95T and modified 95T and 95T in linear salt gradient elution using Q Sepharose HP column.

(A) 95T

(B) amino-linked 95T modified at the 5′ -end (E-NH_2-95T)

(C) a mixture of PEGylated 95T modified with PEG5K at the 5′-end (E-PEG5K-95T) and amino-linked 95T modified at the 5′ -end (E-NH_2-95T)

(D) a mixture of PEGylated 95T modified with PEG10K at the 5′ -end (E-PEG10K-95T) and amino-linked 95T modified at the 5′ -end (E-NH_2-95T)

(E) amino-linked 90T modified at the 45th thymine (M-NH_2-90T)

(F) a mixture of PEGylated 90T modified with PEG5K at the 45th thymine (M-PEG5K-90T) and amino-linked 90T modified at the 45th thymine (M-NH_2-90T)

(G) a mixture of PEGylated 90T modified with PEG10K at the 45th thymine (M-PEG10K-90T) and amino-linked 90T modified at the 45th thymine (M-NH_2-90T)

The solid and dotted lines represent the absorbance at 260 nm of the column elution and the elution salt concentration, respectively. The salt concentration of the mobile phase was linearly varied from 30 mM to 1 M using a low-salt buffer (10 mM sodium phosphate buffer solution containing 30 mM NaCl, pH 7; buffer A) and a high-salt buffer (10 mM sodium phosphate buffer solution containing 1 M NaCl, pH7; buffer B). The gradient volume of salt concentration (V_g), initial and final salt concentration (I_0 and I_F), *GH*, and peak elution salt concentration (I_R) are summarized in **Table S4**.

Column A (see **Table 2**) was used. The sample injection volume was 100 μL. The sample concentrations are shown in **Table S1**. The flow rate was set to 1.0 mL/min.

Table S4. Peak elution salt concentration, I_R and model-derived parameters based on the GH-I_R model under different salt gradient volumes, V_g for unmodified and modified poly(dT) obtained from linear salt gradient elution experiments using Q Sepharose HP column (column A), as shown in **Figures. S8-S11**.

(i) unmodified and modified 9T

V_g [a] [mL]	I_0 [b] [M]	I_F [c] [M]	GH [d] [M]	I_R [e] [M]	$A\times10^3$ [f]	B [g]	K_e [h]	$\|R\|$ [i]	$\frac{K_e}{K_{e,\ 9T}}$ [j]
**(A) 9T**									
140	0.031	1.0	0.0049	0.263 ± 0.001	1.05	6.6	450	0.998	1
350	0.030	1.0	0.0020	0.231 ± 0.003					
467	0.030	1.0	0.0015	0.222 ± 0.001					
700	0.030	1.0	0.00098	0.213 ± 0.001					
**(B) amino-linked 9T at the 5' -end (E-NH$_2$-9T)**									
138	0.030	1.0	0.0050	0.260 ± 0.001	1.10	6.6	480	0.997	1
344	0.031	1.0	0.0020	0.235 ± 0.002					
459	0.031	1.0	0.0015	0.225 ± 0.001					
658	0.031	1.0	0.0010	0.214± 0.000					
**(C) PEGylated 9T modified with PEG5K at the 5' -end (E-PEG5K-9T)**									
138	0.031	1.0	0.0051	0.215 ± 0.002	0.26	6.6	110	0.998	0.236
344	0.030	1.0	0.0020	0.193 ± 0.000					
459	0.031	1.0	0.0015	0.187 ± 0.001					
658	0.031	1.0	0.0010	0.181 ± 0.000					
**(D) PEGylated 9T modified with PEG10K at the 5' -end (E-PEG10K-9T)**									
138	0.031	1.0	0.0050	0.205 ± 0.000	0.19	6.6	82	0.999	0.173
344	0.031	1.0	0.0020	0.186 ± 0.000					
459	0.031	1.0	0.0015	0.179 ± 0.000					
658	0.031	1.0	0.0010	0.171 ± 0.001					
**(E) amino-linked 9T modified at the 5th thymine (M-NH$_2$-9T)**									
138	0.031	1.0	0.0051	0.224 ± 0.001	0.30	6.6	130	0.999	0.273
344	0.031	1.0	0.0020	0.197 ± 0.000					
459	0.030	1.0	0.0015	0.189 ± 0.002					
658	0.031	1.0	0.0011	0.181 ± 0.000					
**(F) PEGylated 9T modified with PEG5K at the 5th thymine (M-PEG5K-9T)**									
138	0.030	1.0	0.0051	0.196 ± 0.001	0.11	6.6	48	0.999	0.100
344	0.031	1.0	0.0020	0.174 ± 0.002					
459	0.030	1.0	0.0015	0.166 ± 0.000					
658	0.031	1.0	0.0011	0.159 ± 0.002					
**(G) PEGylated 9T modified with PEG10K at the 5th thymine (M-PEG10K-9T)**									
138	0.30	1.0	0.0050	0.183 ± 0.001	0.073	6.6	32	0.999	0.066
344	0.31	1.0	0.0020	0.164 ± 0.000					
459	0.31	1.0	0.0015	0.158 ± 0.001					
688	0.31	1.0	0.0011	0.150 ± 0.001					

(ii) unmodified and modified 20T

V_g [a] [mL]	I_0 [b] [M]	I_F [c] [M]	GH [d] [M]	I_R [e] [M]	$A\times10^3$ [f]	B [g]	$K_e\times10^{-6}$ [h]	$\|R\|$ [i]	$\frac{K_e}{K_{e,\ 20T}}$ [j]
**(A) 20T**									
140	0.030	1.0	0.0049	0.412 ± 0.001	0.54	11	1.3	0.992	1
350	0.030	1.0	0.0020	0.387 ± 0.002					
467	0.030	1.0	0.0015	0.376 ± 0.004					
700	0.030	0.99	0.00098	0.359 ± 0.001					
**(B) amino-linked 20T modified at the 5’ -end (E-NH_2-20T)**									
140	0.030	1.0	0.0049	0.405 ± 0.004	0.41	11	1.0	0.999	0.741
350	0.030	0.99	0.0020	0.375 ± 0.002					
467	0.030	1.0	0.0018	0.364 ± 0.004					
700	0.030	1.0	0.00098	0.352 ± 0.001					
**(C) PEGylated 20T modified with PEG5K at the 5’ -end (E-PEG5K-20T)**									
140	0.031	1.0	0.0050	0.363 ± 0.002	0.29	11	0.72	0.997	0.523
350	0.030	1.0	0.0020	0.339 ± 0.001					
467	0.031	1.0	0.0015	0.329 ± 0.001					
700	0.030	1.0	0.00099	0.322 ± 0.000					
**(D) PEGylated 20T modified with PEG10K at the 5’ -end (E-PEG10K-20T)**									
140	0.031	1.0	0.0050	0.349 ± 0.002	0.064	11	0.16	0.999	0.116
350	0.031	1.0	0.0020	0.328 ± 0.000					
467	0.031	1.0	0.0015	0.321 ± 0.000					
700	0.031	1.0	0.00099	0.312 ± 0.001					
**(E) amino-linked 20T modified at the 10th thymine (M-NH_2-20T)**									
140	0.030	1.0	0.0047	0.395 ± 0.001	0.053	11	0.13	0.998	0.096
350	0.030	1.0	0.0019	0.361 ± 0.001					
467	0.030	1.0	0.0014	0.354 ± 0.001					
700	0.030	1.0	0.00098	0.342 ± 0.003					
**(F) PEGylated 20T modified with PEG5K at the 10th thymine (M-PEG5K-20T)**									
140	0.031	1.0	0.0049	0.347 ± 0.002	0.11	11	0.27	0.999	0.199
350	0.030	1.0	0.0020	0.319 ± 0.000					
467	0.030	1.0	0.0015	0.312 ± 0.001					
700	0.030	0.99	0.00098	0.300 ± 0.003					
**(G) PEGylated 20T modified with PEG10K at the 10th thymine (M-PEG10K-20T)**									
140	0.031	1.0	0.0047	0.340 ± 0.000	0.073	11	0.18	0.993	0.132
350	0.031	1.0	0.0019	0.312 ± 0.002					
460	0031	1.0	0.0014	0.309 ± 0.005					
700	0.030	1.0	0.00099	0.295 ± 0.001					

(iii) unmodified and modified 50T

V_g [a] [mL]	I_0 [b] [M]	I_F [c] [M]	GH [d] [M]	I_R [e] [M]	$A\times10^6$ [f]	B [g]	$K_e\times10^{-13}$ [h]	$\|R\|$ [i]	$\frac{K_e}{K_{e,\ 50T}}$ [j]
**(A) 50T**									
140	0.030	1.0	0.0050	0.514 ± 0.000	1.8	22	1.1	0.991	1
350	0.030	1.0	0.0020	0.498 ± 0.007					
467	0.030	1.0	0.0015	0.489 ± 0.001					
700	0.030	1.0	0.0010	0.480 ± 0.003					
**(B) amino-linked poly 50T at the 5' -end (E-NH$_2$-50T)**									
87	0.40	1.0	0.0051	0.524 ± 0.001	2.9	22	1.8	0.991	1.62
216	0.40	1.0	0.0020	0.504 ± 0.000					
289	0.40	1.0	0.0015	0.500 ± 0.003					
433	0.40	1.0	0.0010	0.495 ± 0.001					
**(C) PEGylated 50T modified with PEG5K at the 5'- end (E-PEG5K-50T)**									
87	0.40	1.0	0.0050	0.485 ± 0.004	0.50	22	0.31	0.969	0.277
216	0.40	1.0	0.0020	0.474 ± 0.002					
289	0.40	1.0	0.0015	0.463 ± 0.001					
433	0.40	1.0	0.0010	0.453 ± 0.002					
**(D) PEGylated 50T modified with PEG10K at the 5' -end (E-PEG10K-50T)**									
87	0.4	1.0	0.0051	0.475 ± 0.001	0.35	22	0.21	0.994	0.196
216	0.4	1.0	0.0020	0.461 ± 0.000					
289	0.4	1.0	0.0015	0.458 ± 0.004					
433	0.4	1.0	0.0010	0.454 ± 0.000					
**(E) amino-linked 50T modified at the 24th thymine (M-NH2-50T)**									
87	0.4	1.0	0.0050	0.519 ± 0.003	2.6	22	1.6	0.988	1.42
216	0.4	1.0	0.0020	0.504 ± 0.004					
289	0.4	1.0	0.0015	0.499 ± 0.001					
433	0.4	1.0	0.00099	0.488 ± 0.001					
**(F) PEGylated 50T modified with PEG5K at the 24th thymine (M-PEG5K-50T)**									
87	0.4	1.0	0.0050	0.478 ± 0.003	0.355	22	0.22	0.988	0.197
216	0.4	1.0	0.0020	0.462 ± 0.003					
289	0.4	1.0	0.0015	0.460 ± 0.001					
433	0.4	1.0	0.0010	0.448 ± 0.001					
**(G) PEGylated 50T modified with PEG10K at the 24th thymine (M-PEG10K-50T)**									
87	0.4	1.0	0.0051	0.471 ± 0.001	0.233	22	0.14	0.999	0.129
216	0.4	1.0	0.0020	0.454 ± 0.001					
289	0.4	1.0	0.0015	0.449 ± 0.000					
433	0.4	1.0	0.0010	0.441 ± 0.001					

(iv) unmodified and modified 90T or 95T

V_g [a] [mL]	I_0 [b] [M]	I_F [c] [M]	GH [d] [M]	I_R [e] [M]	$A\times10^6$ [f]	B [g]	$K_e\times10^{-17}$ [h]	$\|R\|$ [i]	$\frac{K_e}{K_{e,\ 95T}}$ [j]
**(A) 95T**									
140	0.031	1.0	0.0049	0.573 ± 0.000	1.08	27	1.2	0.999	1
350	0.031	1.0	0.0020	0.555 ± 0.003					
467	0.030	1.0	0.0015	0.548 ± 0.000					
700	0.030	1.0	0.00099	0.541 ± 0.002					
**(B) amino-linked 95T modified at the 5’ -end (E-NH2-95T)**									
140	0.031	1.0	0.0049	0.574 ± 0.007	1.30	27	1.5	0.979	1.16
350	0.031	1.0	0.0020	0.561 ± 0.003					
467	0.031	0.99	0.0015	0.554 ± 0.007					
700	0.031	1.0	0.00099	0.542 ± 0.003					
**(C) PEGylated 95T modified with PEG5K at the 5’ -end (E-PEG5K-95T)**									
87	0.40	1.0	0.0049	0.546 ± 0.004	0.399	27	0.45	0.972	0.179
216	0.40	1.0	0.0020	0.538 ± 0.003					
289	0.40	1.0	0.0015	0.532 ± 0.007					
433	0.40	1.0	0.0010	0.524 ± 0.006					
**(D) PEGylated 95T modified with PEG10K at the 5’ -end (E-PEG10K-95T)**									
87	0.40	1.0	0.0049	0.544 ± 0.001	0.303	27	0.34	0.981	0.158
216	0.40	1.0	0.0020	0.533 ± 0.002					
289	0.40	1.0	0.0015	0.523 ± 0.000					
433	0.40	1.0	0.00099	0.519 ± 0.002					
**(E) amino-linked 90T modified at the 45th thymine (M-NH2-90T)**									
87	0.40	1.0	0.0049	0.569 ± 0.001	1.18	27	1.34	0.981	1.05
216	0.40	1.0	0.0020	0.559 ± 0.002					
289	0.40	1.0	0.0015	0.550 ± 0.003					
433	0.40	1.0	0.00099	0.544 ± 0.005					
**(F) PEGylated 90T modified with PEG5K at the 45th thymine (M-PEG5K-90T)**									
87	0.40	1.0	0.0050	0.544 ± 0.001	0.322	27	0.37	0.989	0.294
216	0.40	1.0	0.0020	0.531 ± 0.000					
289	0.40	1.0	0.0015	0.527 ± 0.000					
433	0.40	1.0	0.00099	0.520 ± 0.000					
**(G) PEGylated 90T modified with PEG10K at the 45th thymine (M-PEG10K-90T)**									
87	0.40	1.0	0.0050	0.534 ± 0.001	0.197	27	0.22	0.983	0.179
216	0.40	1.0	0.0020	0.521 ± 0.000					
289	0.40	1.0	0.0015	0.517 ± 0.002					
433	0.40	1.1	0.00099	0.515 ± 0.001					

[a]V_g: Gradient volume from the initial salt concentration (I_0) to the final salt concentration (I_f) in a linear salt gradient elution experiment.

[b]I_0: Initial salt concentration.

[c]I_f: Final salt concentration.

[d]GH: Normalized gradient slope, calculated as $GH = \frac{I_0 - I_f}{V_g/V_s}$ where V_s is the stationary phase volume.

[e]I_R: Peak elution salt concentration.

[f]A: Fitting parameter of the GH-IR model described by **Equation (2)**.

[g]B: Number of the binding sites of the solute to the stationary phase.

[h]K_e: Ion exchange reaction constant.

[i]$|R|$: Correlation coefficient based on **Equation (2)**.

[j]$\frac{K_e}{K_{e, ref}}$: Ratio of ion-exchange reaction constant of modified poly(dT) to that of corresponding unmodified reference (e.g., 9T, 20T, 50T, 90T, or 95T).

5. Data from linear salt gradient elution experiments using QA CIM monolith column.

(A) Elution curves of 9T

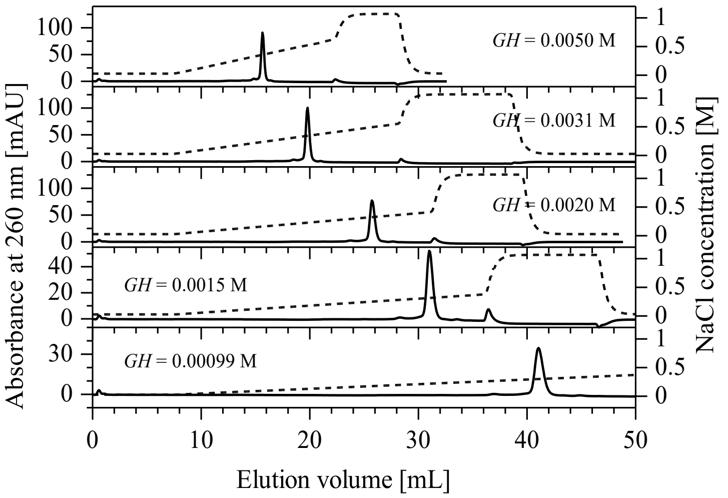

(B) Elution curves of amino-linked 9T modified at the 5′ -end (E-NH_2-9T)

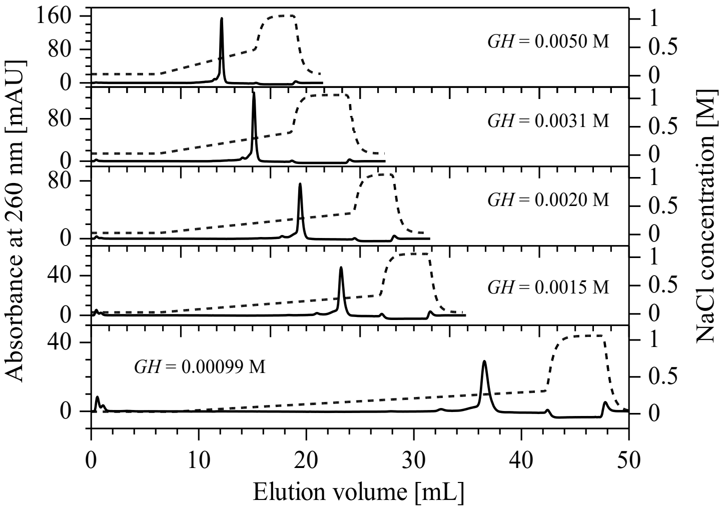

(C) Elution curves of a mixture of PEGylated 9T modified with PEG5K at the 5′ -end (E-PEG5K-9T) and amino-linked 9T modified at the 5′ -end (E-NH_2-9T)

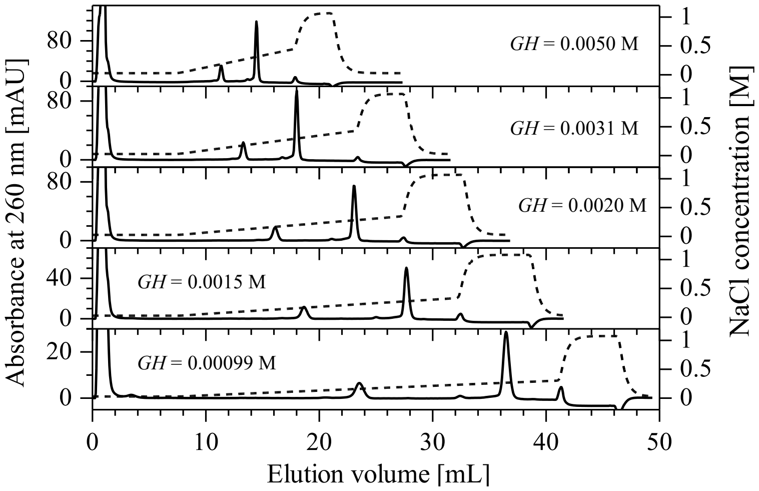

Figure S12 *continued*

(D) Elution curves of a mixture of PEGylated 9T modified with PEG10K at the 5′ -end (E-PEG10K-9T) and amino-linked 9T modified at the 5′ -end (E-NH_2-9T).

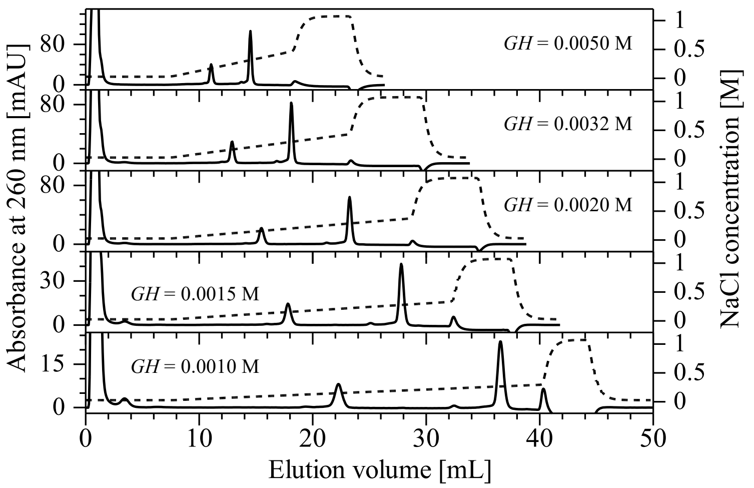

(E) Elution curves of amino-linked 9T modified at the 5th thymine (M-NH_2-9T)

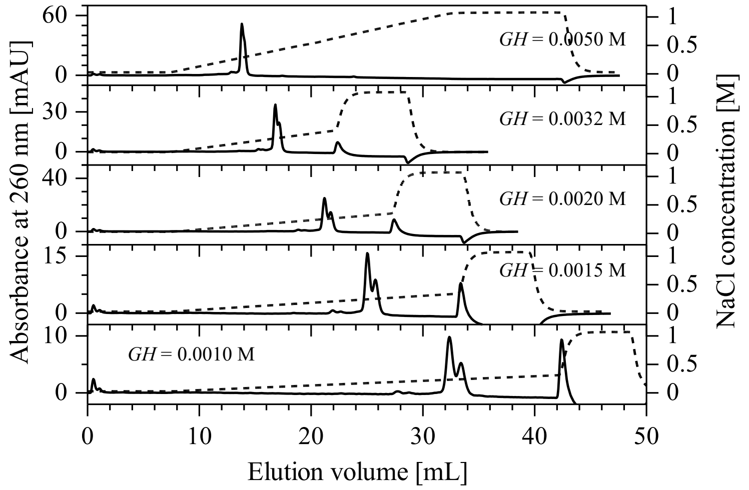

(F) Elution curves of a mixture of PEGylated 9T modified with PEG5K at the 5th thymine (M-PEG5K-9T) and amino-linked 9T modified at the 5th thymine (M-NH_2-9T)

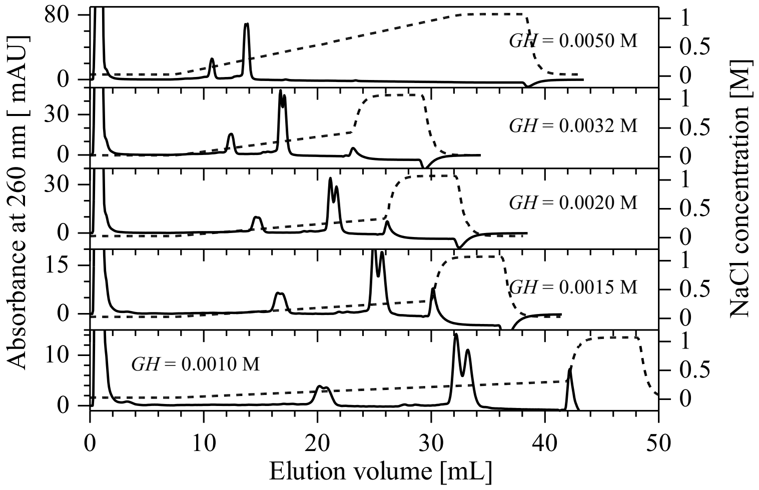

Figure S12 *continued*

(G) Elution curves of a mixture of PEGylated 9T modified with PEG5K at the 5th thymine (M-PEG5K-9T) and amino-linked 9T modified at the 5th thymine (M-NH_2-9T)

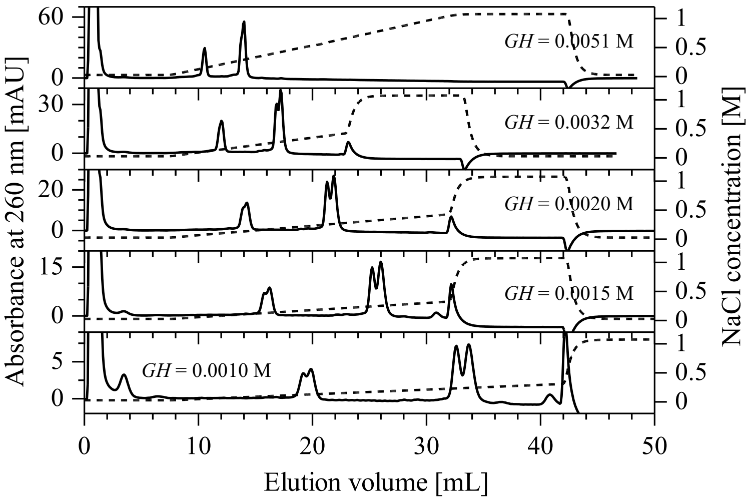

Figure S12. Elution curves of unmodified and modified 9T in linear salt gradient elution using QA monolith column.

(A) 9T

(B) amino-linked 9T modified at the 5′ -end (E-NH_2-9T)

(C) a mixture of PEGylated 9T modified with PEG5K at the 5′ -end (E-PEG5K-9T) and amino-linked 9T modified at the 5′- end (E-NH_2-9T)

(D) a mixture of PEGylated 9T modified with PEG10K at the 5′ -end (E-PEG10K-9T) and amino-linked 9T modified at the 5′-end (E-NH_2-9T)

(E) amino-linked 9T modified at the 5th thymine (M-NH_2-9T)

(F) a mixture of PEGylated 9T modified with PEG5K at the 5th thymine (M-PEG5K-9T) and amino-linked 9T modified at the 5th thymine (M-NH_2-9T)

(G) a mixture of PEGylated 9T modified with PEG10K at the 5th thymine (M-PEG10K-9T) and amino-linked 9T modified at the 5th thymine (M-NH_2-9T)

The solid and dotted lines represent the absorbance at 260 nm of the column elution and the elution salt concentration, respectively. The salt concentration of the mobile phase was linearly varied from 30 mM to 1 M using a low-salt buffer (10 mM sodium phosphate buffer solution containing 30 mM NaCl, pH 7; buffer A) and a high-salt buffer (10 mM sodium phosphate buffer solution containing 1 M NaCl, pH7; buffer B). The gradient volume of the salt concentration (V_g), initial and final salt concentration (I_0 and I_F), *GH*, and peak elution salt concentration (I_R) are summarized in **Table S5**.

Column C (see **Table 2**) was used. The sample injection volume was 100 μL. The sample concentrations are shown in **Table S1**. The flow rate was set to 1 mL/min.

(A) Elution curves of 20T

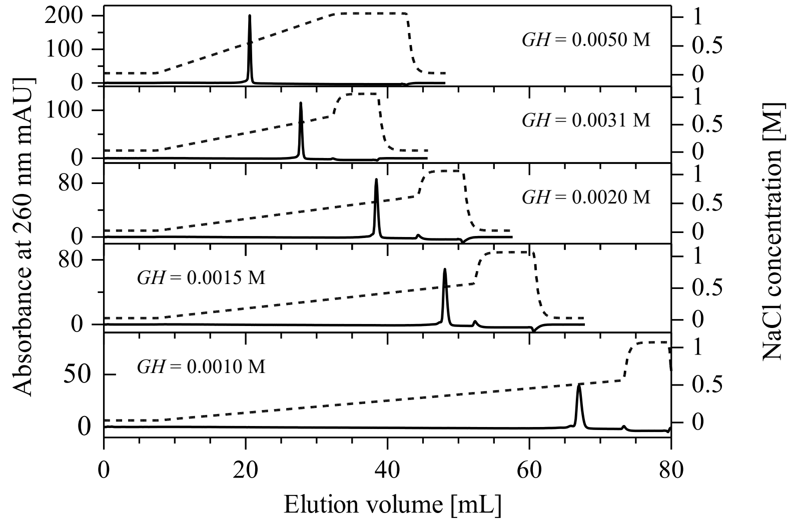

(B) Elution curves of amino-linked 20T modified at the 5′ -end (E-NH_2-20T).

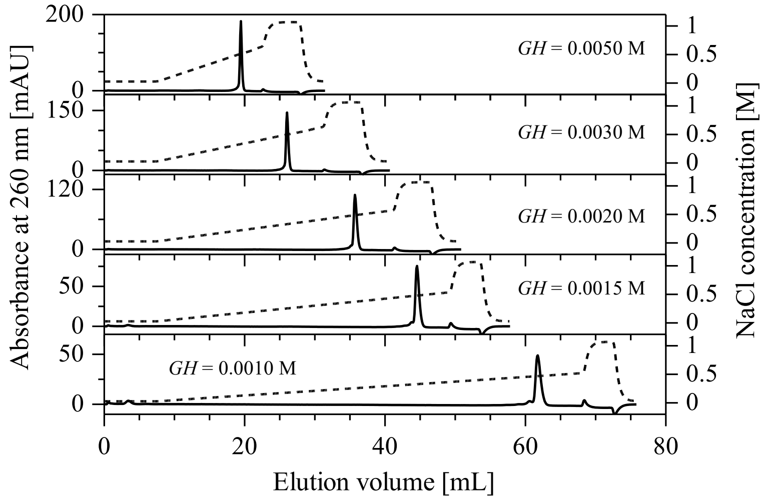

(C) Elution curves of a mixture of PEGylated 20T modified with PEG5K at the 5′ -end (E-PEG5K-20T) and amino-linked 20T modified at the 5′ -end (E-NH_2-20T).

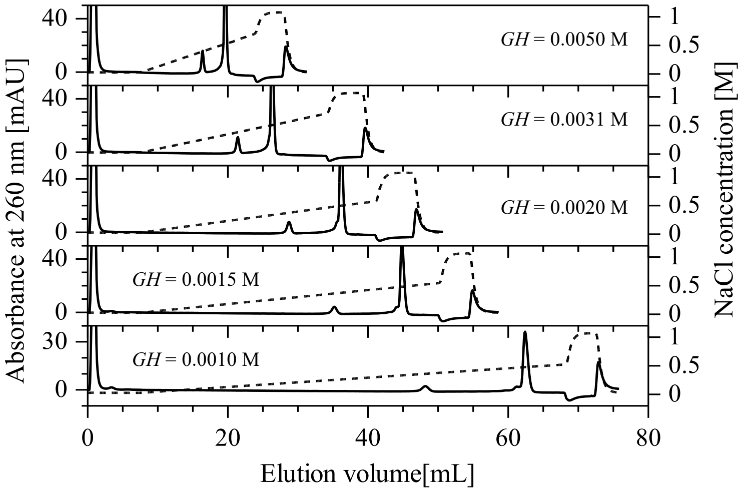

Figure S13 *continued*

(D) Elution curves of a mixture of PEGylated 20T modified with PEG10K at the 5′ -end (E-PEG10K-20T) and amino-linked 20T modified at the 5′ -end (E-NH_2-20T)

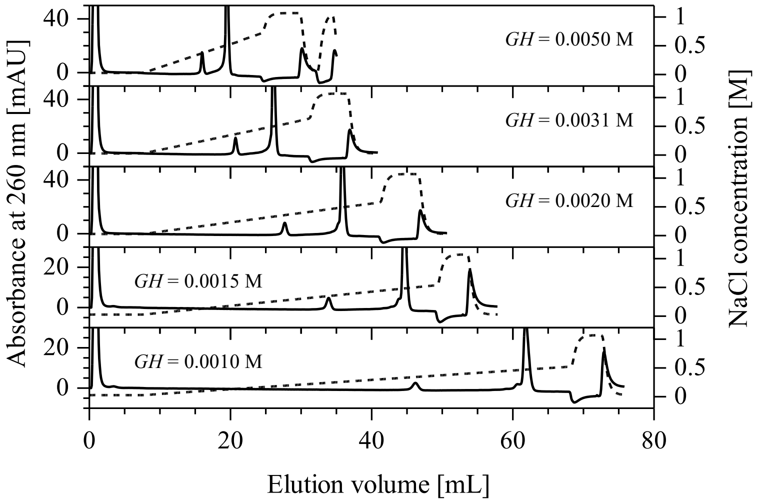

(E) Elution curves of amino-linked 20T modified at the 10th thymine (M-NH_2-20T)

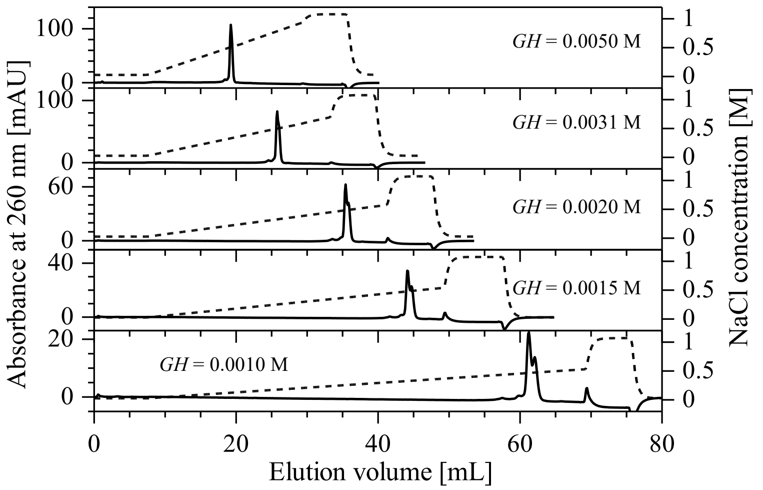

(F) Elution curves of a mixture of PEGylated 20T modified with PEG5K at the 10th thymine (M-PEG5K-20T) and amino-linked 20T modified at the 10th thymine (M-NH_2-20T)

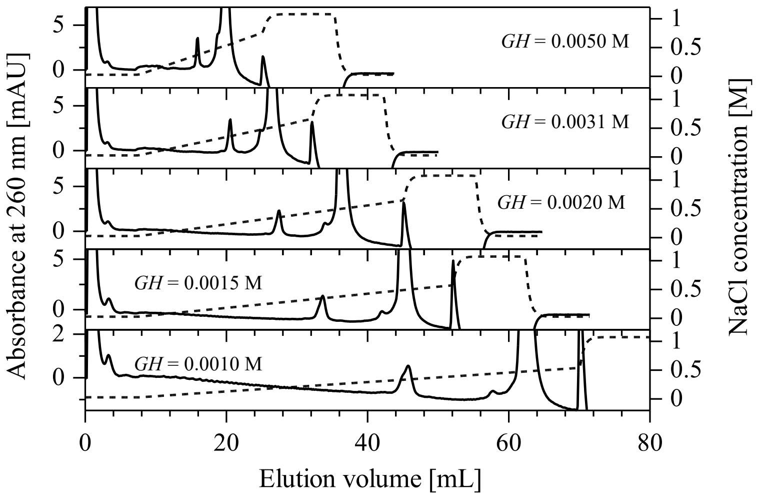

Figure S13 *continued*

(G) Elution curves of a mixture of PEGylated 20T modified with PEG10K at the 10th thymine (M-PEG10K-20T) and amino-linked 20T modified at the 10th thymine (M-NH_2-20T)

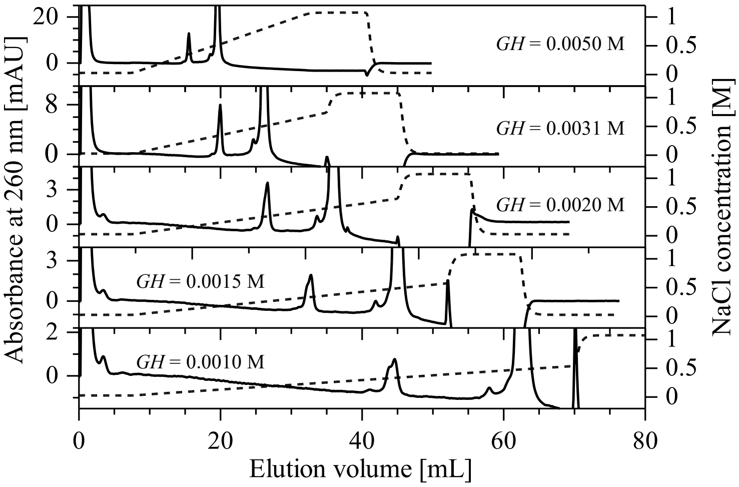

Figure S13. Elution curves of unmodified and modified 20T in linear salt gradient elution using QA monolith column.

(A) 20T

(B) amino-linked 9T modified at the 5′ -end (E-NH_2-20T)

(C) a mixture of PEGylated 20T modified with PEG5K at the 5′-end (E-PEG5K-20T) and amino-linked 20T modified at the 5′ -end (E-NH_2-20T)

(D) a mixture of PEGylated 20T modified with PEG10K at the 5′ -end (E-PEG10K-20T) and amino-linked 20T modified at the 5′ -end (E-NH_2-20T)

(E) amino-linked 20T modified at the 10th thymine (M-NH_2-20T)

(F) a mixture of PEGylated 20T modified with PEG5K at the 10th thymine (M-PEG5K-20T) and amino-linked 20T modified at the 10th thymine (M-NH_2-20T)

(G) a mixture of PEGylated 20T modified with PEG10K at the 10th thymine (M-PEG10K-20T) and amino-linked 20T modified at the 10th thymine (M-NH_2-20T)

The solid and dotted lines represent the absorbance at 260 nm of the column elution and the elution salt concentration, respectively. The salt concentration of the mobile phase was linearly varied from 30 mM to 1 M using a low-salt buffer (10 mM sodium phosphate buffer solution containing 30 mM NaCl, pH 7; buffer A) and a high-salt buffer (10 mM sodium phosphate buffer solution containing 1 M NaCl, pH7; buffer B). The gradient volume of salt concentration (V_g), initial and final salt concentration (I_0 and I_F), *GH*, and peak elution salt concentration (I_R) are summarized in **Table S5**.

Column C (see **Table 2**) was used. The sample injection volume was 100 μL. The sample concentrations are shown in **Table S1**. The flow rate was set to 5.0 mL/min.

(A) Elution curves of 50T

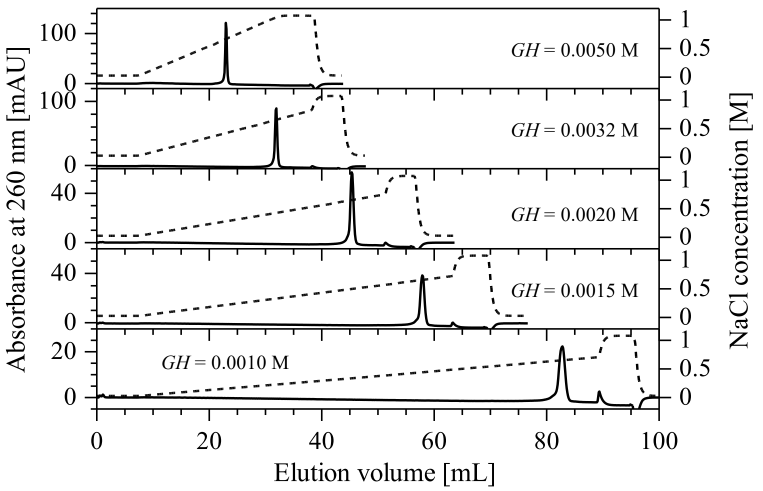

(B) Elution curves of amino-linked 50T modified at the 5′ -end (E-NH_2-50T)

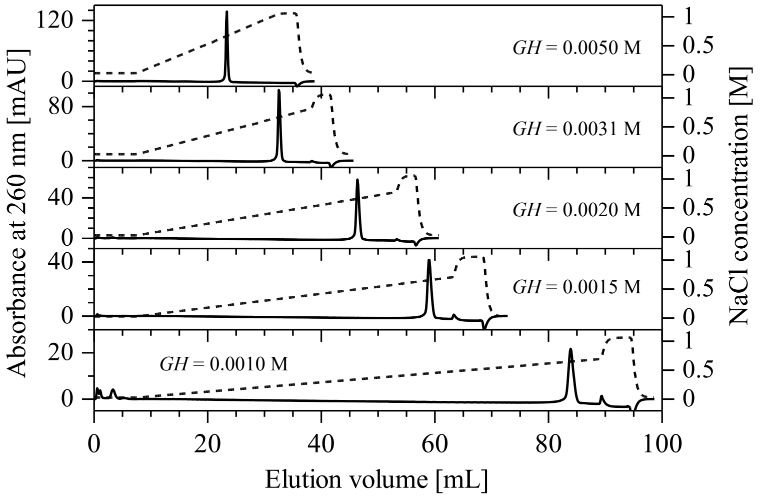

(C) Elution curves of a mixture of PEGylated 50T modified with PEG5K at the 5′ -end (E-PEG5K-50T) and amino-linked 50T modified at the 5′ -end (E-NH_2-50T)

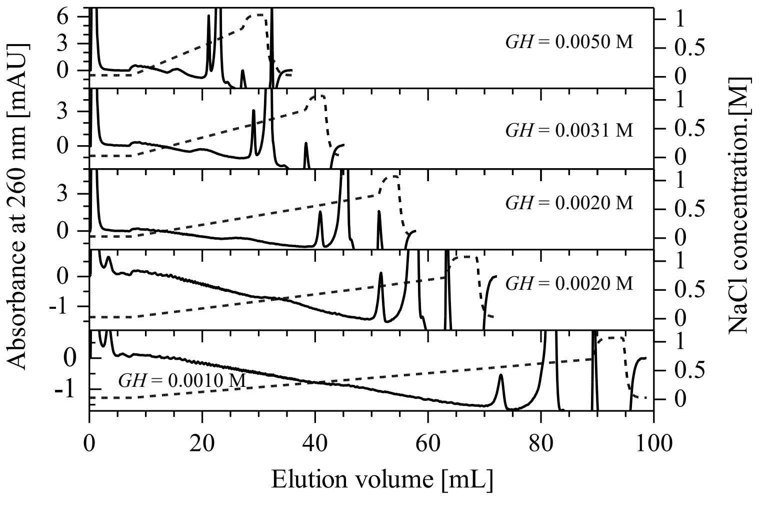

Figure S14 *continued*

(D) Elution curves of a mixture of PEGylated 50T modified with PEG10K at the 5′-end (E-PEG10K-50T) and amino-linked 50T modified at the 5′-end (E-NH_2-50T)

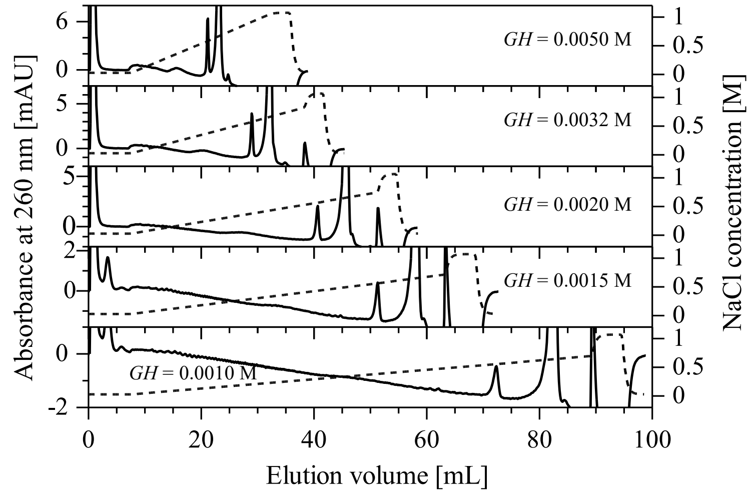

(E) Elution curves of amino-linked 50T modified at the 24th thymine (M-NH_2-50T)

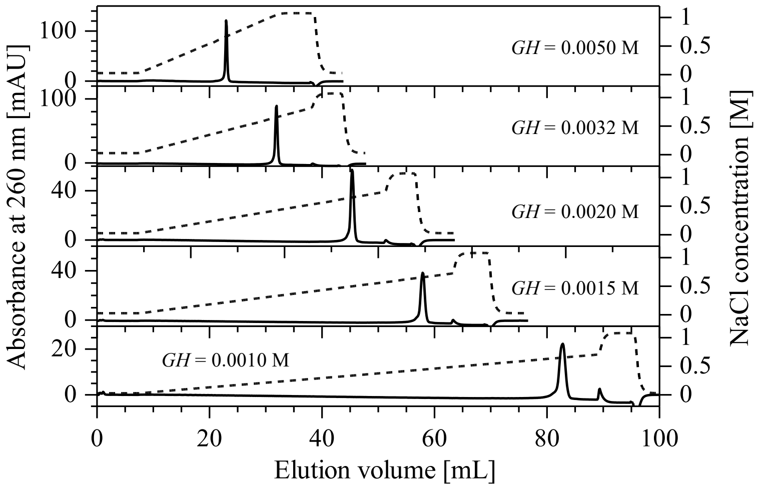

(F) Elution curves of a mixture of PEGylated 50T modified with PEG5K at the 24th thymine (M-PEG5K-50T) and amino-linked 50T modified at the 24th thymine (M-NH_2-50T)

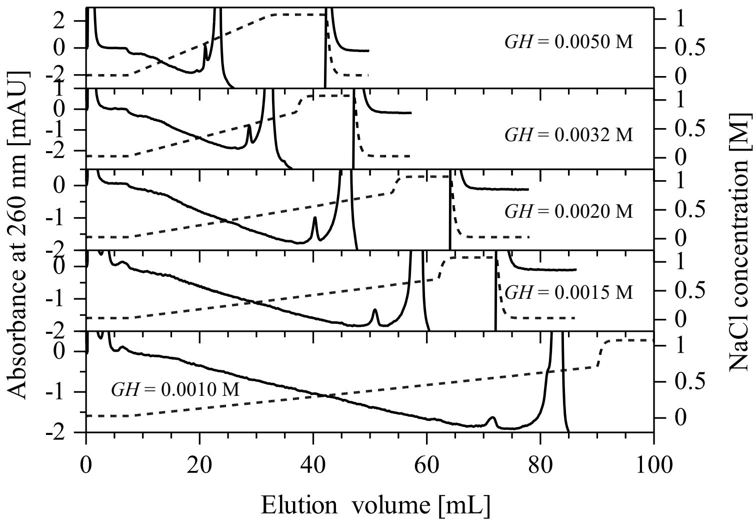

Figure S14 *continued*

(G) Elution curves of a mixture of PEGylated 50T modified with PEG10K at the 24th thymine (M-PEG10K-50T) and amino-linked 50T modified at the 24th thymine (M-NH_2-50T)

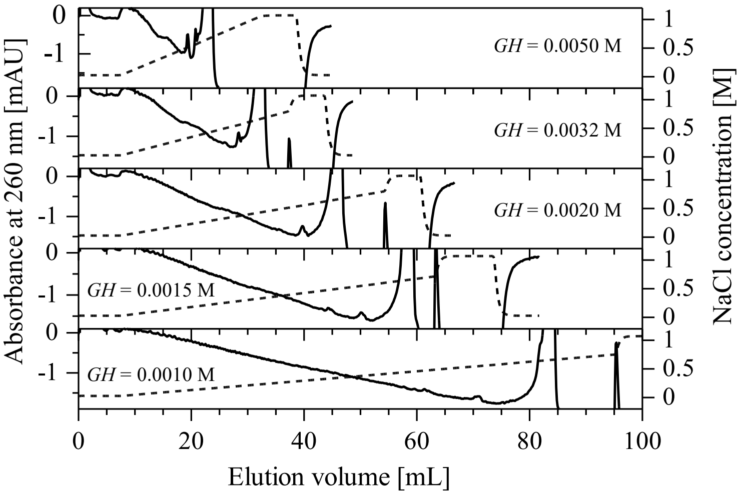

Figure S14. Elution curves of unmodified and modified 50T in linear salt gradient elution using a QA monolith column.

(A) 50T

(B) amino-linked 9T modified at the 5′ -end (E-NH_2-50T)

(C) a mixture of PEGylated 50T modified with PEG5K at the 5′-end (E-PEG5K-50T) and amino-linked 20T modified at the 5′ -end (E-NH_2-50T)

(D) a mixture of PEGylated 20T modified with PEG10K at the 5′ end (E-PEG10K-50T) and amino-linked 20T modified at the 5′ -end (E-NH_2-20T)

(E) amino-linked 50T modified at the 10th thymine (M-NH_2-50T)

(F) PEGylated 50T modified with PEG5K at the 24th thymine (M-PEG5K-50T) and amino-linked 20T modified at the 24th thymine (M-NH_2-50T)

(G) PEGylated 50T modified with PEG10K at the 10th thymine (M-PEG10K-50T) and amino-linked 20T modified at the 24th thymine (M-NH_2-50T)

The solid and dotted lines represent the absorbance at 260 nm of the column elution and the elution salt concentration, respectively. The salt concentration of the mobile phase was linearly varied from 30 mM to 1 M using a low-salt buffer (10 mM sodium phosphate buffer solution containing 30 mM NaCl, pH 7; buffer A) and a high-salt buffer (10 mM sodium phosphate buffer solution containing 1 M NaCl, pH7; buffer B). The gradient volume of salt concentration (V_g), initial and final salt concentration (I_0 and I_F), *GH*, and peak elution salt concentration (I_R) are summarized in **Table S5**.

Column C (see **Table 2**) was used. The sample injection volume was 100 μL. The sample concentrations are shown in **Table S1**. The flow rate was set to 5.0 mL/min.

(A) Elution curves of 95T

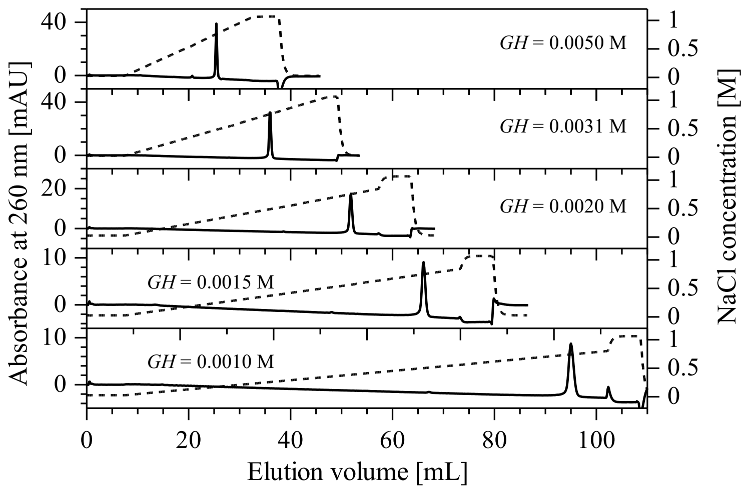

(B) Elution curves of amino-linked 95T modified at the 5′ -end (E-NH_2-9T)

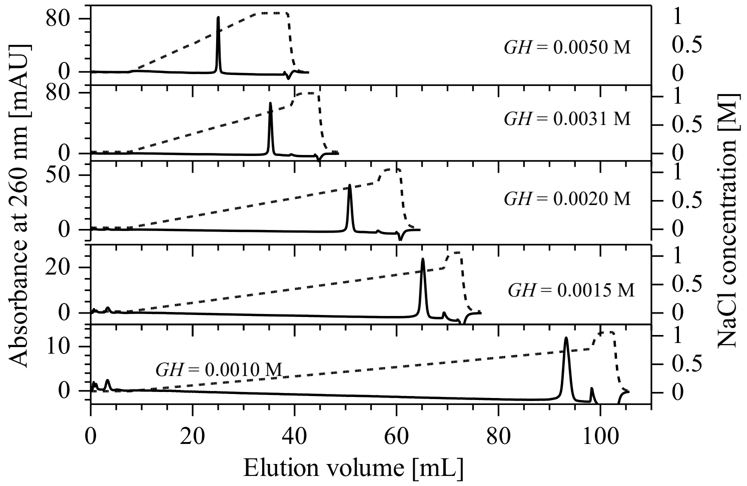

(C) Elution curves of a mixture of PEGylated 95T modified with PEG5K at the 5′ -end (E-PEG5K-95T) and amino-linked 95T modified at the 5′ -end (E-NH_2-95T)

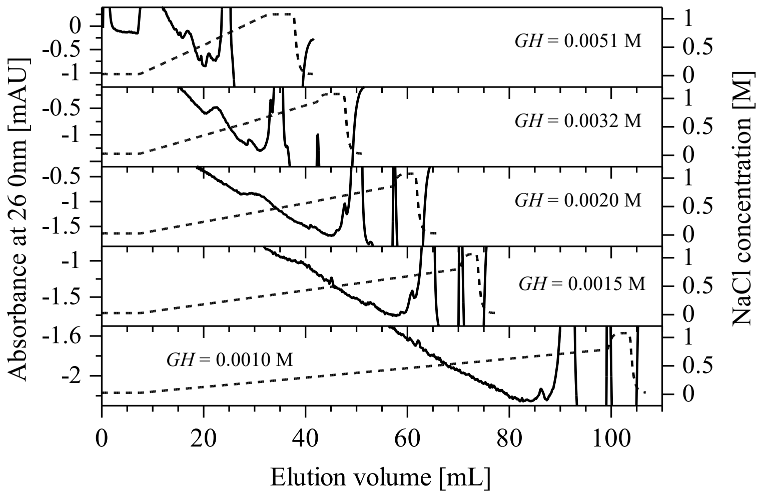

Figure S15 *continued*

(D) Elution curves of a mixture of PEGylated 95T modified with PEG10K at the 5′-end (E-PEG5K-95T) and amino-linked 95T modified at the 5′-end (E-NH_2-95T)

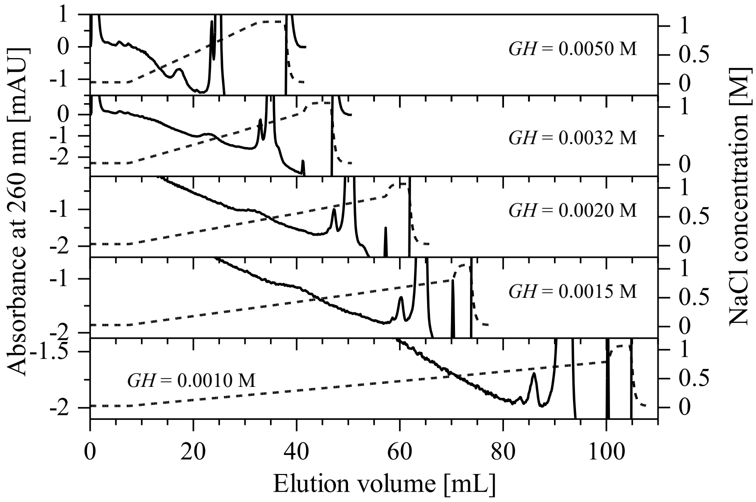

(E) Elution curves of amino-linked 90T modified at the 45th thymine (M-NH_2-90T)

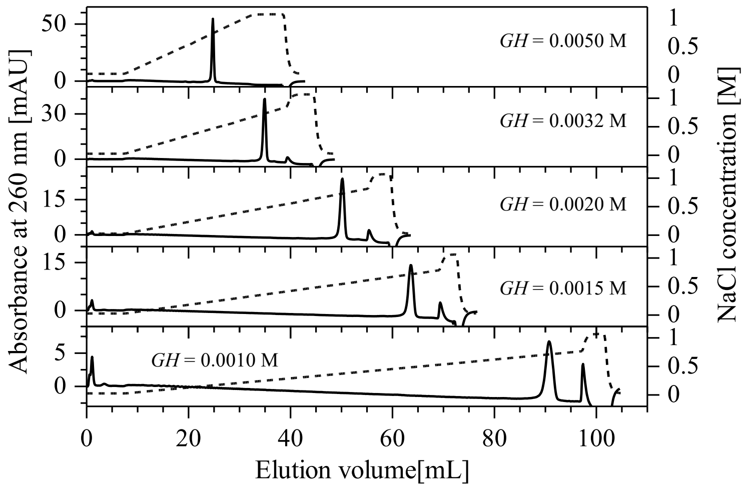

(F) Elution curves of a mixture of PEGylated 90T modified with PEG5K at the 24th thymine (M-PEG5K-90T) and amino-linked 90T modified at the 45th thymine (M-NH_2-90T)

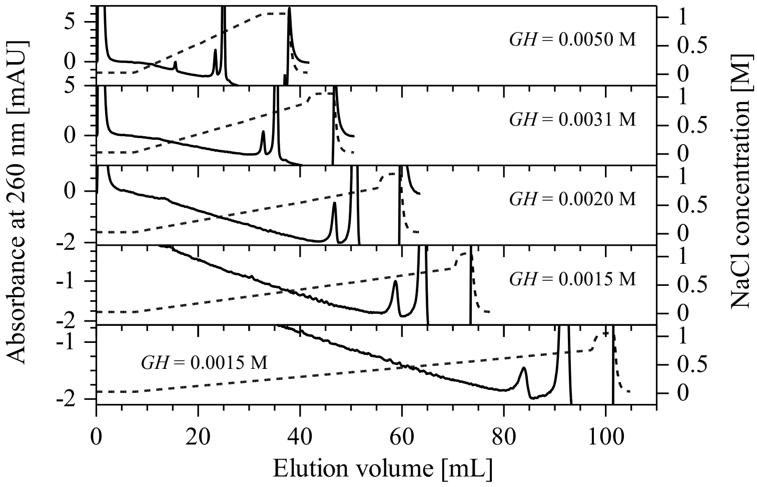

Figure S15 *continued*

(G) Elution curves of a mixture of PEGylated 90T modified with PEG10K at the 45th thymine (M-PEG10K-90T) and amino-linked 90T modified at the 45th thymine (M-NH_2-90T)

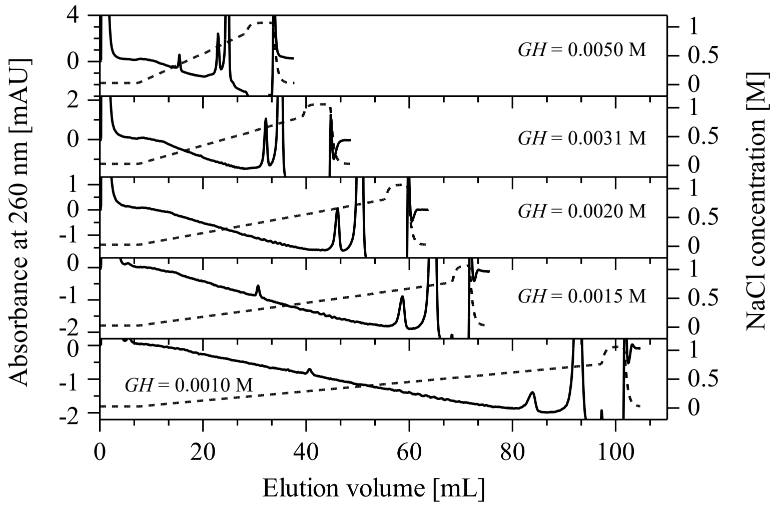

Figure S15. Elution curves of unmodified 95T and modified 95T and 95T in linear salt gradient elution using QA monolith column.

(A) 95T

(B) amino-linked 95T modified at the 5′ -end (E-NH_2-95T)

(C) a mixture of PEGylated 95T modified with PEG5K at the 5′-end (E-PEG5K-95T) and amino-linked 95T modified at the 5′ -end (E-NH_2-95T)

(D) a mixture of PEGylated 95T modified with PEG10K at the 5′ end (E-PEG10K-95T) and amino-linked 95T modified at the 5′ -end (E-NH_2-95T)

(E) amino-linked 90T modified at the 45th thymine (M-NH_2-90T)

(F) a mixture of PEGylated 90T modified with PEG5K at the 45th thymine (M-PEG5K-90T) and amino-linked 90T modified at the 45th thymine (M-NH_2-90T)

(G) a mixture of PEGylated 90T modified with PEG10K at the 45th thymine (M-PEG10K-90T) and amino-linked 90T modified at the 45th thymine (M-NH_2-90T)

The solid and dotted lines represent the absorbance at 260 nm of the column elution and the elution salt concentration, respectively. The salt concentration of the mobile phase was linearly varied from 30 mM to 1 M using a low-salt buffer (10 mM sodium phosphate buffer solution containing 30 mM NaCl, pH 7; buffer A) and a high-salt buffer (10 mM sodium phosphate buffer solution containing 1 M NaCl, pH7; buffer B). The gradient volume of salt concentration (V_g), initial and final salt concentration (I_0 and I_F), *GH*, and peak elution salt concentration (I_R) are summarized in **Table S5**.

Column A (see **Table 2**) was used. The sample injection volume was 100 μL. The sample concentrations are shown in **Table S1**. The flow rate was set to 5.0 mL/min.

Table S5 Peak elution salt concentration, I_R and model-derived parameters based on the GH-I_R model under different salt gradient volumes, V_g for unmodified and modified poly(dT) obtained from linear salt gradient elution experiments using QA monolith column (column C), as shown in **Figures. S12-S15**.

(i) unmodified and modified 9T

V_g[a] [mL]	I_0[b] [M]	I_F[c] [M]	GH[d] [M]	I_R[e] [M]	$A\times10^3$[f]	B[g]	$K_e\times10^{52}$[h]	$\|R\|$[i]	$\frac{K_e}{K_{e,\ 9T}}$[j]
**(A) 9T**									
25	0.030	0.99	0.0050	0.343 ± 0.003					
40	0.030	0.99	0.0031	0.330 ± 0.000					
63	0.030	0.99	0.0020	0.312 ± 0.001	1.3	8.1	1.2	0.998	1
84	0.030	0.99	0.0015	0.303 ± 0.000					
125	0.030	0.99	0.00099	0.290 ± 0.000					
**(B) NH_2 modified 9T at 5' -terminal (E-NH_2-9T)**									
25	0.031	0.99	0.0050	0.299 ± 0.001					
40	0.031	1.0	0.0031	0.286 ± 0.000					
63	0.031	0.99	0.0020	0.272 ± 0.000	0.38	8.1	0.35	0.997	0.30
84	0.031	1.0	0.0015	0.264 ± 0.000					
125	0.031	0.99	0.0010	0.254 ± 0.000					
**(C) PEG5K modified 9T at 5' -terminal (E-PEG5K-9T)**									
25	0.031	1.0	0.0050	0.180 ± 0.002					
40	0.031	1.0	0.0031	0.171 ± 0.001					
63	0.031	1.0	0.0020	0.163 ± 0.001	0.0039	8.1	0.0036	0.988	0.0030
84	0.031	1.0	0.0015	0.162 ± 0.000					
125	0.031	1.0	0.0010	0.157 ± 0.000					
**(D) PEG10K modified 9T at 5' -terminal (E-PEG10K-9T)**									
25	0.031	1.0	0.0050	0.168 ± 0.001					
40	0.031	1.0	0.0032	0.163 ± 0.000					
63	0.031	1.0	0.0020	0.156 ± 0.000	0.0023	8.1	0.0021	0.999	0.0018
84	0.031	1.0	0.0015	0.151 ± 0.001					
125	0.031	1.0	0.0010	0.146 ± 0.000					
**(E) NH_2 modified 9T at the forth T (M-NH_2-9T)**									
25	0.031	1.0	0.0050	0.274 ± 0.001					
40	0.031	1.0	0.0032	0.267 ± 0.000					
63	0.031	1.0	0.0020	0.252 ± 0.000	0.18	8.1	0.17	0.991	0.14
84	0.031	1.0	0.0015	0.244 ± 0.000					
125	0.031	1.0	0.0010	0.232 ± 0.000					
**(F) PEG5K modified 9T at the forth T (M-PEG5K-9T)**									
25	0.031	1.0	0.0050	0.156 ± 0.001					
40	0.031	1.0	0.0032	0.150 ± 0.000					
63	0.031	1.0	0.0020	0.141 ± 0.000	0.00095	8.1	0.00087	0.996	0.00067
84	0.031	1.0	0.0015	0.136 ± 0.000					
125	0.031	1.0	0.0010	0.130 ± 0.000					
**(G) PEG10K modified 9T at the forth T (M-PEG10K-9T)**									
25	0.031	1.0	0.0051	0.147 ± 0.002					
40	0.031	1.0	0.0032	0.142 ± 0.000					
63	0.031	1.0	0.0020	0.136 ± 0.000	0.00064	8.1	0.00059	0.997	0.00045
84	0.031	1.0	0.0015	0.132 ± 0.000					
125	0.031	1.0	0.0010	0.127 ± 0.001					

(ii) unmodified and modified 20T

V_g[a] [mL]	I_0[b] [M]	I_F[c] [M]	GH[d] [M]	I_R[e] [M]	A×10[6f]	B[g]	K_e×10[133h]	|R|[i]	$\frac{K_e}{K_{e,\ 20T}}$[j]
**(A) 20T**									
25	0.031	1.0	0.0050	0.528 ± 0.001					
40	0.031	1.0	0.0031	0.515 ± 0.001					
63	0.031	1.0	0.0020	0.505 ± 0.001	9.30	21	9.70	0.999	1
84	0.031	1.0	0.0015	0.497 ± 0.001					
125	0.031	1.0	0.0010	0.490 ± 0.001					
**(B) amino-linked 20T modified at the 5' -end (E-NH_2-20T)**									
25	0.031	1.0	0.0050	0.490 ± 0.001					
40	0.031	1.0	0.0031	0.477 ± 0.000					
63	0.031	1.0	0.0020	0.468 ± 0.001	1.70	21	1.77	0.999	0.190
84	0.031	1.0	0.0015	0.459 ± 0.000					
125	0.031	1.0	0.0010	0.449 ± 0.000					
**(C) PEGylated 20T modified with PEG5K at the 5' -end (E-PEG5K-20T)**									
25	0.031	1.0	0.0050	0.377 ± 0.001					
40	0.031	1.0	0.0031	0.367 ± 0.000					
63	0.031	1.0	0.0020	0.360 ± 0.001	5.9×10^{-3}	21	6.2×10^{-3}	0.997	0.00067
84	0.031	1.0	0.0015	0.352 ± 0.002					
125	0.031	1.0	0.0010	0.345 ± 0.001					
**(D) PEGylated 20T modified with PEG10K at the 5' -end (E-PEG10K-20T)**									
25	0.031	1.0	0.0050	0.357 ± 0.000					
40	0.031	1.0	0.0031	0.355 ± 0.006					
63	0.031	1.0	0.0020	0.342 ± 0.000	2.2×10^{-3}	21	2.3×10^{-3}	0.978	0.00025
84	0.031	1.0	0.0015	0.337 ± 0.000					
125	0.031	1.0	0.0010	0.330 ± 0.000					
**(E) amino-linked 20T modified at the 10th thymine (M-NH_2-20T)**									
25	0.031	1.0	0.0050	0.487 ± 0.001					
40	0.031	1.0	0.0031	0.476 ± 0.002					
63	0.031	1.0	0.0020	0.467 ± 0.003	1.69	21	1.8	0.996	0.194
84	0.031	1.0	0.0015	0.462 ± 0.003					
125	0.031	1.0	0.0010	0.450 ± 0.003					
**(F) PEGylated 20T modified with PEG5K at the 10th thymine (M-PEG5K-20T)**									
25	0.031	1.0	0.0050	0.356 ± 0.001					
40	0.031	1.0	0.0031	0.347 ± 0.000					
63	0.031	1.0	0.0020	0.340 ± 0.001	1.8×10^{-3}	21	1.9×10^{-3}	0.999	0.00020
84	0.031	1.0	0.0015	0.335 ± 0.001					
125	0.031	1.0	0.0010	0.327 ± 0.000					
**(G) PEGylated 20T modified with PEG10K at the 10th thymine (M-PEG10K-20T)**									
25	0.031	1.0	0.0050	0.341 ± 0.001					
40	0.031	1.0	0.0031	0.335 ± 0.001					
63	0.031	1.0	0.0020	0.327 ± 0.000	8.3×10^{-4}	21	8.6×10^{-4}	0.993	0.000092
84	0.031	1.0	0.0015	0.323 ± 0.002					
125	0.031	1.0	0.0010	0.320 ± 0.001					

(iii) unmodified and modified 50T

V_g[a] [mL]	I_0[b] [M]	I_F[c] [M]	GH[d] [M]	I_R[e] [M]	$A\times10^{6}$[f]	B[g]	$K_e\times10^{244}$[h]	$\lvert R\rvert$[i]	$\frac{K_e}{K_{e,\ 50T}}$[j]
**(A) 50T**									
25	0.031	0.99	0.0050	0.670 ± 0.005					
40	0.031	1.0	0.0031	0.663 ± 0.003					
63	0.031	1.0	0.0020	0.659 ± 0.000	1.39	37	2.61	0.981	1
84	0.031	1.0	0.0015	0.649 ± 0.000					
125	0.031	1.0	0.0010	0.644 ± 0.001					
**(B) amino-linked poly 50T at the 5’ -end (E-NH_2-50T)**									
25	0.031	1.0	0.0050	0.639 ± 0.001					
40	0.031	1.0	0.0031	0.634 ± 0.000					
63	0.031	1.0	0.0020	0.628 ± 0.000	0.237	37	0.908	0.982	0.17
84	0.031	1.0	0.0015	0.622 ± 0.000					
125	0.031	1.0	0.0010	0.613 ± 0.005					
**(C) PEGylated 50T modified with PEG5K at the 5’ -end (E-PEG5K-50T)**									
25	0.031	1.0	0.0050	0.553 ± 0.000					
40	0.031	1.0	0.0031	0.549 ± 0.001					
63	0.031	1.0	0.0020	0.546 ± 0.000	1.11×10^{-3}	37	0.00424	0.995	0.00079
84	0.031	1.0	0.0015	0.541 ± 0.000					
125	0.031	1.0	0.0010	0.535 ± 0.001					
**(D) PEGylated 50T modified with PEG10K at the 5’ -end (E-PEG10K-50T)**									
25	0.031	1.0	0.0050	0.552 ± 0.001					
40	0.031	1.0	0.0031	0.546 ± 0.000					
63	0.031	1.0	0.0020	0.541 ± 0.000	8.50×10^{-4}	37	0.00325	0.984	0.00061
84	0.031	1.0	0.0015	0.536 ± 0.001					
125	0.031	1.0	0.0010	0.530 ± 0.000					
**(E) amino-linked 50T modified at the 24th thymine (M-NH2-50T)**									
25	0.031	1.0	0.0050	0.629 ± 0.005					
40	0.031	1.0	0.0032	0.625 ± 0.002					
63	0.031	1.0	0.0020	0.619 ± 0.002	0.183	37	0.701	0.822	0.13
84	0.031	1.0	0.0015	0.625 ± 0.001					
125	0.031	1.0	0.0010	0.618 ± 0.003					
**(F) PEGylated 50T modified with PEG5K at the 24th thymine (M-PEG5K-50T)**									
25	0.031	1.0	0.0050	0.549 ± 0.002					
40	0.031	1.0	0.0032	0.542 ± 0.001					
63	0.031	1.0	0.0020	0.538 ± 0.001	6.83×10^{-4}	37	0.00261	0.988	0.00049
84	0.031	1.0	0.0015	0.534 ± 0.002					
125	0.031	1.0	0.0010	0.527 ± 0.001					
**(G) PEGylated 50T modified with PEG10K at the 24th thymine (M-PEG10K-50T)**									
25	0.031	1.0	0.0050	0.540 ± 0.003					
40	0.031	1.0	0.0032	0.535 ± 0.000					
63	0.031	1.0	0.0020	0.529 ± 0.000	3.89×10^{-4}	37	0.00148	0.999	0.00028
84	0.031	1.0	0.0015	0.524 ± 0.000					
125	0.031	1.0	0.0010	0.523 ± 0.001					

(iv) unmodified and modified 90T or 95T

V_g[a] [mL]	I_0[b] [M]	I_F[c] [M]	GH[d] [M]	I_R[e] [M]	A×10^9[f]	B[g]	K_e[h]	|R|[i]	$\frac{K_e}{K_{e,\,95T}}$[j]
**(A) 95T**									
25	0.031	1.0	0.0050	0.725 ± 0.002					
40	0.031	1.0	0.0031	0.719 ± 0.000					
63	0.031	1.0	0.0020	0.716 ± 0.001	2.98	64	10^{421}	0.999	1
84	0.031	1.0	0.0015	0.712 ± 0.000					
125	0.031	1.0	0.0010	0.706 ± 0.002					
**(B) amino-linked 95T modified at the 5'-end (E-NH2-95T)**									
25	0.031	1.0	0.0050	0.699 ± 0.002					
40	0.031	1.0	0.0031	0.701 ± 0.003					
63	0.031	1.0	0.0020	0.695 ± 0.002	0.501	64	10^{421}	0.858	
84	0.031	1.0	0.0015	0.695 ± 0.002					
125	0.031	1.0	0.0010	0.691 ± 0.002					
**(C) PEGylated 95T modified with PEG5K at the 5'-end (E-PEG5K-95T)**									
25	0.030	1.0	0.0051	N.D.[k]					
40	0.031	1.0	0.0032	N.D.[k]					
63	0.031	1.0	0.0020	N.D.[k]	N.A.[k]	N.A.[k]	N.A.[k]	N.A.[k]	N.A.[k]
84	0.031	1.0	0.0015	N.D.[k]					
125	0.030	1.0	0.0010	N.D.[k]					
**(D) PEGylated 95T modified with PEG10K at the 5' end (E-PEG10K-95T)**									
25	0.031	1.0	0.0050	N.D.[k]					
40	0.031	1.0	0.0032	N.D. [k]					
63	0.031	1.0	0.0020	N.D.[k]	N.A.[k]	N.A.[k]	N.A.[k]	N.A.[k]	N.A.[k]
84	0.031	1.0	0.0015	N.D.[k]					
125	0.031	1.0	0.0010	N.D.[k]					
**(E) amino-linked 90T modified at the 45th thymine (M-NH2-90T)**									
25	0.031	1.0	0.0050	0.700 ± 0.001					
40	0.031	1.0	0.0032	0.695 ± 0.001					
63	0.031	1.0	0.0020	0.690 ± 0.003	0.231	64	10^{420}	0.266	0.078
84	0.031	1.0	0.0015	0.680 ± 0.000					
125	0.031	1.0	0.0010	0.675 ± 0.002					
**(F) PEGylated 90T modified with PEG5K at the 45th thymine (M-PEG5K-90T)**									
25	0.031	1.0	0.0050	0.637 ± 0.003					
40	0.031	1.0	0.0031	0.638 ± 0.002					
63	0.031	1.0	0.0020	0.630 ± 0.000	0.0065	64	10^{418}	0.938	0.00022
84	0.031	1.0	0.0015	0.621 ± 0.001					
125	0.031	1.0	0.0010	0.615 ± 0.002					
**(G) PEGylated 90T modified with PEG10K at the 45th thymine (M-PEG10K-90T)**									
25	0.031	1.0	0.0050	0.629 ± 0.000					
40	0.031	1.0	0.0031	0.627 ± 0.000					
63	0.031	1.0	0.0020	0.623 ± 0.003	0.0047	64	10^{418}	0.958	0.0016
84	0.031	1.0	0.0015	0.621 ± 0.003					
125	0.031	1.0	0.0010	0.621 ± 0.000					

[a]Vg: Gradient volume from the initial salt concentration (I_0) to the final salt concentration (I_f) in a linear salt gradient elution experiment.

[b]I_0: Initial salt concentration.

[c]I_f: Final salt concentration.

[d]GH: Normalized gradient slope, calculated as $GH = \frac{I_f - I_0}{V_g / V_t(1-\varepsilon)}$, where V_t (= 1.0 mL) is the total column volume and V_s (= 0.71 mL) is the stationary phase volume (i. e. $V_t(1-\varepsilon)$).

[e]I_R: Peak elution salt concentration.

[f]A: Fitting parameter of the GH-IR model described by **Equation (2)**.

[g]B: Number of the binding sites of the solute to the stationary phase.

[h]K_e: Ion exchange reaction constant.

[i]$|R|$: Correlation coefficient based on **Equation (2)**.

[j]$\frac{K_e}{K_{e,\,ref}}$: Ratio of ion-exchange reaction constant of modified poly(dT) to that of corresponding unmodified reference (e.g., 9T, 20T, 50T, 90T, or 95T).

[k]Due to the low yield of PEGylation, a distinct peak corresponding to PEGylated 95T was not observed.

6. Calculated elution salt concentration of PEGylated and unreacted poly(dT) in linear salt gradient.

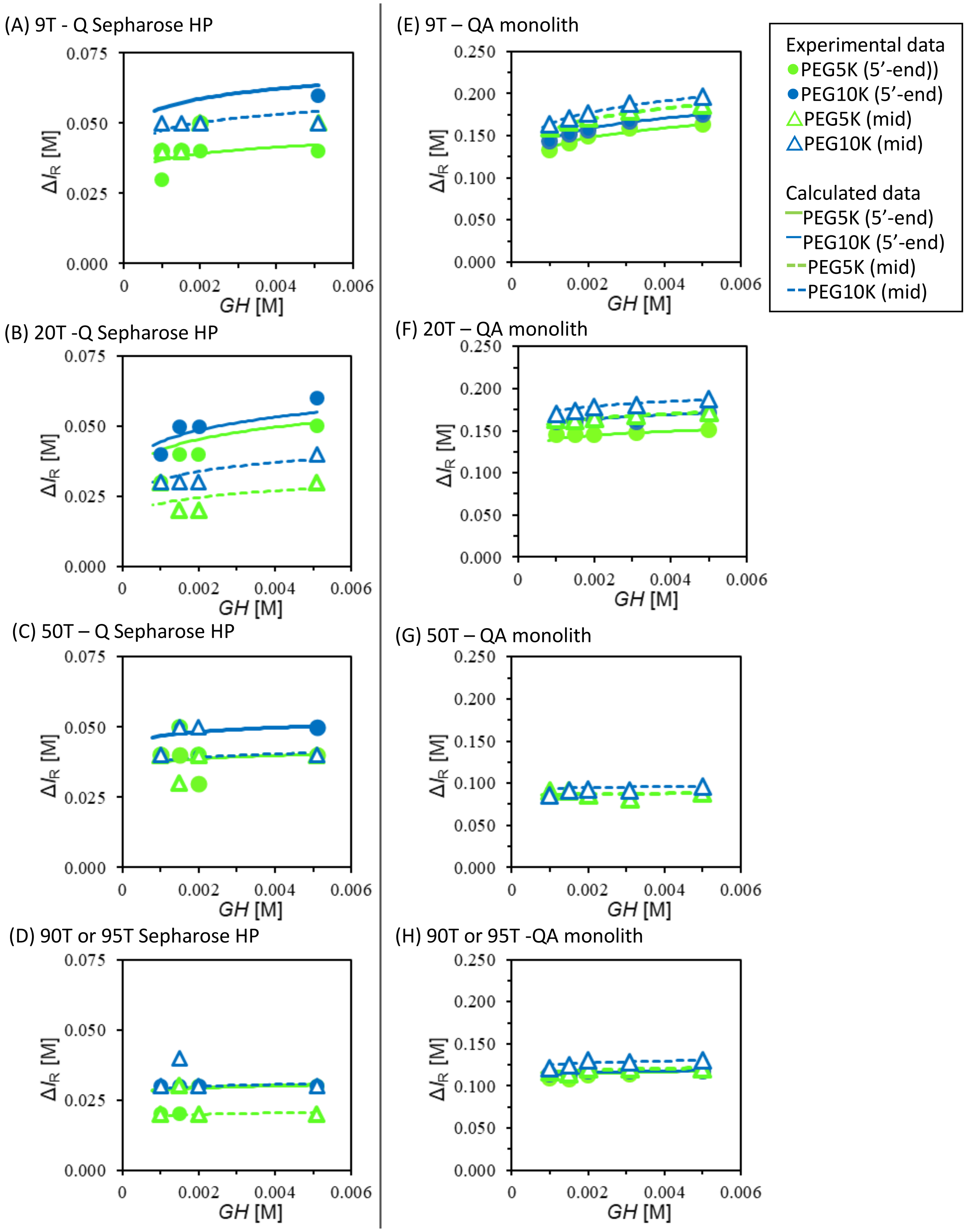

Figure S16. Differences in elution salt concentration, ΔI_R between PEGylated and unreacted poly(dT) in linear salt gradient.

ΔI_R values were obtained using (A - D) Q Sepharose HP column and (E - H) QA monolith column.

(A, E) 9T, (B, F) 20T, (C, G) 50T, (D, H) 90T or 95T.

Green filled circle: Experimental ΔI_R between PEGylated poly(dT) with PEG5K at the 5' -end (E-PEG10K-T) and unreacted amino-linked poly(dT)

Blue filled circle: Experimental ΔI_R t between PEGylated poly(dT) with PEG10K at the 5' -end (E-PEG10K-T) and unreacted amino-linked poly(dT)

Green open triangle: Experimental ΔI_R between PEGylated poly(dT) with PEG5K at the mid position (E-PEG10K-T)

Blue open triangle: Experimental ΔI_R obtained between PEGylated poly(dT) with PEG10K at the mid position(E-PEG10K-T) and unreacted amino-linked poly(dT)

Green solid line: Calculated ΔI_R obtained by calculation using **equation (9)** for PEGylated poly(dT) with PEG5K at the 5' -end (E-PEG5K-T)

Blue solid line: Calculated ΔI_R using **equation (9)** between PEGylated poly(dT) with PEG10K at the 5' -end (E-PEG10K-T)

Green dotted line: Calculated ΔI_R using **equation (9)** PEGylated poly(dT) with PEG5K at the mid position (E-PEG5K-T)

Blue dotted line: Calculated ΔI_R using **equation (9)** PEGylated poly(dT) with PEG10K at the mid position(E-PEG10K-T)

7. Data used to determine mass transfer properties of PEGylated oligonucleotide.

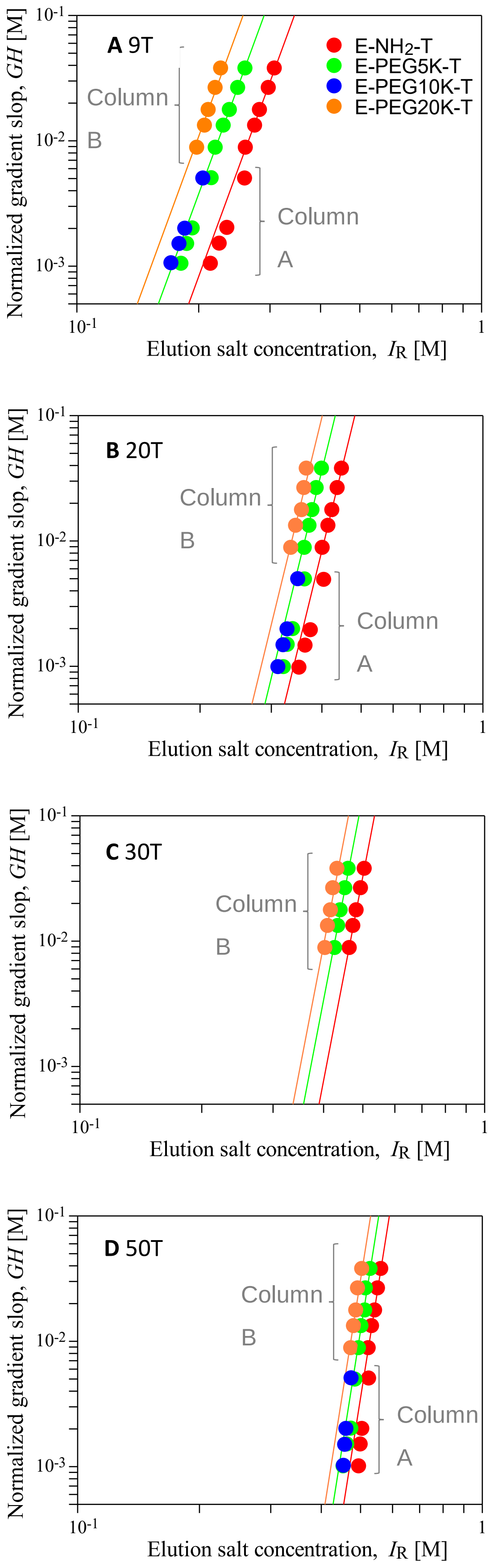

Figure S17. Comparison of *GH*-I_R plots for reaction mixtures of PEGylated and unreacted amino-linked poly(dT) with different base lengths (9T-50T), modified with PEG5k, 10K and 20K, analyzed using prepacked and self-packed Q Sepharose columns (Column A and Column B).

(A) Reaction mixture of PEGylated and unreacted 9T

(B) Reaction mixture of PEGylated and unreacted 20T

(C) Reaction mixture of PEGylated and unreacted 30T

(D) Reaction mixture of PEGylated and unreacted 50T

Red circles: amino-linked poly(dT) at the 5’ -end ($E\text{-}NH_2\text{-}T$)

Green circles: PEGylated poly(dT) with PEG5K at the 5’ -end (E-PEG5K-T)

Blue circles: PEGylated poly(dT) with PEG10K at the 5’ -end (E-PEG10K-T)

Orange circles: PEGylated poly(dT) with PEG20K at the 5’ -end (E-PEG20K-T)

A Elution curves of a mixture of PEGylated 9T modified with PEG5K at the 5′-end (E-PEG5K-9T) and amino-linked 9T modified at the 5′ -end (E-NH_2-9T)

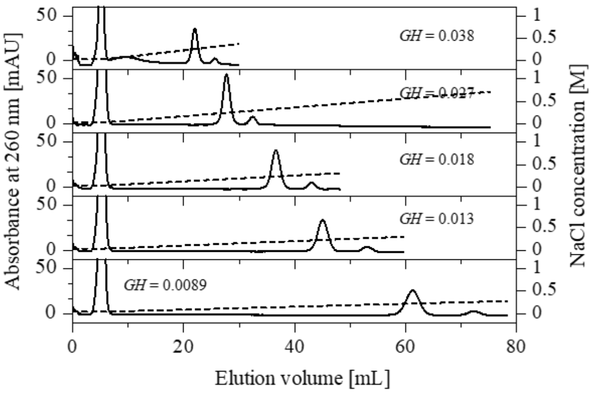

B Elution curves of a mixture of PEGylated 9T modified with PEG20K at the 5′ -end (E-PEG20K-9T) and unreacted amino-linked 9T modified at the 5′ -end (E-NH_2-9T)

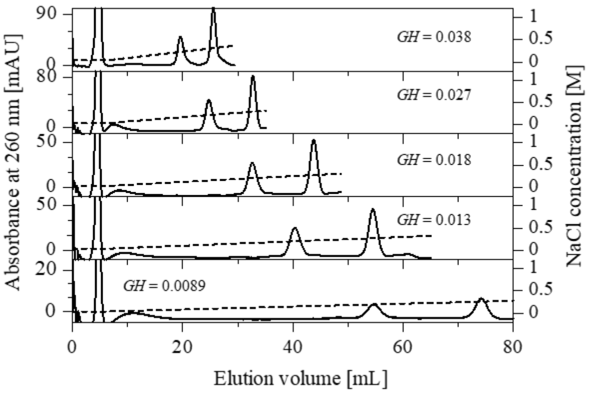

C Elution curves of a mixture of PEGylated 20T modified with PEG5K at the 5′ -end (E-PEG5K-20T) and unreacted amino-linked 20T modified at the 5′ -end (E-NH_2-20T)

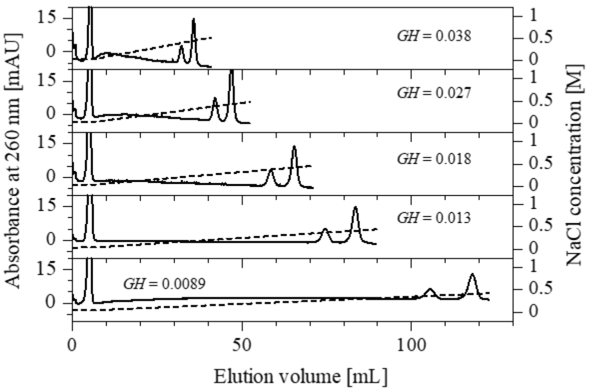

Figure S18 *continued*

D Elution curves of a mixture of PEGylated 20T modified with PEG20K at the 5′ -end (E-PEG20K-20T) and unreacted amino-linked 20T modified at the 5′ -end (E-NH_2-20T)

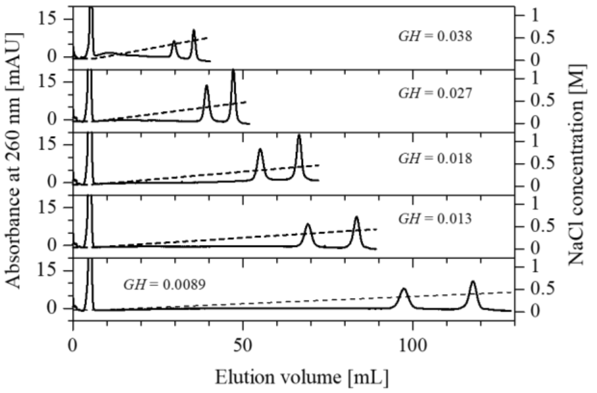

E Elution curves of a mixture of PEGylated 30T modified with PEG5K at the 5′ -end (E-PEG5K-30T) and unreacted amino-linked 30T modified at the 5′ -end (E-NH_2-30T)

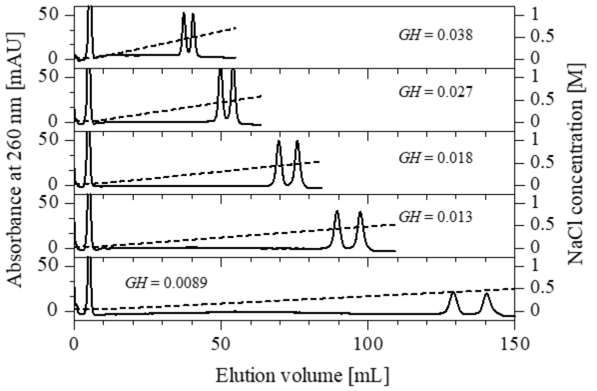

F Elution curves of a mixture of PEGylated 30T modified with PEG20K at the 5′ -end (E-PEG20K-30T) and unreacted amino-linked 30T modified at the 5′ -end (E-NH_2-30T)

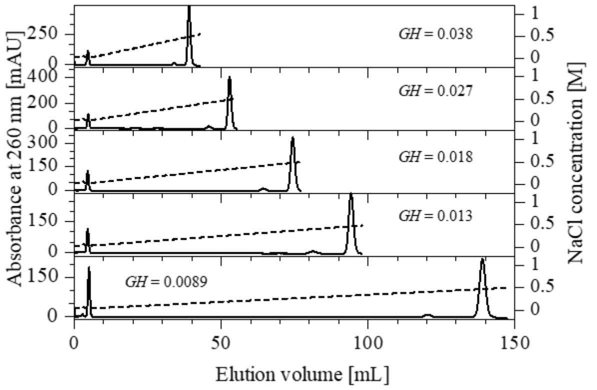

Figure S18 *continued*

G Elution curves of a mixture of PEGylated 50T modified with PEG5K at the 5′ -end (E-PEG5K-50T) and unreacted amino-linked 50T modified at the 5′ -end (E-NH_2-50T)

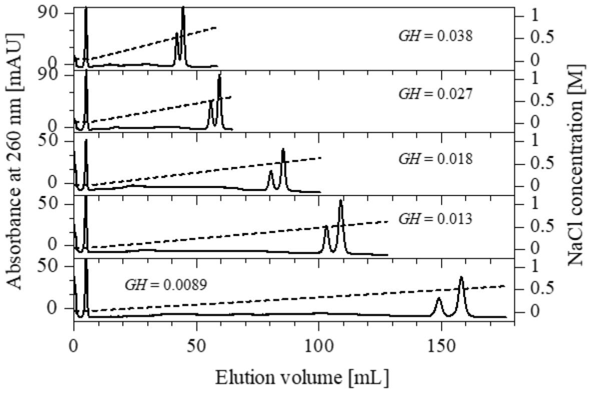

H Elution curves of a mixture of PEGylated 50T modified with PEG20K at the 5′ -end (E-PEG20K-50T) and unreacted amino-linked 50T modified at the 5′ -end (E-NH_2-50T)

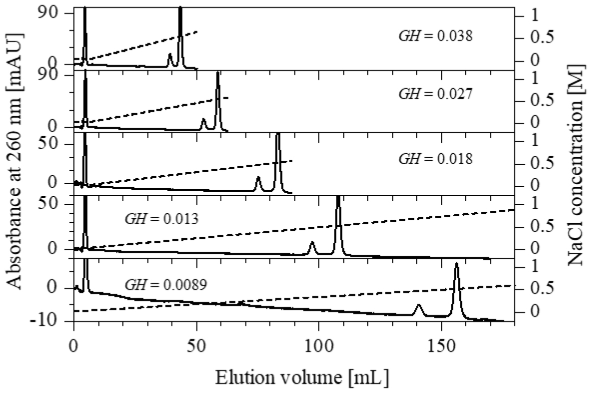

Figure S18. Elution curves of a mixture of PEGylated and unreacted amino-linked poly(dT) at the 5′ -end in linear salt gradient elution using Q Sepharose HP column (Column B) for determination of height of equivalent theoretical plate, $HETP_{LGE}$.

(A) a mixture of PEGylated 9T modified with PEG5K at the 5′ -end (E-PEG5K-9T) and amino-linked 9T modified at the 5′- end (E-NH_2-9T)

(B) a mixture of PEGylated 9T modified with PEG20K at the 5′ -end (E-PEG20K-9T) and amino-linked 9T modified at the 5′ -end (E-NH_2-9T)

(C) a mixture of PEGylated 20T modified with PEG5K at the 5′ -end (E-PEG5K-20T) and amino-linked 20T modified at the 5′ -end (E-NH_2-20T)

(D) a mixture of PEGylated 20T modified with PEG20K at the 5′ -end (E-PEG20K-20T) and amino-linked 20T modified at the 5′-end (E-NH_2-20T)

(E) a mixture of PEGylated 30T modified with PEG5K at the 5′ -end (E-PEG5K-30T) and amino-linked 30T modified at the 5′ -end (E-NH_2-30T)

(F) a mixture of PEGylated 30T modified with PEG20K at the 5′ -end (E-PEG20K-30T) and amino-linked 30T modified at the 5′ -end (E-NH_2-30T)

(G) a mixture of PEGylated 30T modified with PEG5K at the 5′ -end (E-PEG5K-50T) and amino-linked 50T modified at the 5′ -end (E-NH_2-50T)

(H) a mixture of PEGylated 50T modified with PEG20K at the 5’ -end (E-PEG20K-50T) and amino-linked 50T modified at the 5’ -end (E-NH_2-50T)

The solid and dotted lines represent the absorbance at 260 nm of the column elution and the elution salt concentration, respectively. The salt concentration of the mobile phase was linearly varied from 30 mM to 1 M using a low-salt buffer (10 mM sodium phosphate buffer solution containing 30 mM NaCl, pH 7; buffer A) and a high-salt buffer (10 mM sodium phosphate buffer solution containing 1 M NaCl, pH7; buffer B). The gradient volume of salt concentration (V_g), initial and final salt concentration (I_0 and I_F), *GH*, and peak elution salt concentration (I_R) are summarized in **Table S6**.

Column A (see **Table 2**) was used. The sample injection volume was 100 μL. The sample concentrations are shown in **Table S1**. The flow rate was set to 1.0 mL/min.

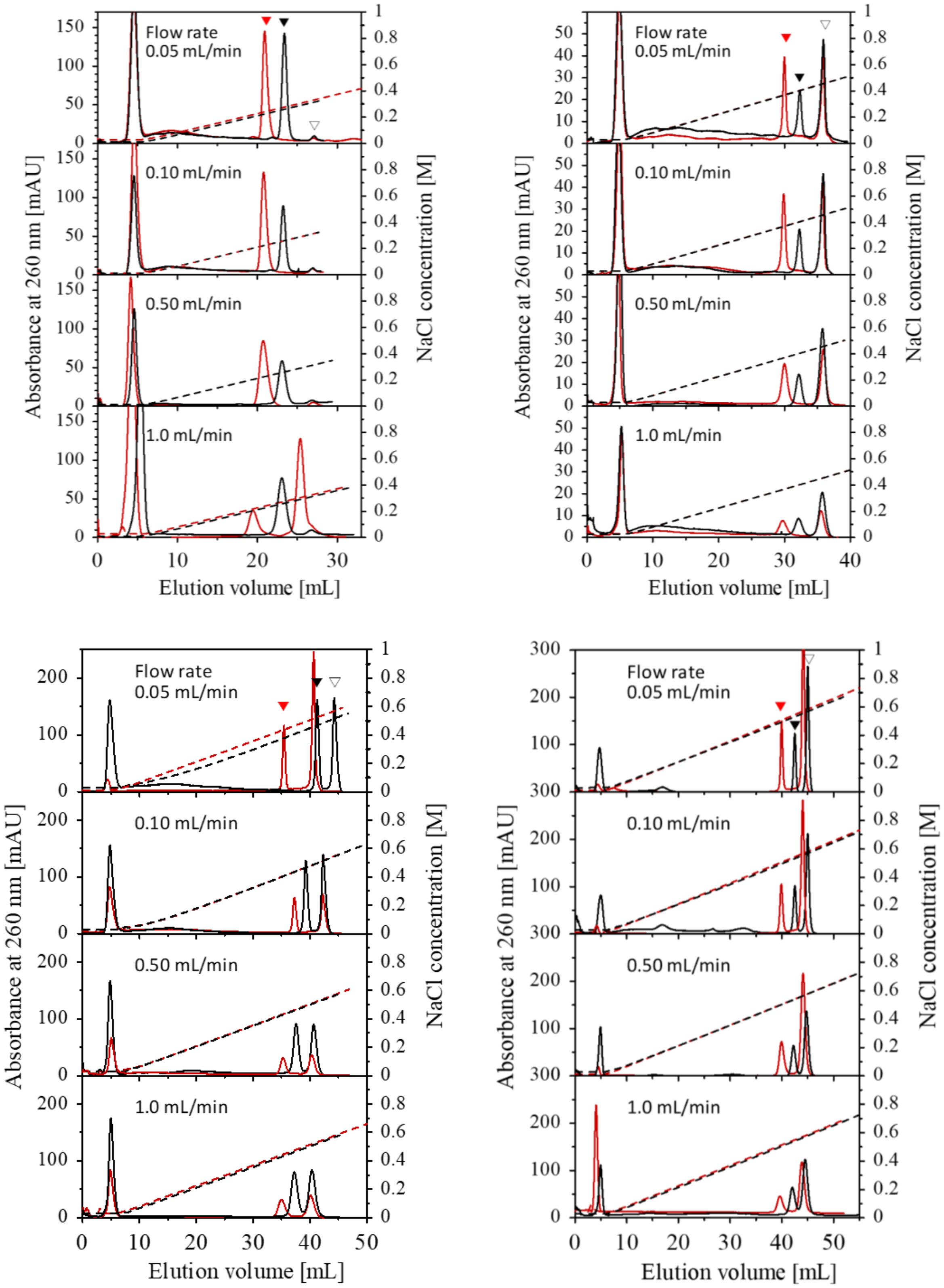

Figure S19. Elution curves of a mixture of PEGylated and unreacted amino-linked poly(dT) at the 5′ -end in linear salt gradient elution using Q Sepharose HP column (column B) at various flow rates f for determining of the height equivalent to a theoretical plate, $HETP_{LGE}$.

(A) a mixture of PEGylated 9T modified with PEG5K or PEG20K at the 5′ -end (E-PEG5K-9T or E-PEG20K-9T) and amino-linked 9T modified at the 5′-end(E-NH_2-9T)
(B) a mixture of PEGylated 20T modified with PEG5K or PEG20K at the 5′ -end (E-PEG5K-20T or E-PEG20K-20T) and amino-linked 20T modified at the 5′-end(E-NH_2-20T)
(C) a mixture of PEGylated 30T modified with PEG5K or PEG20K at the 5′ -end (E-PEG5K-30T or E-PEG20K-30T) and amino-linked 30T modified at the 5′-end(E-NH_2-30T)
(D) a mixture of PEGylated 50T modified with PEG5K or PEG20K at the 5′ -end (E-PEG5K-50T or E-PEG20K-50T) and amino-linked 50T modified at the 5′-end(E-NH_2-50T)

The solid and dotted lines represent the absorbance at 260 nm of the column elution and the elution salt concentration, respectively.

Black lines: Mixture containing PEGylated poly(dT) with PEG50K

Red lines: Mixture of PEGylated poly(dT) with PEG20K

Open black triangle: The peak of unreacted poly(dT)

Solid black triangle: The peak of PEGylated poly(dT) with PEG5K

Solid red triangle: The peak of PEGylated poly(dT) with PEG20K

The salt concentration of the mobile phase was linearly varied from 30 mM to 1 M using a low-salt buffer (10 mM sodium phosphate buffer solution containing 30 mM NaCl, pH 7; buffer A) and a high-salt buffer (10 mM sodium phosphate buffer solution containing 1 M NaCl, pH7; buffer B). The gradient volume of salt concentration (V_g) was set to 70 mL. The initial and final salt concentration (I_0 and I_F), *GH*, and peak elution salt concentration (I_R) are summarized in **Table S6**.

Column B (see **Table 2**) was used. The sample injection volume was 100 μL. The sample concentrations are shown in **Table S1**. Flow rate was set to 0.05 - 1.0 mL/min.

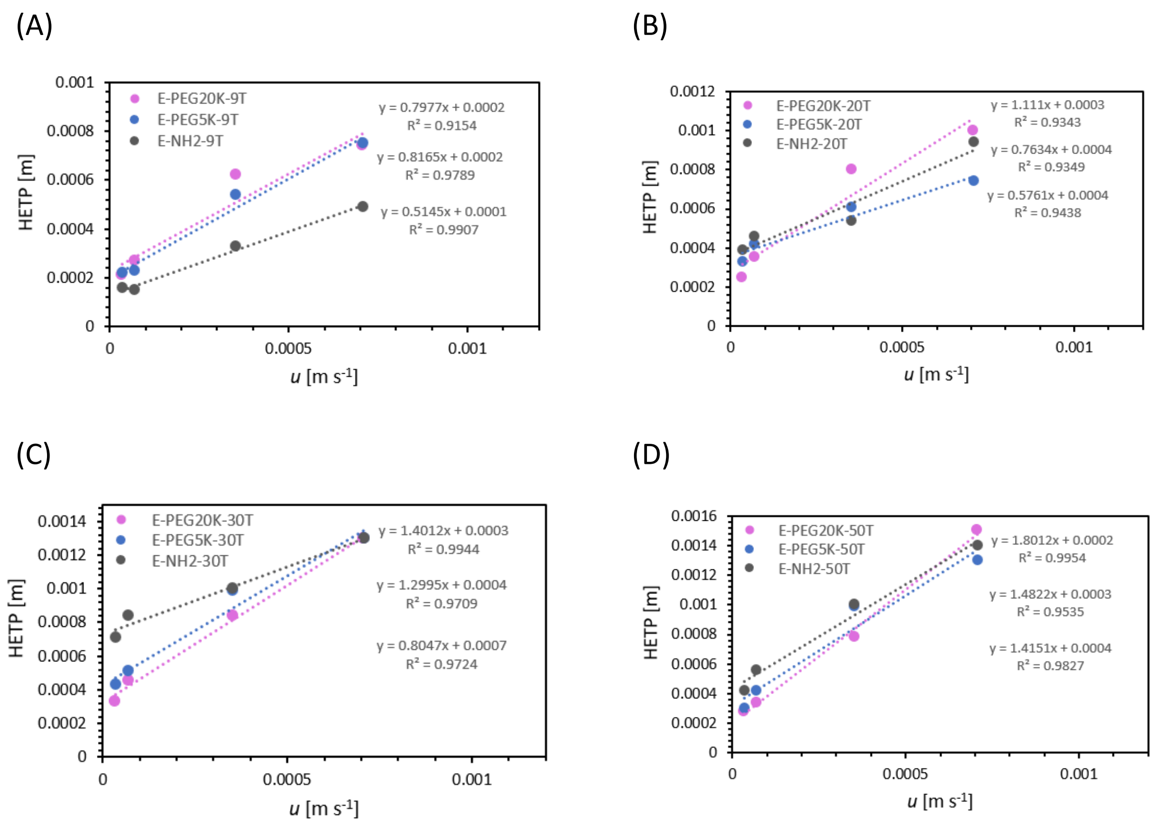

Figure S20. van Deemter plots of PEGylated and unreacted amino-linked poly(dT) at the 5' -end in linear salt gradient elution using Q Sepharose HP column (Column B) at various flow rates for determining of the height equivalent to a theoretical plate, $HETP_{LGE}$.

(A) PEGylated 9T modified with PEG5K or PEG20K at the 5' -end (E-PEG5K-9T or E-PEG20K-9T) and amino-linked 9T modified at the 5'-end(E-NH_2-9T)

(B) PEGylated 20T modified with PEG5K or PEG20K at the 5' -end (E-PEG5K-20T or E-PEG20K-20T) and amino-linked 20T modified at the 5'-end(E-NH_2-20T)

(C) PEGylated 30T modified with PEG5K or PEG20K at the 5' -end (E-PEG5K-30T or E-PEG20K-30T) and amino-linked 30T modified at the 5'-end(E-NH_2-30T)

(D) PEGylated 50T modified with PEG5K or PEG20K at the 5' -end (E-PEG5K-50T or E-PEG20K-50T) and amino-linked 50T modified at the 5'-end(E-NH_2-50T)

Black circle: Unreacted poly(dT)

Blue circle: PEGylated poly(dT) with PEG5K

Pink circle: PEGylated poly(dT) with PEG20K

$HETP_{LGE}$ was calculated using **Equation (5)** with the peak data shown in **Figure S19** and summarized in **Table S6**.

Table S6 Peak elution salt concentration, I_R, model-derived parameters, peak standard deviation, σ and height equivalent to a theoretical plate, $HETP_{LGE}$ based on the *GH*-I_R model under different salt gradient volumes, V_g for unmodified and modified poly(dT) obtained from linear salt gradient elution experiments using Q Sepharose column (column B), as shown in **Figures. S18** and **19**.

(i) unmodified and modified 9T

V_g^a [mL]	GH^b [M]	F^c [mL/min]	I_R^d [M]	σ_V^e [mL]	K_R^f	M^g	L^h	V_R^i [mL]	$HETP_{LGE}^j$ [cm]	$D_P^k \times 10^{11}$ [m²/s]
**(A) NH2 modified 9T at 5'-terminal (E-NH2-9T)** $A^l = 0.53 \times 10^{-3}$, $B^m = 6.5$, $\lvert R^n \rvert = 0.999$, $D_0^o = 9.1 \times 10^{-11}$ m²/s										
70	0.038	0.05	0.313 ± 0.002	0.26 ± 0.03	1.6 ± 0.0	1.2 ± 0.0	0.81 ± 0.00	5.6 ± 0.1	0.016 ± 0.003	1.2
		0.10	0.314 ± 0.002	0.25 ± 0.03	1.6 ± 0.0	1.2 ± 0.0	0.81 ± 0.00	5.6 ± 0.1	0.015 ± 0.002	
		0.50	0.313 ± 0.001	0.37 ± 0.01	1.6 ± 0.0	1.2 ± 0.0	0.81 ± 0.00	5.7 ± 0.1	0.033 ± 0.002	
		1.0	0.312 ± 0.001	0.46 ± 0.01	1.6 ± 0.0	1.2 ± 0.0	0.81 ± 0.00	5.7 ± 0.1	0.049 ± 0.000	
100	0.027	1.0	0.297 ± 0.001	0.54 ± 0.01	2.1 ± 0.0	1.4 ± 0.0	0.84 ± 0.00	6.8 ± 0.1	0.043 ± 0.000	N.D.^{p}
150	0.018	1.0	0.281 ± 0.001	0.67 ± 0.03	2.7 ± 0.0	1.8 ± 0.0	0.87 ± 0.00	8.6 ± 0.1	0.040 ± 0.005	N.D.^{p}
200	0.013	1.0	0.272 ± 0.002	0.74 ± 0.01	3.3 ± 0.2	2.1 ± 0.1	0.89 ± 0.01	10 ± 0.4	0.034 ± 0.003	N.D.^{p}
300	0.0089	1.0	0.257 ± 0.002	0.94 ± 0.01	4.4 ± 0.2	2.8 ± 0.1	0.92 ± 0.00	13 ± 0.5	0.030 ± 0.003	N.D.^{p}
**(B) PEG5K modified 9T at 5'-terminal (E-PEG5K-9T)** $A^l = 0.15 \times 10^{-3}$, $B^m = 6.5$, $\lvert R^n \rvert = 0.999$, $D_0^o = 6.1 \times 10^{-11}$ m²/s										
70	0.038	0.05	0.262 ± 0.001	0.29 ± 0.01	1.5 ± 0.0	1.1 ± 0.0	0.80 ± 0.00	5.4 ± 0.1	0.022 ± 0.001	0.8
		0.10	0.263 ± 0.001	0.29 ± 0.01	1.5 ± 0.0	1.1 ± 0.0	0.80 ± 0.00	5.4 ± 0.0	0.023 ± 0.001	
		0.50	0.261 ± 0.000	0.46 ± 0.00	1.6 ± 0.0	1.1 ± 0.0	0.80 ± 0.00	5.5 ± 0.0	0.054 ± 0.001	
		1.0	0.261 ± 0.001	0.54 ± 0.00	1.6 ± 0.0	1.1 ± 0.0	0.81 ± 0.00	5.5 ± 0.0	0.075 ± 0.001	
100	0.027	1.0	0.250 ± 0.000	0.80 ± 0.00	1.9 ± 0.0	1.3 ± 0.0	0.83 ± 0.00	6.4 ± 0.0	0.077 ± 0.000	N.D.^{p}
150	0.018	1.0	0.238 ± 0.001	0.91 ± 0.00	2.4 ± 0.1	1.6 ± 0.1	0.86 ± 0.00	7.7 ± 0.0	0.074 ± 0.000	N.D.^{p}
200	0.013	1.0	0.232 ± 0.001	1.1 ± 0.0	2.7 ± 0.1	1.8 ± 0.0	0.88 ± 0.00	8.7 ± 0.3	0.071 ± 0.005	N.D.^{p}
300	0.0089	1.0	0.222 ± 0.001	1.1 ± 0.0	3.6 ± 0.1	2.3 ± 0.0	0.90 ± 0.00	11 ± 0.2	0.062 ± 0.001	N.D.^{p}
**(C) PEG20K modified 9T at 5'-terminal (E-PEG20K-9T)** $A^l = 0.059$, $B^m = 6.5$, $\lvert R^n \rvert = 1$, $D_0^o = 2.9 \times 10^{-11}$ m²/s										
70	0.038	0.05	0.228 ± 0.001	0.27 ± 0.05	1.5 ± 0.0	1.1 ± 0.0	0.80 ± 0.00	5.3 ± 0.0	0.021 ± 0.008	0.8
		0.10	0.229 ± 0.000	0.31 ± 0.04	1.5 ± 0.0	1.1 ± 0.0	0.80 ± 0.00	5.2 ± 0.0	0.027 ± 0.007	
		0.50	0.227 ± 0.001	0.47 ± 0.07	1.6 ± 0.0	1.1 ± 0.0	0.80 ± 0.00	5.4 ± 0.1	0.062 ± 0.021	
		1.0	0.226 ± 0.001	0.53 ± 0.04	1.6 ± 0.0	1.1 ± 0.0	0.80 ± 0.00	5.5 ± 0.1	0.074 ± 0.013	
100	0.027	1.0	0.220 ± 0.000	0.69 ± 0.11	1.8 ± 0.0	1.3 ± 0.0	0.82 ± 0.00	6.0 ± 0.0	0.10 ± 0.03	N.D.^{p}
150	0.018	1.0	0.211 ± 0.001	0.84 ± 0.09	2.1 ± 0.0	1.5 ± 0.0	0.85 ± 0.00	7.0 ± 0.1	0.10 ± 0.02	N.D.^{p}
200	0.013	1.0	0.205 ± 0.002	0.93 ± 0.11	2.4 ± 0.1	1.6 ± 0.1	0.86 ± 0.01	7.8 ± 0.3	0.095 ± 0.01	N.D.^{p}
300	0.0089	1.0	0.197 ± 0.001	1.2 ± 0.08	3.0 ± 0.1	2.0 ± 0.0	0.88 ± 0.00	9.4 ± 0.2	0.10 ± 0.01	N.D.^{p}

(ii) unmodified and modified 20T

V_g[a] [mL]	GH[b] [M]	F[c] [mL/min]	I_R[d] [M]	σ_v[e] [mL]	K_R[f]	M[g]	L[h]	V_R[i] [mL]	$HETP_{LGE}$[j] [cm]	D_p[k] × 10^{11} [m²/s]
**(A) NH_2 modified 20T at 5'-terminal (E-NH_2-20T)** A[l] = 0.073 × 10^{-3}, B[m] = 12, |R[n]| = 0.994, D_0[o] = 6.1 × 10^{-11} m²/s										
70	0.038	0.05	0.454 ± 0.003	0.34 ± 0.05	1.4 ± 0.1	1.0 ± 0.0	0.78 ± 0.01	4.9 ± 0.2	0.039 ± 0.007	1.2
		0.10	0.456 ± 0.002	0.37 ± 0.00	1.4 ± 0.0	1.0 ± 0.0	0.78 ± 0.01	4.9 ± 0.1	0.046 ± 0.003	
		0.50	0.451 ± 0.001	0.43 ± 0.01	1.5 ± 0.0	1.1 ± 0.0	0.79 ± 0.00	5.2 ± 0.0	0.054 ± 0.002	
		1.0	0.452 ± 0.002	0.56 ± 0.00	1.4 ± 0.1	1.1 ± 0.0	0.79 ± 0.01	5.2 ± 0.2	0.094 ± 0.006	
100	0.027	1.0	0.440 ± 0.005	0.63 ± 0.01	1.8 ± 0.2	1.3 ± 0.1	0.82 ± 0.01	6.1 ± 0.5	0.080 ± 0.014	N.D.[p]
150	0.018	1.0	0.425 ± 0.001	0.76 ± 0.03	2.5 ± 0.0	1.6 ± 0.0	0.86 ± 0.00	7.9 ± 0.1	0.063 ± 0.022	N.D.[p]
200	0.013	1.0	0.415 ± 0.000	0.96 ± 0.01	3.1 ± 0.0	2.0 ± 0.1	0.89 ± 0.01	9.6 ± 0.0	0.064 ± 0.009	N.D.[p]
300	0.0089	1.0	0.405 ± 0.003	1.1 ± 0.03	3.9 ± 0.3	2.4 ± 0.2	0.91 ± 0.01	11 ± 0.8	0.058 ± 0.011	N.D.[p]
**(B) PEG5K modified 20T at 5'-terminal (E-PEG5K-20T)** A[l] = 0.017 × 10^{-3}, B[m] = 12, |R[n]| = 1, D_0[o] = 4.4 × 10^{-11} m²/s										
70	0.038	0.05	0.404 ± 0.002	0.31 ± 0.02	1.3 ± 0.1	1.1 ± 0.0	0.99 ± 0.03	4.8 ± 0.1	0.033 ± 0.002	0.92
		0.10	0.405 ± 0.002	0.34 ± 0.02	1.3 ± 0.0	1.1 ± 0.0	0.99 ± 0.02	4.8 ± 0.1	0.042 ± 0.007	
		0.50	0.400 ± 0.000	0.44 ± 0.00	1.4 ± 0.0	1.1 ± 0.0	1.0 ± 0.00	5.1 ± 0.0	0.061 ± 0.001	
		1.0	0.400 ± 0.001	0.49 ± 0.00	1.4 ± 0.0	1.1 ± 0.0	1.1 ± 0.00	5.1 ± 0.0	0.074 ± 0.001	
100	0.027	1.0	0.393 ± 0.005	0.65 ± 0.03	1.7 ± 0.2	1.3 ± 0.0	1.2 ± 0.10	5.7 ± 0.5	0.10 ±0.03	N.D.[p]
150	0.018	1.0	0.379 ± 0.005	0.78 ± 0.00	2.3 ± 0.0	1.6 ± 0.1	1.5 ± 0.00	7.4 ± 0.0	0.078 ± 0.000	N.D.[p]
200	0.013	1.0	0.372 ± 0.001	1.1 ± 0.06	2.7 ± 0.0	1.8 ± 0.0	1.7 ± 0.00	8.5 ± 0.0	0.10 ± 0.010	N.D.[p]
300	0.0089	1.0	0.363 ± 0.001	1.2 ± 0.00	3.4 ± 0.1	2.3 ± 0.0	2.1 ± 0.05	10 ± 0.2	0.087 ± 0.011	N.D.[p]
**(C) PEG20K modified 20T at 5'-terminal (E-PEG20K-20T)** A[l] = 0.0063 × 10^{-3}, B[m] = 12, |R[n]| =1, D_0[o] = 2.7 × 10^{-11} m²/s										
70	0.038	0.05	0.371 ± 0.001	0.27 ± 0.02	1.4 ± 0.0	1.0 ± 0.0	0.78 ± 0.00	4.9 ± 0.0	0.025 ± 0.003	0.61
		0.10	0.370 ± 0.000	0.32 ±0.03	1.4 ± 0.0	1.0 ± 0.0	0.78 ± 0.00	4.9 ± 0.0	0.035 ± 0.007	
		0.50	0.370 ± 0.000	0.49 ± 0.00	1.4 ± 0.0	1.0 ± 0.0	0.78 ± 0.00	5.0 ± 0.1	0.080 ± 0.001	
		1.0	0.368 ± 0.002	0.71 ± 0.03	1.6± 0.0	1.0 ± 0.0	0.79 ± 0.00	5.1 ± 0.1	0.10 ± 0.00	
100	0.027	1.0	0.361 ± 0.001	0.84 ± 0.03	1.9 ± 0.1	1.2 ± 0.0	0.81 ± 0.00	5.7 ± 0.0	0.12 ± 0.01	N.D.[p]
150	0.018	1.0	0.355 ± 0.001	0.84 ± 0.09	2.6 ± 0.1	1.3 ± 0.0	0.83 ± 0.00	6.4 ± 0.1	0.13 ± 0.02	N.D.[p]
200	0.013	1.0	0.343 ± 0.001	1.0 ± 0.03	2.4± 0.1	1.7 ± 0.1	0.87 ± 0.01	8.3 ± 0.3	0.096 ± 0.003	N.D.[p]
300	0.0089	1.0	0.337 ± 0.002	1.2 ± 0.08	3.1 ± 0.2	2.0 ± 0.1	0.89 ± 0.00	9.6 ± 0.4	0.10 ± 0.00	N.D.[p]

(iii) unmodified and modified 30T

V_g[a] [mL]	GH[b] [M]	F[c] [mL/min]	I_R[d] [M]	σ_v[e] [mL]	K_R[f]	M[g]	L[h]	V_R[i] [mL]	$HETP_{LGE}$[j] [cm]	D_p[k] × 10^{11} [m^2/s]
**(A) NH_2 modified 30T at 5'-terminal (E-NH_2-30T)** A[l] = 0.013 × 10^{-3}, B[m] = 16, $\|R$[n]$\|$ = 1, D_0[o] = 4.8 × 10^{-11} m^2/s										
70	0.038	0.05	0.510 ± 0.000	0.38 ± 0.01	1.1 ± 0.0	0.89 ± 0.00	0.76 ± 0.01	4.3 ± 0.0	0.071 ± 0.004	0.93
		0.10	0.508 ± 0.000	0.44 ± 0.02	1.2 ± 0.0	0.93 ± 0.01	0.76 ± 0.00	4.4 ± 0.0	0.084 ± 0.009	
		0.50	0.504 ± 0.000	0.51 ± 0.03	1.3 ± 0.0	0.98 ± 0.01	0.78 ± 0.00	4.7 ± 0.0	0.10 ± 0.01	
		1.0	0.504 ± 0.001	0.58 ± 0.01	1.3 ± 0.0	0.98 ± 0.02	0.78 ± 0.00	4.7 ± 0.1	0.13 ± 0.01	
100	0.027	1.0	0.493 ± 0.001	0.71 ± 0.03	1.6 ± 0.2	1.2 ± 0.0	0.81 ± 0.00	5.5 ± 0.1	0.13 ± 0.01	N.D.[p]
150	0.018	1.0	0.482 ± 0.000	0.89 ± 0.04	2.1 ± 0.0	1.4 ± 0.0	0.84 ± 0.00	6.9 ± 0.1	0.12 ± 0.01	N.D.[p]
200	0.013	1.0	0.473 ± 0.000	1.1 ± 0.02	2.6 ± 0.0	1.7 ± 0.0	0.87 ± 0.00	8.3 ± 0.0	0.11 ± 0.00	N.D.[p]
300	0.0089	1.0	0.463 ± 0.001	1.3 ± 0.10	3.5 ± 0.1	2.3 ± 0.0	0.90 ± 0.00	11 ± 0.2	0.090 ± 0.019	N.D.[p]
**(B) PEG5K modified 30T at 5'-terminal (E-PEG5K-30T)** A[l] = 0.0030 × 10^{-3}, B[m] = 16, $\|R$[n]$\|$ = 1, D_0[o] = 3.8 × 10^{-11} m^2/s										
70	0.038	0.05	0.465 ± 0.000	0.30 ± 0.00	1.1 ± 0.0	0.90 ± 0.00	0.76 ± 0.00	4.3 ± 0.0	0.043 ± 0.000	0.57
		0.10	0.463 ± 0.001	0.34 ± 0.04	1.2 ± 0.0	0.92 ± 0.01	0.77 ± 0.00	4.4 ± 0.0	0.051 ± 0.001	
		0.50	0.460 ± 0.000	0.50 ± 0.02	1.3 ± 0.0	0.97 ± 0.00	0.77 ± 0.00	4.6 ± 0.0	0.099 ± 0.010	
		1.0	0.460 ± 0.001	0.57 ± 0.02	1.3 ± 0.0	0.97 ± 0.01	0.77 ± 0.00	4.6 ± 0.1	0.13 ± 0.01	
100	0.027	1.0	0.452 ± 0.000	0.70 ± 0.03	1.5 ± 0.2	1.1 ± 0.0	0.80 ± 0.00	5.3 ± 0.0	0.14 ± 0.01	N.D.[p]
150	0.018	1.0	0.441 ± 0.000	0.85 ± 0.03	2.0 ± 0.0	1.4 ± 0.0	0.84 ± 0.00	6.6 ± 0.1	0.12 ± 0.01	N.D.[p]
200	0.013	1.0	0.434 ± 0.000	1.0 ± 0.02	2.4 ± 0.0	1.6 ± 0.0	0.86 ± 0.00	7.7 ± 0.1	0.12 ± 0.01	N.D.[p]
300	0.0089	1.0	0.425 ± 0.001	1.2 ± 0.03	3.1 ± 0.1	2.0 ± 0.0	0.89 ± 0.00	9.7 ± 0.2	0.096 ± 0.010	N.D.[p]
**(C) PEG20K modified 30T at 5'-terminal (E-PEG20K-30T)** A[l] = 0.0011 × 10^{-3}, B[m] = 16, $\|R$[n]$\|$ = 1, D_0[o] = 2.5 × 10^{-11} m^2/s										
70	0.038	0.05	0.436 ± 0.000	0.26 ± 0.01	1.1 ± 0.0	0.90 ± 0.00	0.76 ± 0.01	4.3 ± 0.0	0.033 ± 0.004	0.53
		0.10	0.434 ± 0.000	0.32 ± 0.00	1.2 ± 0.0	0.91 ± 0.00	0.76 ± 0.00	4.4 ± 0.0	0.045 ± 0.009	
		0.50	0.432 ± 0.001	0.45 ± 0.05	1.2 ± 0.0	0.95 ± 0.01	0.77 ± 0.00	4.5 ± 0.1	0.084 ± 0.016	
		1.0	0.431 ± 0.001	0.57 ± 0.04	1.2 ± 0.0	0.96 ± 0.01	0.77 ± 0.00	4.6 ± 0.1	0.13 ± 0.01	
100	0.027	1.0	0.423 ± 0.001	0.61 ± 0.02	1.5 ± 0.2	1.1 ± 0.0	0.80 ± 0.00	5.3 ± 0.1	0.10 ± 0.01	N.D.[p]
150	0.018	1.0	0.416 ± 0.000	0.76 ± 0.06	1.8 ± 0.0	1.3 ± 0.0	0.82 ± 0.00	6.1 ± 0.0	0.11 ± 0.02	N.D.[p]
200	0.013	1.0	0.407 ± 0.002	0.93 ± 0.02	2.4 ± 0.2	1.6 ± 0.1	0.86 ± 0.01	7.7 ± 0.5	0.10 ± 0.01	N.D.[p]
300	0.0089	1.0	0.403 ± 0.001	1.1 ± 0.0	2.7 ± 0.1	1.8 ± 0.0	0.87 ± 0.00	8.6 ± 0.1	0.11 ± 0.01	N.D.[p]

(iv) unmodified and modified 50T

V_g[a] [mL]	GH[b] [M]	F[c] [mL/min]	I_R[d] [M]	σ_v[e] [mL]	K_R[f]	M[g]	L[h]	V_R[i] [mL]	$HETP_{LGE}$[j] [cm]	D_p[k] × 10^{11} [m^2/s]
**(A) NH_2 modified 50T at 5'-terminal (E-NH_2-50T)** A[l] = 0.0028 × 10^{-3}, B[m] = 21, |R[n]| = 0.997, D_0[o] = 3.9 × 10^{-11} m^2/s										
70	0.038	0.05	0.559 ± 0.001	0.30 ± 0.00	1.1 ± 0.0	0.92 ± 0.01	0.76 ± 0.00	4.3 ± 0.0	0.042 ± 0.004	0.55
		0.10	0.559 ± 0.001	0.35 ± 0.01	1.1 ± 0.0	0.92 ± 0.02	0.76 ± 0.00	4.4 ± 0.1	0.056 ± 0.009	
		0.50	0.564 ± 0.002	0.43 ± 0.00	1.0 ± 0.0	0.86 ± 0.02	0.75 ± 0.01	4.0 ± 0.1	0.10 ± 0.01	
		1.0	0.560 ± 0.003	0.53 ± 0.01	1.1 ± 0.1	0.91 ± 0.05	0.76 ± 0.01	4.2 ± 0.2	0.14 ± 0.01	
100	0.027	1.0	0.550 ± 0.000	0.67 ± 0.01	1.4 ± 0.0	1.1 ± 0.0	0.80 ± 0.00	5.1 ± 0.0	0.14 ± 0.01	N.D.[p]
150	0.018	1.0	0.541 ± 0.002	0.81 ± 0.01	1.8 ± 0.1	1.3 ± 0.1	0.83 ± 0.01	6.1 ± 0.3	0.13 ± 0.01	N.D.[p]
200	0.013	1.0	0.533 ± 0.002	0.89 ± 0.01	2.3 ± 0.1	1.6 ± 0.1	0.86 ± 0.01	7.5 ± 0.3	0.096 ± 0.00	N.D.[p]
300	0.0089	1.0	0.525 ± 0.000	1.4 ± 0.0	3.0 ± 0.0	2.1 ± 0.0	0.89 ± 0.00	9.5 ± 0.0	0.13 ± 0.019	N.D.[p]
**(B) PEG5K modified 50T at 5'-terminal (E-PEG5K-50T)** A[l] = 0.00070 × 10^{-3}, B[m] = 21, |R[n]| = 1, D_0[o] = 3.1 × 10^{-11} m^2/s										
70	0.038	0.05	0.524 ± 0.001	0.25 ± 0.01	1.1 ± 0.0	0.93 ± 0.01	0.77 ± 0.00	4.3 ± 0.1	0.030 ± 0.001	0.52
		0.10	0.524 ± 0.002	0.30 ± 0.01	1.1 ± 0.0	0.93 ± 0.03	0.77 ± 0.01	4.3 ± 0.1	0.042 ± 0.000	
		0.50	0.529 ± 0.001	0.42 ± 0.01	1.0 ± 0.0	0.86 ± 0.02	0.75 ± 0.00	4.0 ± 0.1	0.099 ± 0.001	
		1.0	0.525 ± 0.003	0.52 ± 0.01	1.1 ± 0.1	0.91 ± 0.02	0.76 ± 0.01	4.2 ± 0.3	0.13 ± 0.02	
100	0.027	1.0	0.516 ± 0.001	0.66 ± 0.05	1.4 ± 0.0	1.1 ± 0.0	0.80 ± 0.00	5.0 ± 0.1	0.14 ± 0.02	N.D.[p]
150	0.018	1.0	0.509 ± 0.003	0.78 ± 0.03	1.7 ± 0.2	1.3 ± 0.1	0.82 ± 0.02	5.9 ± 0.5	0.13 ± 0.02	N.D.[p]
200	0.013	1.0	0.503 ± 0.001	0.83 ± 0.04	2.1 ± 0.6	1.5 ± 0.0	0.85 ± 0.00	6.9 ± 0.2	0.10 ± 0.01	N.D.[p]
300	0.0089	1.0	0.495 ± 0.000	1.2 ± 0.0	2.8 ± 0.0	1.9 ± 0.0	0.88 ± 0.00	8.7 ± 0.0	0.11 ± 0.00	N.D.[p]
**(C) PEG20K modified 50T at 5'-terminal (E-PEG20K-50T)** A = 0.00027 × 10^{-3}, B = 21, |R| = 1, D_0[o] = 2.3 × 10^{-11} m^2/s										
70	0.038	0.05	0.501 ± 0.001	0.25 ± 0.03	1.1 ± 0.0	0.92 ± 0.02	0.76 ± 0.00	4.3 ± 0.1	0.028 ± 0.005	0.43
		0.10	0.449 ± 0.001	0.29 ± 0.01	1.2 ± 0.0	0.96 ± 0.02	0.77 ± 0.00	4.5 ± 0.1	0.034 ± 0.000	
		0.50	0.498 ± 0.001	0.44 ± 0.02	1.2 ± 0.0	0.99 ± 0.02	0.78 ± 0.00	4.6 ± 0.1	0.078 ± 0.009	
		1.0	0.506 ± 0.000	0.51 ± 0.01	1.0 ± 0.0	0.85 ± 0.03	0.75 ± 0.00	3.9 ± 0.1	0.15 ± 0.01	
100	0.027	1.0	0.493 ± 0.000	0.65 ± 0.01	1.4 ± 0.0	1.1 ± 0.0	0.80 ± 0.00	5.1 ± 0.1	0.13 ± 0.00	N.D.[p]
150	0.018	1.0	0.486 ± 0.000	0.81 ± 0.05	1.7 ± 0.0	1.3 ± 0.0	0.82 ± 0.00	6.0 ± 0.0	0.14 ± 0.01	N.D.[p]
200	0.013	1.0	0.481 ± 0.000	0.88 ± 0.05	2.1 ± 0.0	1.5 ± 0.0	0.85 ± 0.00	6.8 ± 0.1	0.12 ± 0.02	N.D.[p]
300	0.0089	1.0	0.475 ± 0.001	1.2 ± 0.0	2.6 ± 0.1	1.8 ± 0.1	0.87 ± 0.01	8.3 ± 0.3	0.13 ± 0.00	N.D.[p]

[a]V_g: Gradient volume from the initial salt concentration (I_0 = 0.030 M) to the final salt concentration (I_f = 1.0 M) in a linear salt gradient elution experiment.

[b]GH: Normalized gradient slope, calculated as $GH = \frac{I_f - I_0}{V_g / V_t(1-\varepsilon)}$, where V_t (= 3.9 mL) is the total column volume and V_s (= 2.7 mL) is the stationary phase volume (i. e. $V_t(1-\varepsilon)$).

[c]F: flow rate

[d]I_R: Peak elution salt concentration.

[e]σ_V: Volume-based standard deviation of the peak.

[f]K_R: Distribution coefficient as a function of the mobile phase salt concentration, defined in **Equation (4)**.

[g]M: Dimensionless group described in **Equation (7)**.

[h]L: Correction factor for HETP in linear gradient elution, defined in **Equation (2)**.

[i]V_R: Retention volume, as defined in **Equation (8)**.

[j]$HETP_{LGE}$: Height equivalent to a theoretical plate, corrected by L, as defined in **Equation (5)**.

[k]D_p:Intraparticele diffusivity estimated from the C term of van Deemter equation as follow[S2, S3]; .

$$HETP = A° + B°/u + C°u = A° + \left(\frac{d_p^2 H K_R}{30(1+HK_R)^2 D_P}\right)u$$

where $A°$ is the intercept in van Deemter plot, $B°$ represents longitudinal diffusion, u is interstitial velocity, dp is the diameter of particle and H is the phase ratio.

[l]A: Fitting parameter of the GH-IR model described in **Equation (2)**.

[m]B: Number of the binding sites of the solute to the stationary phase.

[g]$|R|$: Correlation coefficient obtained from the GH-I_R plots (**Equation (2))**.

[p] N.D.: Not determined.

8. MD simulations for local interaction tendencies between PEG chains and DNA functional groups.

MD simulations were performed to characterize local interaction tendencies between PEG chains and DNA functional groups at the atomic level. The simulations were not intended to reproduce detailed DNA conformational dynamics or long-timescale structural fluctuations, but rather to compare relative interaction propensities among phosphate, sugar, and nucleobase moieties under identical conditions.

Initial structures of PEGylated 9T with C6 amino linked PEG (PEG 10mer, M_w around 0.6 kDa) were constructed using Avogadro[S3] (version 1.2.0) and Avogadro 2 (version 1.102.1). Molecular dynamics simulations were performed using GROMACS[S4,S5] (version 2025.4.). To reduce the computational cost, the PEG chain was truncated to 10 ethylene glycol units (approximately 0.6 kDa), including the C6 amino linker, which is comparable in counter length to the 9T oligonucleotide.

The simulations were carried out in explicit water solvent containing 0.15 M NaCl. All molecular models were described using GAFF[6]-based parameters generated using ACPYPE[7], and water molecules were modeled using the TIP3P model[8]. Periodic boundary conditions were applied in all directions. A time step of 2fs was used, and all bonds involving hydrogen atoms were constrained using the LINCS algorithm[9]. Short-range electrostatic and van der Waals interactions were truncated at 1.0 nm, and long-range electrostatic interactions were treated using the particle mesh Ewald (PME) method[10].

After energy minimization, the systems were equilibrated under NVT and NPT ensembles at 300 K and 1 atm. Temperature was controlled using the V-rescale thermostat[11], and pressure was controlled using the Parrinello-Rahman barostat[12]. All simulations were performed for 50 ns.

Contact analysis between PEG and DNA was performed using the gmx mindist tool in GROMACS. The DNA strand was divided into nine thymidine residues (DT1 – DT9), and each residue was further classified into three substructures: phosphate group, sugar moiety, and nucleobase. For each DNA substructure, the minimum distance to PEG atoms was calculated at every frame of the 50-ns trajectory. A contact was defined when the minimum distance was shorter than 0.35 nm. The contact frequency was calculated as the fraction of frames satisfying this condition. In addition, the mean minimum distance and its standard deviation were calculated over the entire MD trajectory.

(A) PEGylated 9T modified with PEG 0.6 kDa at the 5' -end (E-PEG-9T)

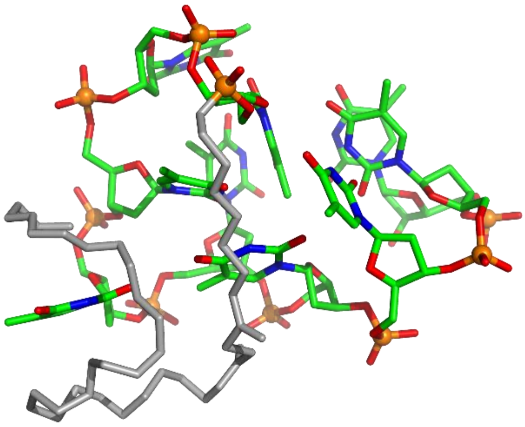

(B) PEGylated 9T modified with PEG 0.6 kDa at the 5th thymine (M-PEG-9T)

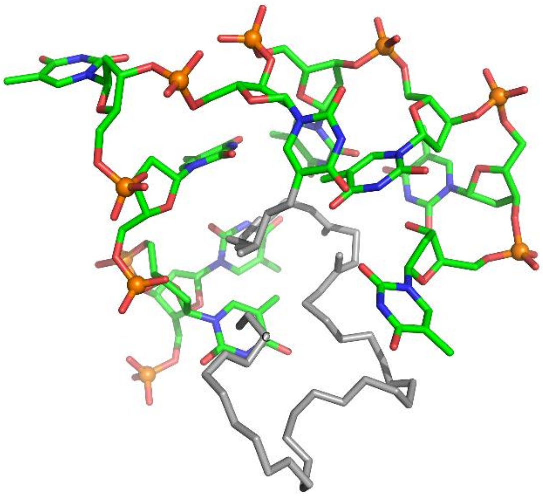

Figure S21. Representative structures of unmodified and PEGylated 9T obtained after 50 ns MD simulations.

(A) PEGylated 9T modified with PEG 0.6 kDa at the 5' -end (E-PEG-9T).

(B) PEGylated 9T modified with PEG 0.6 kDa at the 5th thymine (M-PEG-9T).

Carbon, nitrogen, oxygen and phosphate atoms of 9T are shown in green, blue, red, and orange spheres, respectively. The PEG chain is shown in gray. Hydrogen atoms are omitted for clarity.

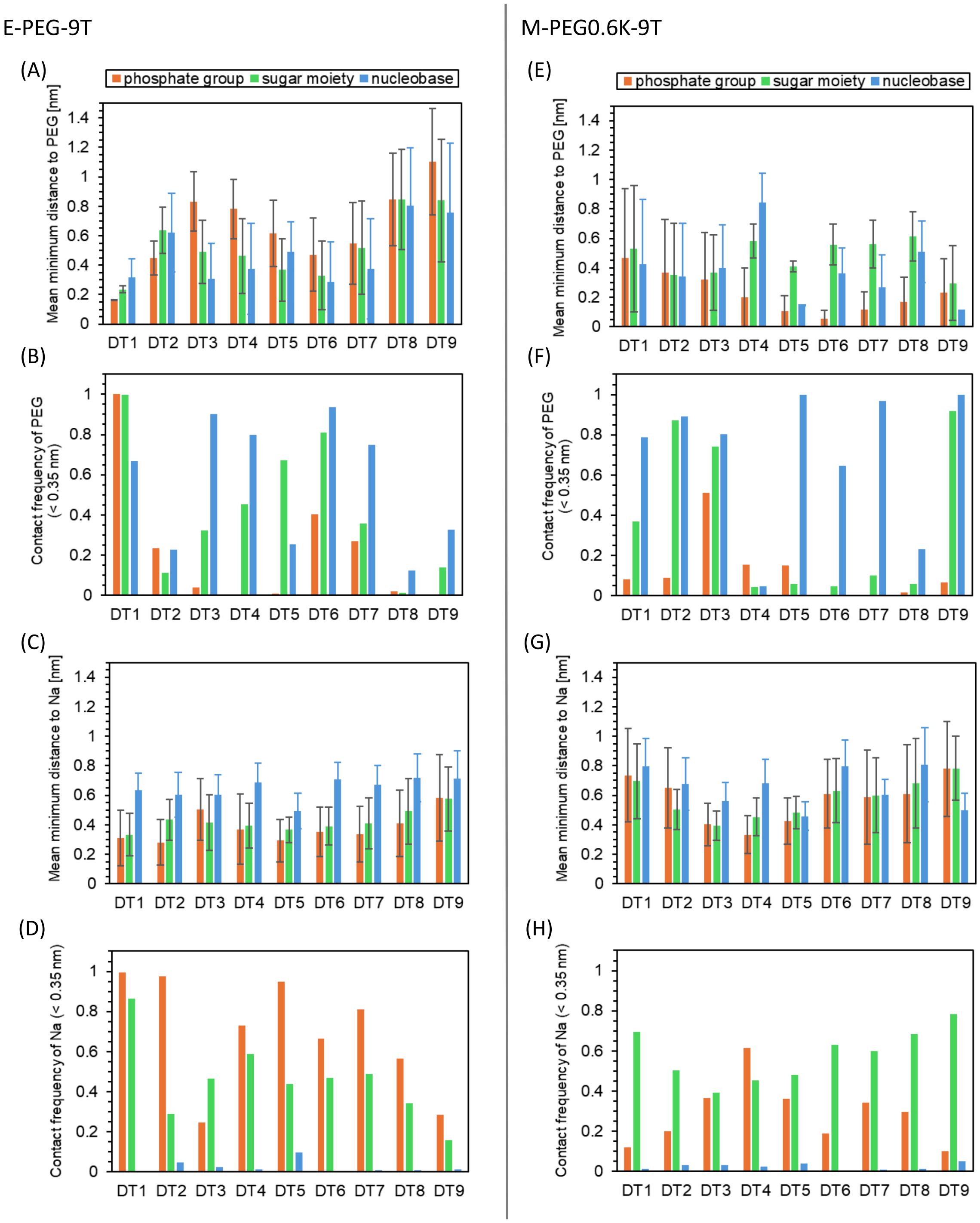

Figure S22. Minimum distances and contact frequencies between PEG chains or Na^+ ions and functional groups of poly(9T) analyzed by molecular dynamics simulations.

PEGylated 9T modified with PEG 0.6 kDa at the 5' -end (E-PEG-9T):

(A) Mean minimal distance to the PEG chain. (B) Contact frequency of the PEG chain. (C) Mean minimal distance to Na^+ ions, (D) Contact frequency of Na^+ ions.

PEGylated 9T modified with PEG 0.6 kDa at the 5th thymine (M-PEG-9T):

(E) Mean minimal distance to the PEG chain. (F) Contact frequency of the PEG chain. (G) Mean minimal distance to Na^+ ions. (H) Contact frequency of Na^+ ions.

References

S1.1. Fee, C. J., & Van Alstine, J. M. (2004). Prediction of the viscosity radius and the size exclusion chromatography behavior of PEGylated proteins. *Bioconjugate Chemistry*, *15*(6), 1304–1313. https://doi.org/10.1021/bc049843w

2. Chen, C. S., Simoes-Cardoso, J. C., Ikezuki, Y., Tanaka, D., Yoshimoto, N., & Yamamoto, S. (2020). Retention and diffusion characteristics of oligonucleotides in a solid phase with polymer grafted anion-exchanger. *Journal of Chromatography A*, *1629*, 461495. https://doi.org/10.1016/j.chroma.2020.461495

3. Hanwell, M. D., Curtis, D. E., Lonie, D. C., Vandermeersch, T., Zurek, E., & Hutchison, G. R. (2018). Avogadro: an advanced semantic chemical editor, visualization, and analysis platform | Journal of Cheminformatics | Full Text. *Journal of Cheminformatics*, *4*, 1–17. https://jcheminf.springeropen.com/articles/10.1186/1758-2946-4-17%0Ahttp://files/1718/1758-2946-4-17.html

4. Van Der Spoel, D., Lindahl, E., Hess, B., Groenhof, G., Mark, A. E., & Berendsen, H. J. C. (2005). GROMACS: Fast, flexible, and free. *Journal of Computational Chemistry*, *26*(16), 1701–1718. https://doi.org/10.1002/jcc.20291

5. Abraham, M. J., Murtola, T., Schulz, R., Páll, S., Smith, J. C., Hess, B., & Lindah, E. (2015). Gromacs: High performance molecular simulations through multi-level parallelism from laptops to supercomputers. *SoftwareX*, *1–2*, 19–25. https://doi.org/10.1016/j.softx.2015.06.001

6. Wang, J., Wolf, R. M., Caldwell, J. W., Kollman, P. A., & Case, D. A. (2004). Development and testing of a general Amber force field. *Journal of Computational Chemistry*, *25*(9), 1157–1174. https://doi.org/10.1002/jcc.20035

7. Sousa Da Silva, A. W., & Vranken, W. F. (2012). ACPYPE - AnteChamber PYthon Parser interfacE. *BMC Research Notes*, *5*, 1–8. https://doi.org/10.1186/1756-0500-5-367

8. Klein, W. L. J. J. C. J. D. M. R. W. I. M. L. (1983). Comparison of simple potential functions for simulating liquid water. *The Journal of Chemical Physics*, *79*(2), 926–935. https://doi.org/https://doi.org/10.1063/1.445869

9. Hess, B., Bekker, H., Berendsen, H. J. C., & Fraaije, J. G. E. M. (1997). LINCS: A Linear Constraint Solver for molecular simulations. *Journal of Computational Chemistry*, *18*(12), 1463–1472. https://doi.org/10.1002/(SICI)1096-987X(199709)18:12<1463::AID-JCC4>3.0.CO;2-H

10. Darden, T., York, D., & Pedersen, L. (1993). Particle mesh Ewald: An N·log(N) method for Ewald sums in large systems. *The Journal of Biochemistry*, *98*(12), 10089–10092. https://doi.org/https://doi.org/10.1063/1.464397

11. Bussi, G., Donadio, D., & Parrinello, M. (2007). Canonical sampling through velocity rescaling. *Journal of Chemical Physics*, *126*(1). https://doi.org/10.1063/1.2408420

12. Parrinello, M., & Rahman, A. (1981). Polymorphic transitions in single crystals: A new molecular dynamics method. *Journal of Applied Physics*, *52*(12), 7182–7190. https://doi.org/10.1063/1.328693
